# Wildfire exposure and political participation in Africa

**DOI:** 10.1038/s41467-026-77404-4

**Published:** 2026-09-12

**Authors:** Siyuan Chen, Michael Akwasi Appiah-Kubi, Lina Meng, Pengfei Liu, Lu Lin

**Affiliations:** 1 School of Economics, Xiamen University, Xiamen, Fujian China; 2 MAFLO African Urban Research Center, Achimota-Ofankor, Accra Ghana; 3 Wang Yanan Institute for Studies in Economics (WISE), Xiamen University, Xiamen, Fujian China; 4 Key Laboratory of Urban Land Resources Monitoring and Simulation, Ministry of Natural Resource, Shenzhen, Guangdong China; 5 School of Economics and Management, Beihang University, Haidian, Beijing China; 6 Lab for Low-carbon Intelligent Governance (LLIG), Beihang University, Haidian, Beijing China; 7 School of Economics and Management, China University of Petroleum Beijing, Changping, Beijing China

**Keywords:** Economics, Developing world

## Abstract

Civic engagement can shape collective responses to climate change. However, evidence on how climate-related disasters affect political participation in Africa remains limited. Combining Global Fire Atlas records with Afrobarometer surveys of more than 100,000 people in 34 African countries, we show that people living close to wildfires are more likely to vote, contact local councillors, attend community meetings, and raise collective issues, whereas protest participation shows weaker responses. These effects are larger in arid regions and among people with higher climate literacy or access to information, members of politically dominant ethnic groups and those living near ethnic boundaries. Our findings suggest that participation is motivated mainly by immediate material loss and demand for public services, rather than environmental concern. These findings highlight the importance of addressing post-disaster needs to sustain political engagement and public support for long-term climate adaptation in the Global South.

## Abstract (web summary)

Wildfire exposure in Africa increases voting, contacting local officials, attending community meetings, and raising collective issues, driven by material deprivation and demand for government response rather than environmental concern, according to an analysis of over 100,000 individuals across 34 countries.

## Introduction

Climate change is increasing the incidence of extreme weather events with wide-ranging adverse socioeconomic consequences^1^. Globally, the incidence of extreme wildfires, defined as those above the 99th percentile in size, increased to 2.2 times its 2003 level by 2023^2^. Africa contains some of the world’s most fire-prone ecosystems. During 1996–2011, annual burned area averaged 117.7 million hectares in Northern Hemisphere sub-Saharan Africa and 125.0 million hectares in Southern Hemisphere Africa^3^, together accounting for roughly 70% of the global total^4,5^. With wildfire risk expected to escalate^6^, Africa provides a distinctive setting in which to examine whether climate shocks generate political responses different from those observed in developed economies.

Understanding how climate-induced disasters shape political participation in Africa requires considering the continent’s institutional and political context. Research on climate extremes has primarily examined electoral behaviour and environmental attitudes in developed democracies^7–11^. In Africa, however, citizens often evaluate democracy instrumentally, assessing it by its capacity to deliver immediate, tangible benefits rather than by ideological commitments^12–14^, which may shape political mobilisation after disasters. Moreover, the literature’s emphasis on elections overlooks community-level civic engagement, often the principal means through which African citizens influence governance and express demands^15,16^. Unlike national elections, which are infrequent, community activities such as contacting local officials, attending meetings, and raising collective issues provide ongoing opportunities for citizens to seek redress for immediate concerns. In Africa, with around 70% of the population under 30 and many communities facing material deprivation and weak institutional capacity, local governance mechanisms are vital channels for grass-roots democracy. The significance of community participation also varies across different African contexts, shaped by decentralisation frameworks, ethnic compositions, and institutional capacities, with countries like South Africa, Kenya, Ghana, and Rwanda developing distinct local governance innovations that establish formal channels for disaster-affected communities to articulate demands^17–19^.

We therefore develop a framework in which material deprivation and government responsiveness are the main links between wildfire exposure and political participation. This differs from Global North models in which climate disasters heighten environmental concern and support for climate-friendly policies^8,9,20,21^. In settings characterised by limited climate literacy, immediate material scarcity, and instrumental orientations toward democracy, wildfires mobilise citizens primarily through disruption of daily life and creation of urgent needs for government response^22–24^. Climate-induced wildfires generate immediate material consequences, creating pressing needs that cannot be addressed through individual coping strategies alone. Where democracy is valued instrumentally as a means to securing tangible benefits, material deprivation creates powerful incentives for political mobilisation^12,13^. Citizens engage in community-level political activities not primarily from ideological commitment to environmental protection, but as strategic responses to immediate threats to well-being. Our framework distinguishes between institutionalised participation (voting, contacting councillors, community meetings), representing low-cost activities within existing governance structures, and non-institutionalised participation (collective issue-raising, protests) involving higher costs but necessary when institutionalised channels prove unresponsive^25^. Political responses vary systematically across populations based on factors affecting both the severity of material losses and the capacity for mobilisation, including climate vulnerability, ethnic political status, information accessibility, and socioeconomic resources^26,27^. By centreing community-level participation and theorising material deprivation and government’s bundled reactions as primary mechanisms, we extend understanding of how citizens in the Global South respond to climate extremes through diverse forms of civic engagement.

Empirically, we examine how large fires affect voting, contacting local government officials, attending community meetings, collective issue-raising, and protest. By combining wildfire data from the Global Fire Atlas with Afrobarometer survey data from rounds 3 to 6 covering over 100,000 individuals across 34 African countries between 2005 and 2015^28,29^, we offer a continent-wide analysis of the relationship between wildfire and political engagement. Using an instrumented linear probability model (LPM), we find that wildfire exposure is associated with a statistically significant increase in the probability of individuals participating in multiple forms of political activities. Wildfire-induced material deprivation and government responsiveness are key mechanisms and substantially increase political participation in affected villages.

Our study contributes to the literature in three ways. First, it provides continent-wide evidence from Africa, a highly climate-vulnerable but under-represented region, that wildfire exposure increases voting, contact with officials, community-meeting attendance and collective issue-raising. Prior work has largely examined how climate shocks, such as precipitation and temperature anomalies, affect environmental concern and green voting in developed countries^7–11^, while concentrating less on non-electoral activities in under-developed countries. As African climate risks intensify, communities’ capacity to engage politically and articulate collective demands is central to climate resilience and institutional accountability. The African context is especially important for the United Nations Sustainable Development Goals (SDGs), particularly the continent facing disproportionate climate risks (SDG 13 on Climate Action) and challenges in institutional responsiveness (SDG 16 on Peace, Justice, and Strong Institutions).

Second, our study contributes to a growing body of research examining the socioeconomic consequences of climate change in Africa. The existing literature has documented a wide array of impacts, including rising income and wealth inequality^30^, increasing regional polarisation^31^, intensifying conflict^32,33^, worsening infant mortality^34^, and declining social trust^35^. While such disruptions can plausibly motivate political engagement, it remains unclear whether—and through which pathways—climate-related shocks trigger political participation in African contexts^36^. Consistent with Africans’ instrumental preference for democracy and emphasis on near-term benefits^12,13^, we find no increase in general political interest or change in interethnic relations. Instead, wildfire exposure drives political participation primarily by creating shortages of basic necessities, suggesting that climate-related disasters mobilise citizens through immediate economic hardship, needs for public goods, and the disruption of daily life, instead of ideological or environmental awakening. This contrasts with advanced democracies, where such events mainly shift environmental attitudes, climate perceptions, and underlying political inclinations^23,37^.

Third, our comprehensive individual-level data reveal how exposure to extreme climatic perturbations differentially affects political engagement across demographic groups, capturing intra-population diversity obscured by the aggregate country- or region-level data commonly used to study participation^9,12,38–42^. We examine heterogeneous post-wildfire responses using proxies for information accessibility and demographic characteristics, including gender, urban–rural residence, education, occupation, and housing attributes. The results provide a more holistic account of popular political engagement following exogenously induced adverse shocks and can guide targeted, effective interventions responsive to communities’ differing needs and capacities.

## Results

**Fig. 1:**
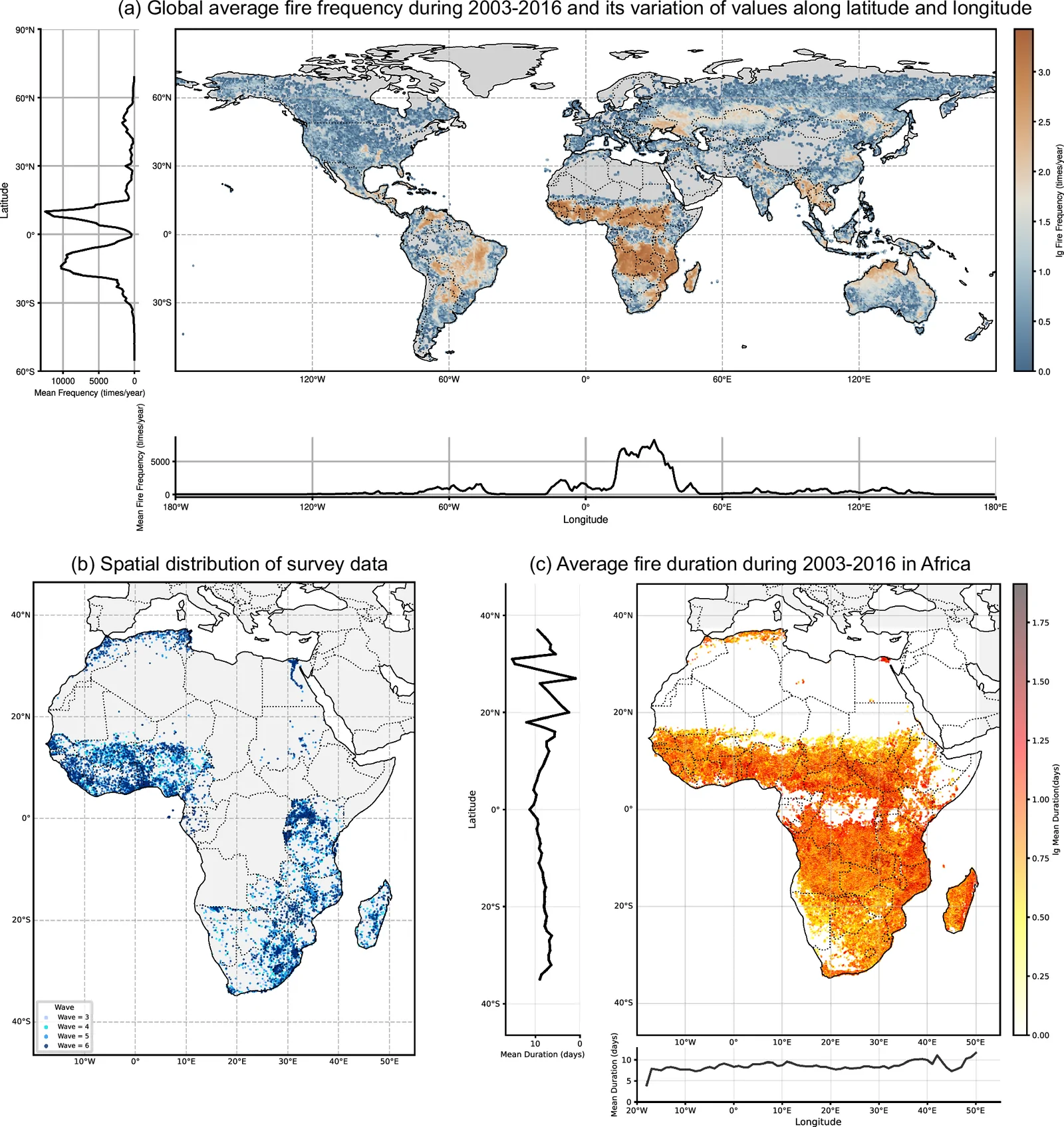
Distribution of large fires and geocoded survey data from 2003–2016. **a** Global average fire frequency from 2003–2016 and variation along latitude and longitude. Darker colours indicate higher fire frequency. **b** Spatial distribution of Afrobarometer survey respondents, the data is geocoded to the village they lived; *n* = 12, 783 villages. **c** Average large-fire duration in Africa from 2003–2016, Areas with darker colour indicate regions in Africa with longer average fire duration. Fire data are from the Global Fire Atlas; only fires exceeding 500 acres are retained. The survey data on political participation comes from Afrobarometer Rounds 3 to 6, covering 34 countries in Africa^29^. The fire data is sourced from Global Fire Atlas^4^. Figures were generated in Python and assembled using Inkscape. Country boundary data were sourced from Natural Earth.

To distinguish the impact of large-scale wildfire disasters from smaller fires typically associated with slash-and-burn agriculture^9,43^, we restrict the analysis to fires with burned areas exceeding 500 acres. Large-scale wildfire occurs on all continents, with a higher frequency in Sub-Saharan Africa, South America, and Northern Australia (Fig. 1a). Within Africa, the distribution of large-scale wildfires is uneven. More large-scale wildfires occur in the tropical belt of sub-Saharan Africa, with an average duration of 8–11 days (Fig. 1c). The duration of wildfires in other African regions is generally less than 10 days. Figure 1b shows the distribution of more than 100,000 geocoded Afrobarometer respondents from Rounds 3–6 across both fire-affected and unaffected regions.

### Wildfire exposure boosts political participation

Firstly, we estimate an LPM with country-year fixed effects in Equation (3) to examine the effect of wildfire exposure on political participation in Africa. To capture both conventional and contentious engagement, we distinguish institutionalised forms of participation, including voting, contacting local government officials, and attending community meetings, from non-institutionalised forms, including collective issue-raising and protest. We define the treatment group as the respondents living in villages located within 5 miles of the nearest wildfire in the two years preceding the survey. To reduce confounding from geocoding errors and create a clearer separation between exposed and unexposed locations, the control group comprises respondents living 7–20 miles from the nearest wildfire^44^. Panel A of Table 1 reports the OLS estimates, which show that respondents living closer to wildfires generally participate more frequently in political activities.

**Table 1:** Baseline Effects of Wildfire Exposure on Political Participation

	Institutionalised Form			Non-institutionalised Form	
Dept. Vars.	Voting	Contacting local govt.	Attending com. meetings	Collective issue-raising	Protest
	(1)	(2)	(3)	(4)	(5)
Panel A: OLS Estimation					
Wildfire	0.0045	0.0433***	0.0668***	0.0486***	0.0127
	(0.0045)	(0.0145)	(0.0183)	(0.0156)	(0.0123)
Observations	69,940	83,206	83,206	83,206	83,206
Panel B: 2SLS Estimation					
Wildfire	0.0728**	0.1490*	0.3750***	0.2080**	0.0373
	(0.0290)	(0.0767)	(0.0975)	(0.0830)	(0.0683)
Observations	69,940	83,206	83,206	83,206	83,206
Hansen J Statistic	2.315	1.909	3.049	2.971	1.156
First Stage F Statistic	26.74	31.44	31.44	31.44	31.44
Country-by-Year FE	Yes	Yes	Yes	Yes	Yes
Spatial Cut-off (miles)	20	20	20	20	20
Temporal Cut-off (months)	24	24	24	24	24

Note: Panel A reports the OLS estimation results by Equation (3), panel B reports the 2SLS estimation results based on Equation (5). We instrument ${{{\rm{Wildfire}}}}_{v(i),t-2}$ by **WildfireRisk**_*v*(*i*),*t*−2_, constructed by climate conditions in the last two years and land cover combustibility in the 3rd year prior to the interview year. F Statistic (one-sided) reports the result of the joint F test of excluded instrumental variables (**WildfireRisk**_*v*(*i*),*t*−2_) in the first stage regression, while Hansen J Statistic (two-sided) reports the over-identification test result of 2SLS estimation. The variable Wildfire is a binary indicator equal to one if a wildfire occurred within 5 miles of the respondent’s village in the two years preceding the survey, and zero if the nearest wildfire occurred between 7 miles and 20 miles during the same period. The non-binary dependent variables, including contacting local government officials, attending community meetings, collective issue-raising, and participation in protests, are standardised before estimation. All specifications include country-by-year fixed effects to account for unobserved heterogeneity and national-level temporal trends. Coefficients inference is based on two-sided tests with country-by-year fixed effects and robust standard errors clustered at the subnational-region level. Family-wise results were additionally evaluated using the Holm–Bonferroni correction reported in Supplementary Table 7. *** *p *< 0.01, ** *p *< 0.05, * *p *< 0.1.

However, this finding is difficult to interpret as a causal effect (see estimation method part of the Method section). To mitigate potential endogeneity, we construct an instrumental variable (IV) for wildfire proximity using meteorological and land cover data. Research demonstrates that exogenous climate factors, specifically temperature, precipitation, wind speed, and land cover, are key determinants of wildfire incidence^22,45^. We thus construct a wildfire risk index instrumented for the wildfire exposure index by interacting the annual average temperature and precipitation with lagged land-cover combustibility, and wind speed (As calculated by Equation (4)). The two-stage least squares (2SLS) specification controls separately for the direct effects of temperature, precipitation, land-cover combustibility, and country-year fixed effects. The identifying assumption is that, conditional on these controls, the interaction terms and wind speed affect political participation primarily through wildfire exposure rather than through other weather- or land-cover-related channels.^46,47^.

Panel B of Table 1 indicates that wildfire exposure increases both institutionalised and non-institutionalised forms of political participation, ranging from voting to collective-issue raising, but not protest. Relative to respondents living 7–20 miles from a recent wildfire, those within five miles are 7.3 percentage points more likely to vote (95% CI: 0.016–0.130). Proximity to a wildfire increases citizens’ reported frequency of contacting local councillors by 14.9% (95%CI: -0.002–0.300) of the standard deviations (S.D. = 0.89), which also boosts community meetings attendance by 37.5% (95%CI: 0.183–0.566) of the overall variation (S.D. = 1.31), and raises joint issues-setting by 20.8% (95% CI: 0.045–0.371) of the overall variation (S.D. = 1.29). In contrast, the effect on protest participation is insignificant. Protest may entail confrontation with authorities or social backlash and therefore impose greater personal risks than lower-cost activities such as voting or attending meetings.

The OLS estimates are generally smaller than their 2SLS counterparts, although both specifications yield effects in the same direction and neither identifies a clear average effect on protest. We treat the IV results as our preferred estimates. The baseline findings remain statistically significant after the Holm–Bonferroni correction for multiple comparisons^48^ (Supplementary Table 7) and remain stable when using alternative exposure measures, distance thresholds, and continuous-distance specifications; excluding agricultural and built-up land; controlling for higher-order temperature and precipitation terms; and adding individual, information-access, and transportation-access controls (Supplementary Tables 8–17). First-stage and reduced-form estimates, falsification tests, and over-identification tests further support the IV design (Supplementary Tables 18–20 and Table 1). The conclusions are also robust to an alternative ordered instrumented probit model (Supplementary Table 21).

### Mechanism analysis

Climate-related shocks can undermine individuals’ living conditions and generate economic losses^31,49,50^, which may exacerbate social tensions, highlight the necessity of government aid^24^ and sometimes trigger conflict and crime^32,51,52^, thereby influencing political participation. Direct exposure may also enhance awareness of climate risks^8,53^, potentially increasing environmental concern and, in turn, the willingness to participate in collective action. To distinguish these channels, we regress measures of material deprivation, perceived government performance, conflict exposure, and public concern on instrumented wildfire exposure using Equation (5).

Panel A of Table 2 shows that individuals living within 5 miles of recent wildfires are significantly more likely to experience medicine and income deprivations than those farther away (Columns (1)–(4)). Notably, wildfire exposure also increases respondents’ favourable evaluations of local councillors’ performance (Column (5) of Panel A), a pattern consistent with greater demand for public goods and local government responsiveness following climate-related shocks^10^.

**Table 2:** Mechanism analysis

Panel A: Material Deprivation and Government Performance					
	Material Deprivation				Percep. Local Govt.
Dep. Vars.	Without Food	Without Income	Without Medics	Without Water	Local Govt. Perf.
	(1)	(2)	(3)	(4)	(5)
Wildfire	-0.0163	0.1640*	0.2450**	0.1800	0.1750**
	(0.1110)	(0.0970)	(0.0961)	(0.1210)	(0.0837)
Observations	83,061	80,569	82,868	83,086	73,669
Panel B: Conflict,Crime and Concern					
	Conflict and Crime		Environmental / Political Concern		
Dep. Vars.	Conflict Count	Being Attacked	Env. Concern 6	Pub. Aff. Int.	Pol. Discussion
	(6)	(7)	(8)	(9)	(10)
Wildfire	− 0.1260*	− 0.0591	− 0.0190	− 0.0111	0.0016
	(0.0748)	(0.0407)	(0.0132)	(0.0705)	(0.0652)
Observations	71,182	82,028	26,206	82,318	82,368
Country-by-Year FE	Yes	Yes	Yes	Yes	Yes
Spatial Cut-off (miles)	20	20	20	20	20
Temporal Cut-off (months)	24	24	24	24	24

Note: This table presents mechanism analysis using different outcome variables with wildfire instrumented by climate conditions in the last 2 years and Land cover combustibility in the 3rd year prior to the interview. All non-binary dependent variables are standardised before estimation. The variable Wildfire is a binary indicator equal to one if a wildfire occurred within 5 miles of the respondent’s village in the two years preceding the survey, and zero if the nearest wildfire occurred between 7 miles and 20 miles during the same period. All specifications include country-by-year fixed effects to account for unobserved heterogeneity and national-level temporal trends. All columns use 2SLS. Coefficients inference is based on two-sided t tests with country-by-year fixed effects and robust standard errors clustered at the subnational-region level and reported in parentheses. No multiple-comparison adjustment was applied. *** *p *< 0.01, ** *p *< 0.05, * *p *< 0.1.

We find that wildfire exposure has negative effects on the likelihood of conflict and physical attack occurrence (Columns (6) and (7) of Panel B). Although seemingly counter-intuitive, most existing evidence links conflict to slower-moving climate pressures that reshape economic opportunities and resource competition^32^. Wildfires are instead episodic and destructive events, resembling earthquakes, which have been shown to reduce conflict and foster cooperation^54^. Our baseline results also align with this logic: wildfire exposure increases participation in community-level activities but does not affect protest involvement.

By contrast, we find no evidence that wildfire exposure increases general public concern (Columns (9) and (10) of Panel B in Table 2). We further examine concerns about environmental risks using Afrobarometer Round 6, the only round whose questionnaire lists all relevant natural hazards. Column (8) shows no significance for individuals who express concern about these environmental issues following wildfire exposure, compared to those farther from wildfire zones. These findings are consistent with the relatively low levels of climate literacy documented in Africa^36^, which may limit the extent to which climate shocks translate into heightened environmental concern.

In conclusion, Table 2 shows that wildfire exposure increases political participation primarily through the wildfire-caused material deprivations and government reactions, rather than abstract public concerns or environmental concerns.

### Persistence of wildfire effects on political participation

We next investigate the persistence of this effect by changing the exposure window from 12 to 84 months prior to the interview by the baseline model specified in Equation (5). Supplementary Fig. 1 shows that the effects on political engagement remain significant and broadly stable across these windows. Across the 12–84-month windows, wildfire proximity increases the intensity of contacting local government by 13.5%–34.1% of a standard deviation (S.D. = 0.890), community-meeting attendance by 28.2%–71.5% (S.D. = 1.310), and collective issue-raising by 25.0%–48.5% (S.D. = 1.294). Its effect on protest remains insignificant. Given the stability of the effects on contacting councillors, attending meetings, and raising collective issues, subsequent analyses use a 24-month exposure window. The persistence of these responses may reflect prolonged material hardship caused by wildfire damage, including deteriorating soil quality and agricultural productivity^55^. Because the wildfire data do not extend before 2000, using an exposure window of more than 36 months before an election would cause substantial sample attrition, we therefore do not estimate long-term effects on voting intentions.

### Moderating role of climate conditions and literacy

Africa exhibits substantial variation in precipitation and climate change literacy. We therefore examine heterogeneity across the Köppen-Geiger typology and levels of climate change literacy^36,56^.

**Fig. 2:**
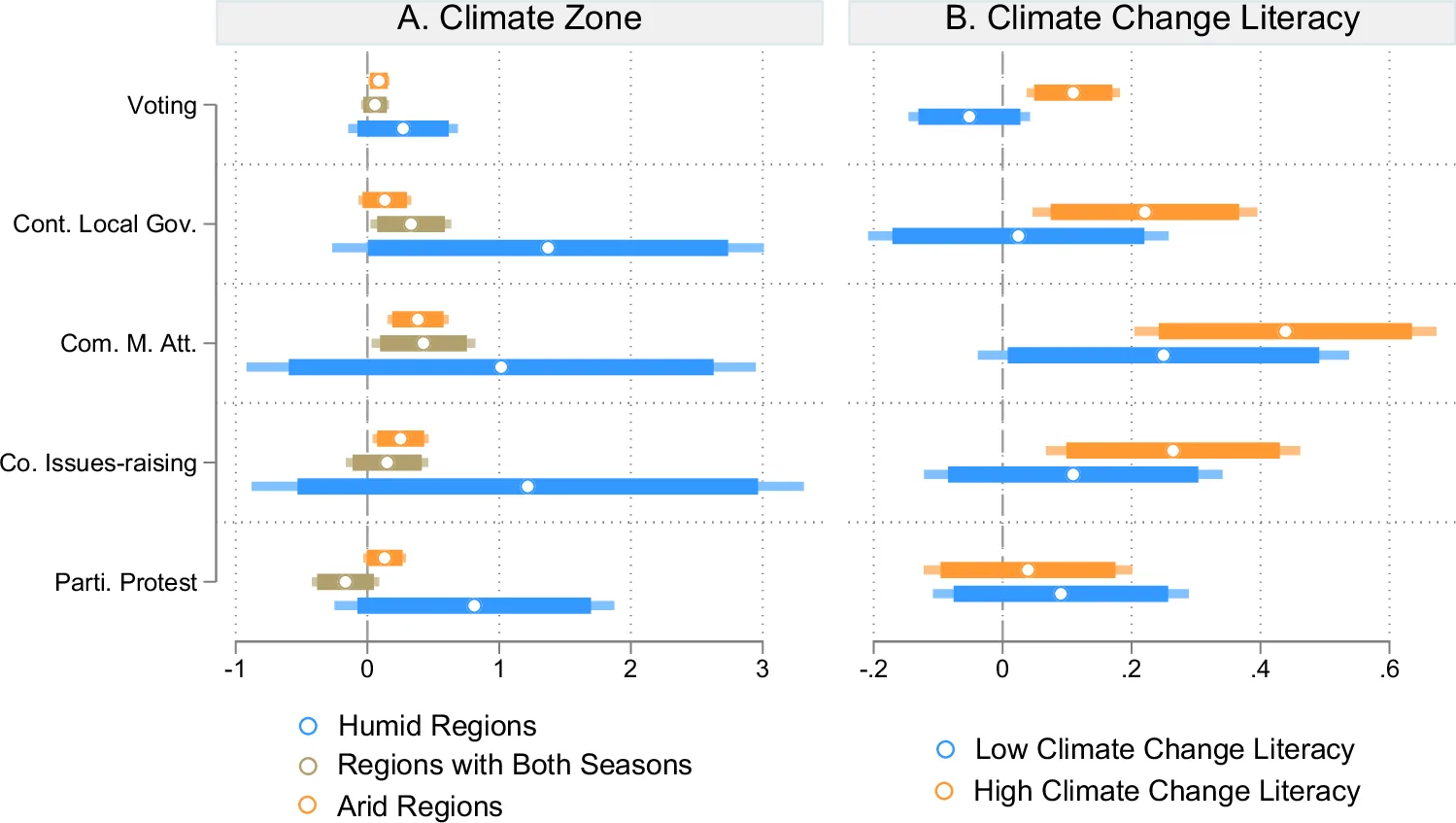
Heterogeneity across climate zones and climate change literacy. **A** Effects by humid, both-season, and arid climate zones. **B** Effects in regions above and below the continental median of climate change literacy (CCL). Open circles are separate 2SLS coefficient estimates for independent respondents; thick and thin horizontal lines show 90% and 95% confidence intervals (CIs), respectively, and the dashed vertical line marks zero. Estimates include country-by-year fixed effects and standard errors clustered by subnational region. CIs and *P*-values use two-sided *t* tests; no multiple-comparison adjustment was applied. Sample sizes and other information are reported in Supplementary Table 27 and Supplementary Table 29.

Humid regions are generally better equipped to prevent and manage large wildfires, potentially muting political responses. In contrast, Residents of arid regions face greater livelihood stress and vulnerability to fire-related damage and may respond more strongly. The results in Fig. 2 align with these expectations. In humid regions, wildfire exposure has only an ambiguous effect on contacting local councillors. In regions with pronounced wet and dry seasons, proximity increases voting and contact with local councillors. In arid regions, it significantly increases voting, community-meeting attendance, and collective issue-raising.

Beyond direct physical exposure to climate impacts, individuals’ climate change literacy (CCL), defined as both awareness of climate change and an understanding of its anthropogenic causes^36,57^, can shape political responses to wildfire shocks. Using regional CCL measures^36^, we divide regions according to whether their average CCL exceeds the continental median and re-estimate the baseline specification in each subsample.

The results in Panel B of Fig. 2 show that individuals in high-CCL regions exhibit significantly greater political participation following wildfire exposure. Those living closer to wildfires show 11.0% (95%CI: 0.038–0.182) higher probability of participating in voting, consistent with findings by Hoffmann et al.^8^. Wildfire exposure also increases contact with local councillors, meeting attendance, and collective issue-raising in high-CCL regions, whereas these effects are absent in low-CCL regions. Greater climate knowledge may therefore direct responses toward institutionalised participation. Protest remains unresponsive to wildfire proximity at both levels of CCL.

### Ethnic power and border proximity in political responses

We next explore how the political status of an ethnic group shapes political participation in response to wildfires. We consider two kinds of political status, with one defined by the GeoEPR-ETH Version 2.0 dataset (GeoEPR-ETH V2) and the other defined by the border proximity^32,58^. We divide the sample into two groups based on the political status variable and re-estimate the baseline specification using Equation (5).

**Fig. 3:**
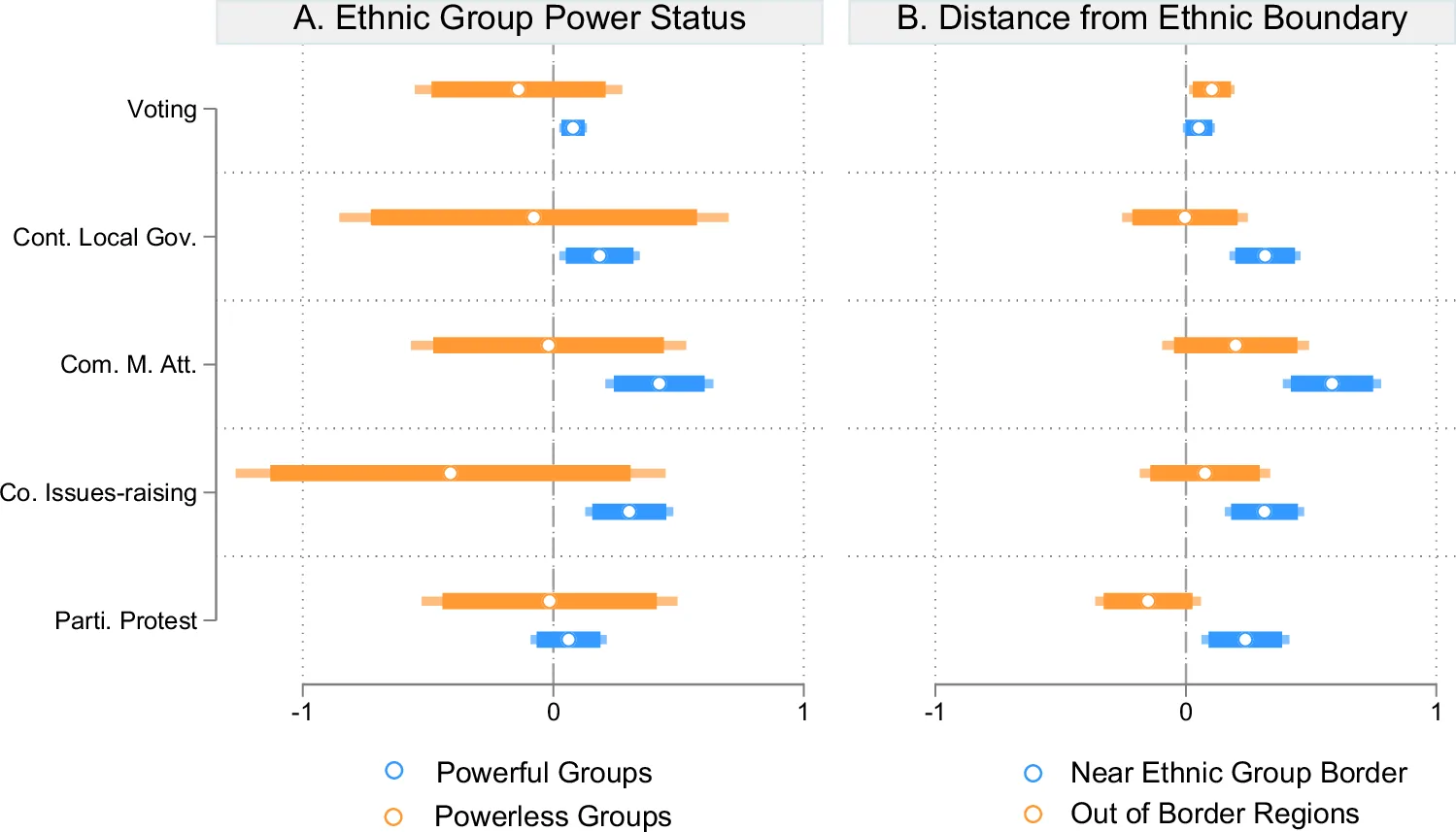
Heterogeneous political responses across ethnic groups. **A** Effects for politically powerful and powerless ethnic groups. **B** Effects within five miles of, and outside, an ethnic-settlement boundary. Open circles are separate 2SLS coefficient estimates for independent respondents; thick and thin horizontal lines show 90% and 95% CIs, respectively, and the dashed vertical line marks zero. Estimates include country-by-year fixed effects and standard errors clustered by subnational region. CIs and *P*-values use two-sided *t* tests; no multiple-comparison adjustment was applied. Sample sizes and other information are reported in Supplementary Table 31 and Supplementary Table 33.

As shown in Panel A of Fig. 3, respondents from dominant ethnic groups exhibit significantly higher levels of political engagement in response to wildfire exposure, compared to respondents further away from the wildfire with the same political status based on the GeoEPR-ETH V2 dataset. Specifically, the probability of voting increases by 7.9% (95% CI: 0.024–0.134) due to proximity to wildfire. More profound influences are observed across the 3 localised activities, including contacting local government officials, attending community meetings, and raising collective issues. Individuals closer to wildfires increase the magnitude of contacting local government councillors by 18.5% (95% CI: 0.024–0.345) from the overall deviation (S.D. = 0.890). A larger effect is observed for attending community meetings and is equal to 42.3% (95% CI: 0.208–0.638) increment from the overall deviation of 1.310, and intensity of participation in community affairs increases by 30.39% (95% CI: 0.127–0.479) of the standard deviation (S.D. = 1.294) among those residing closer to wildfires. Wildfire exposure has only an ambiguous effect on protest participation.

Citizens in less dominant ethnic groups show muted responses when affected by wildfires. Individuals in dominant ethnic groups are generally better resourced and thus more capable of expressing their political appeals. By contrast, members of politically marginalised groups often face systematic repression and are discouraged from participating in political activities due to fear, resignation, or mistrust of existing political institutions^59,60^.

Climate change and related disasters may also reshape inter-group civic activities and conflicts^32^. Respondents residing in potential conflict zones may exhibit heterogeneous political responses. We divided the sample based on the distance from each respondent’s village to the nearest ethnic settlement’s border. Panel B of Fig. 3 shows that individuals within five miles of an ethnic group border exhibit significantly higher levels of political engagement for four activities in response to recent wildfires. Specifically, engagement in local political activities rises sharply, with incremental intensity of 31.6% (95% CI: 0.175–0.458) from the standard deviations (S.D. = 0.890) for contacting local government officials, 58.4% (95% CI: 0.388–0.779) from the standard deviations (S.D. = 1.310) for attending community meetings, and 31.4% (95% CI: 0.156–0.472) standard deviations (S.D. = 1.294) for collective issue-raising. The intensity of protest participation increases by 23.7% (95% CI: 0.062–0.412) from the standard deviations (S.D. = 0.884) among those residing closer to recent wildfires.

Among individuals residing more than five miles from ethnic group borders, wildfire exposure does not increase protest participation; the effect on frequency of contacting local councillors, attending community meetings, and collective issue-raising is also insignificant, but voting turnout rise significantly. Specifically, the intensity (or probability) of voting increases by 10.4% (95% CI: 0.013–0.194) from the overall standard deviations. This discrepancy likely reflects that regions beyond the border generally exhibit stronger social cohesion, where political representation is often dominated by a single ethnic group with stable power dynamics. The need to engage in radical activities, such as protests, is lower because residents may perceive existing local institutions as more aligned with their interests. Those near ethnic boundaries face higher political marginalisation and uncertainty, prompting stronger engagement in local activities when climate shocks like wildfires occur^61^.

### Information and transportation accessibility moderates wildfire impacts

Access to information and transportation infrastructure plays a crucial role in shaping political participation. Existing research shows that improved information and transportation accessibility can strengthen democratic engagement, even in autocracies and emerging democracies^62–64^. To measure respondents’ access to information and transportation, we use three proxies, including the presence of a tarred road in the village, television ownership, and radio ownership. All three variables are self-reported in the Afrobarometer survey.

**Fig. 4:**
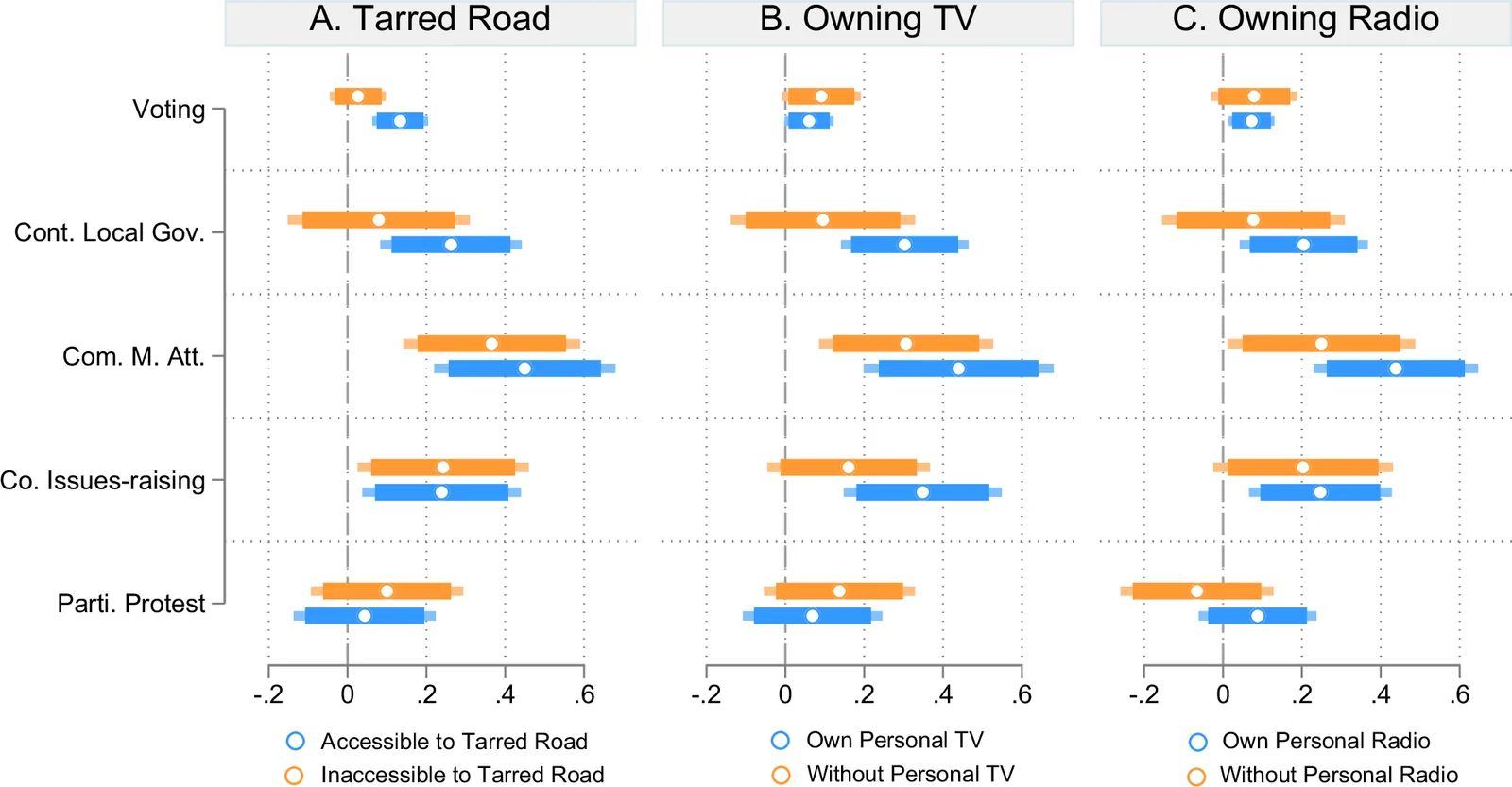
Heterogeneity by information and transportation access. **A** Effects by whether villages have access to a tarred road. **B** Effects by whether respondents own televisions. **C** Effects by whether respondents own a radio. Open circles are separate 2SLS coefficient estimates for independent respondents; thick and thin horizontal lines show 90% and 95% CIs, respectively, and the dashed vertical line marks zero. Estimates include country-by-year fixed effects and standard errors clustered by subnational region. CIs and *P*-values use two-sided *t* tests; no multiple-comparison adjustment was applied. Sample sizes and other information are reported in Supplementary Table 35, Supplementary Table 37 and Supplementary Table 39.

Results are reported in Panel A of Fig. 4, showing that residents with immediate access to a tarred road exhibit higher engagement in voting and contacting local councillors. In contrast, for those without such access, wildfire exposure increases only locally oriented political activities. This asymmetry may be driven by the reduction in commuting costs and better information accessibility provided by road infrastructure^65^.

Then we measure information accessibility by TV ownership and radio ownership in Panels (B, C) of Fig. 4. Respondents with access to TV or radio exhibit higher engagement in contacting local councillors for help following wildfire exposure, whereas no heterogeneous effects are observed across the other four types of activities. Greater media exposure likely increases awareness of wildfire impacts and climate-related cues^53,66^, thereby motivating broader political engagement and demands for reforms.

### Demographic and economic determinants of responsiveness

**Fig. 5:**
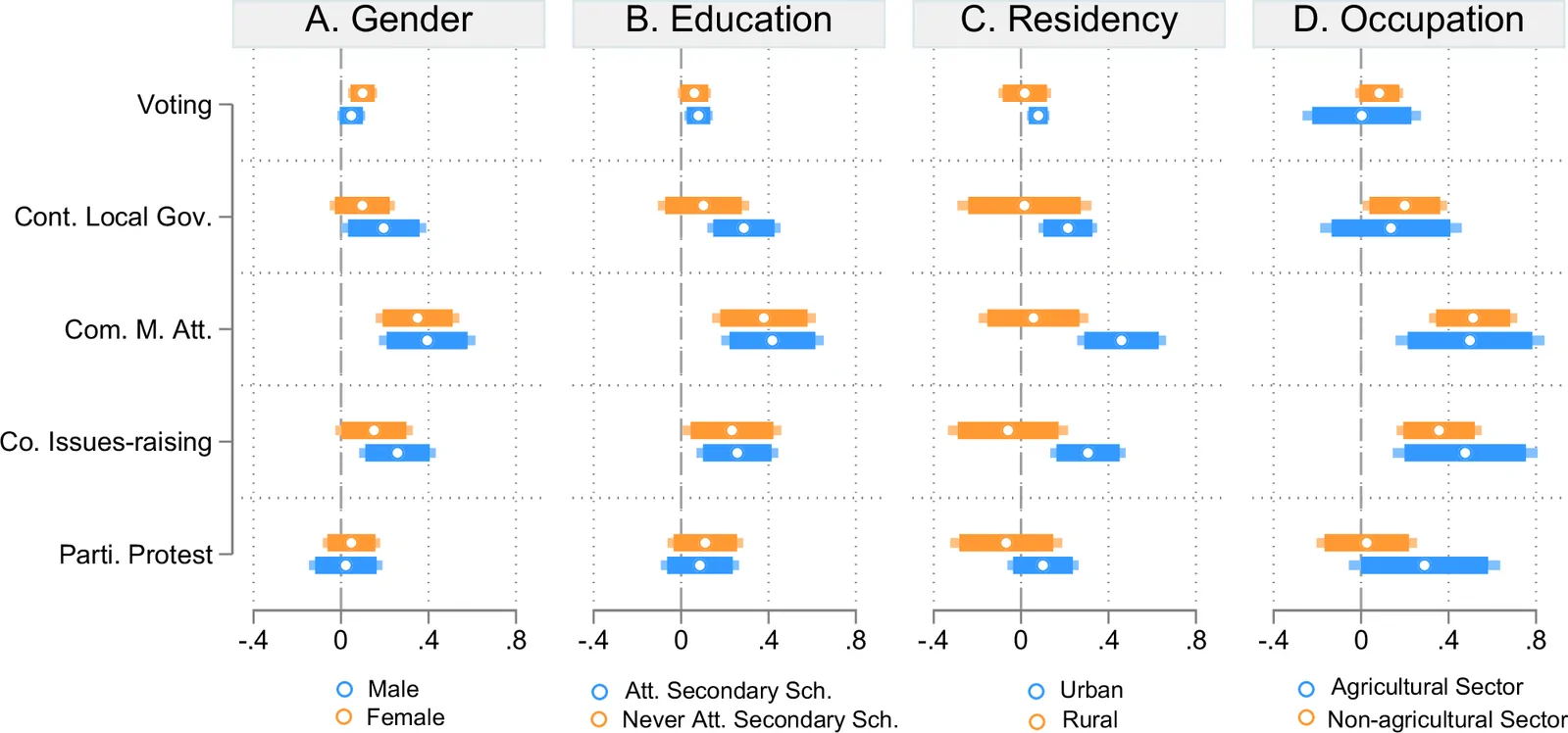
Heterogeneity by demographic and economic characteristics. **A** Effects by gender. **B** Effects by whether respondents attained secondary or higher education. **C** Effects by urban versus rural residency.** D** Effects by agriculture versus non-agriculture occupation. Open circles are separate 2SLS coefficient estimates for independent respondents; thick and thin horizontal lines show 90% and 95% CIs, respectively, and the dashed vertical line marks zero. Estimates include country-by-year fixed effects and standard errors clustered by subnational region. CIs and *P*-values use two-sided *t* tests; no multiple-comparison adjustment was applied. Sample sizes and other information are reported in Supplementary Table 41, Supplementary Table 43, Supplementary Table 45 and Supplementary Table 47.

The Afrobarometer dataset also provides rich demographic information, which helps us to identify the heterogeneous response to wildfires across demographic groups. We focus on gender, education, urban residents, and occupations. Based on Fig. 5, Panel A, the gender-specific patterns in response to wildfire exposure reveal distinct pathways of political engagement, except for voting turnout and contacting local councillors. In fact, we find that the increment in voting turnout for women under closer wildfire exposure is significant, while this effect is ambiguous for men. This result is indeed surprising. Although there is literature suggesting that such disasters provide women with additional political opportunities^67^, the underlying mechanisms remain to be further explored.

The impact of education on political participation is a central question in political science^68^. We group respondents based on their educational attainment, focusing on whether they have secondary schooling experiences. Panel B of Fig. 5 shows that wildfire exposure significantly increases political engagement among individuals with secondary education, which reflects in higher rates of voting and broader civic involvement at the local level. While individuals without secondary education exhibit a statistically significant increase in the frequency (probability) of localised political activities, including attending community meetings and collective issue-raising, they do not participate as much in contacting local councillors and voting as those with secondary experiences following closer wildfire exposure. Our results align with the literature^69,70^ that less-educated individuals tend to engage in pragmatic, community-level actions due to lower political efficacy.

Urban residents exhibit a significantly stronger political response to wildfire exposure compared to rural counterparts (Panel C in Fig. 5), consistent with evidence that wealthier areas express greater negative sentiment under heavy smoke in the U.S.^71^. Differences between respondents in agricultural and non-agricultural sectors are modest (Panel D in Fig. 5). (Notice that only samples in survey Rounds 3 and 6 are included in this analysis since this question is only included in these two survey rounds.) Individuals in both groups attend community meetings more frequently and participate in collective issue-raising when exposed to nearby wildfires. The main divergence appears in institutional activities: only non-agricultural workers show a higher frequency of contacting local councillors, likely reflecting the correlation between non-agricultural occupations and individuals with higher education levels^72^.

For other forms of political activity, we do not detect meaningful differences between the two groups. This pattern contrasts with findings from the Amazon rainforest region, where agricultural producers often benefit economically from large-scale wildfires^22^. The situation in Africa differs substantially from that in South America’s Amazon basin: the scale of slash-and-burn activities is much smaller^73^, and agricultural commercialisation is limited; thus, wildfires typically generate losses rather than economic gains. These structural differences may explain the absence of divergent political responses across livelihood types.

Household wealth is widely recognised as an important source of heterogeneity in response to climate shocks^74^. We focus on housing, a central component of wealth portfolios in rapidly urbanising African economies^75^. We use two housing characteristics as proxies for household wealth: roof combustibility and housing structure. Roofs are classified as combustible or non-combustible, while housing structures are classified as formal or informal. Formal housing refers to permanent dwellings, whereas huts and temporary structures are classified as informal housing. These measures capture housing quality and asset vulnerability rather than constituting a composite wealth index.

**Fig. 6:**
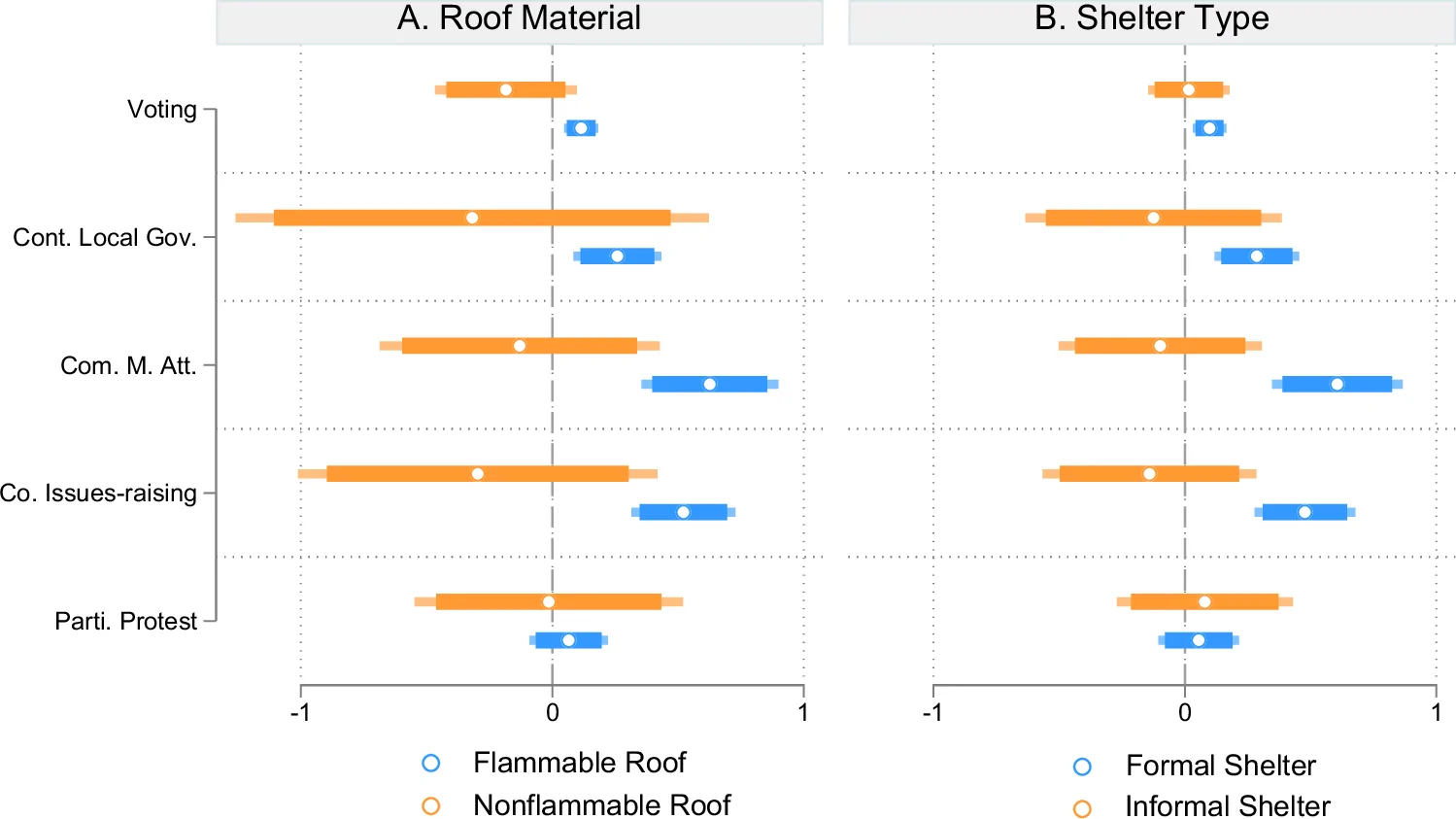
Heterogeneity by roof material and shelter type. **A** Effects by flammable versus non-flammable roof material. **B** Effects by formal versus informal shelter type. Open circles are separate 2SLS coefficient estimates for independent respondents; thick and thin horizontal lines show 90% and 95% CIs, respectively, and the dashed vertical line marks zero. Estimates include country-by-year fixed effects and standard errors clustered by subnational region. CIs and *P* values use two-sided *t* tests; no multiple-comparison adjustment was applied. Sample sizes and other information are reported in Supplementary Table 49 and Supplementary Table 51.

Firstly, we divide the samples based on the combustibility of the roofs of the respondents, and find that individuals living in houses with flammable roofs increase their political participation when exposed to nearby wildfires, whereas no such effect is observed among those with non-flammable roofs (Panel A in Fig. 6).

We then distinguish between formal and informal housing structures. As shown by Panel B of Fig. 6, respondents living in informal dwellings show no detectable changes in political engagement following wildfire exposure, in fact, they even reduce their participation in demonstrations. In contrast, individuals residing in formal housing exhibit increases across nearly all forms of participation (except protest). Specifically, among the latter group, closer wildfire exposure increases the probability of voting by 10.4 per cent (95% CI: 0.051–0.157), and raises the intensity of contacting local government officials, attending community meetings, and engaging in collective issue initiation by 0.306 (95% CI: 0.159–0.454), 0.616 (95% CI: 0.409–0.823), and 0.430 (95% CI: 0.268–0.591) standard deviations respectively. These patterns may appear counterintuitive, yet they are consistent with wealth dynamics in Africa, where formal housing represents a disproportionately large share of household assets. Consequently, wildfires impose greater economic losses on households living in formal structures, generating stronger incentives for political action.

## Discussion

Climate change increases extreme wildfire events, with disproportionate impacts on less-wealthy communities that are most vulnerable to natural disasters^6,76^. Local political participation can influence disaster preparedness, institutional responsiveness, and the distributional fairness of adaptation^9,53^. Using an instrumented LPM and individual-level Afrobarometer data, we show that wildfire exposure persistently increases voting, contact with local officials, community-meeting attendance, and collective issue-raising. The average effect on protest is statistically insignificant, although protest rises among exposed respondents near ethnic boundaries. These conclusions remain stable across exposure windows, estimators, and extensive sensitivity analyses.

Prior research shows that exposure to extreme weather events typically heightens environmental concern and support for climate-related policies. In Europe, exposure increases concern about climate change and electoral support for green parties^8^. In California neighbourhoods affected by large wildfires, support for ballot initiatives expanding government increased by 0.8%, while support for pro-environmental interest groups rose by 2.4%^9^. In contrast, African wildfire exposure does not significantly change concern about weather-related hazards or interest in environmental issues. It instead increases nationwide electoral participation and, more strongly, community-level activities such as contacting officials, attending meetings, and raising collective issues. Climate disasters therefore appear to mobilise African citizens through immediate economic hardship, disruption of daily life, and demand for government assistance rather than through ideological or environmental awakening.^10^.

Our results reflect both limited climate change literacy and the immediate salience of post-wildfire deprivation^36,51^. In developed countries, wildfires and other climate shocks can alter long-term risk preferences and increase support for risk-sharing institutions, social insurance, and government intervention^9,20,21^. African societies bear a disproportionate burden of climatic extremes while often lacking the institutional, financial, and infrastructural capacity required for effective adaptation. Our results show that wildfires exacerbate shortages of basic necessities, especially income and medicine. Material scarcity and demand for responsive government consequently motivate affected communities to seek collective redress and institutional protection against further losses.

We also examine the heterogeneous responses to wildfire exposure. Engagement is stronger in arid regions, where livelihood vulnerability is greater, and in high-CCL regions, where knowledge may help citizens interpret wildfire damage and direct demands toward political institutions. Members of powerful ethnic groups also respond more strongly, consistent with evidence that politically dominant groups possess the resources and institutional access needed for participation, whereas marginalised groups may be repressed or discouraged^59,60^. Respondents living near ethnic boundaries engage more in radical politics when climate shocks like wildfires occur, as residents living in border areas may face higher political marginalisation and uncertainty, prompting political participation after wildfires^61^.

Information and transportation access further shape these responses. Our results align with the literature that wildfire exposure is associated with greater involvement in locally and pro-government-oriented political activities among residents with higher information accessibility across different measurements. Enhanced access to information likely reduces commuting costs, increases awareness of wildfire impacts and climate-related cues^53,66^, thereby motivating broader political engagement.

Responses also vary across demographic and socioeconomic groups. Education promotes broader engagement in contact with local councillors. Urban residents also show stronger responses, underscoring the role of social and economic conditions in shaping civic participation. Heterogeneous analysis based on attributes of real estate generates interesting results. Though it is not surprising to find that respondents with flammable roofs react more strongly, it deserves more attention that the effects are also more noticeable among those with formal houses.

Our occupational findings contribute to debates about the conditions under which climate events increase or reduce political participation. Although many studies find that disasters raise political engagement and support for green policies^77,78^, these effects can weaken or reverse when local economic structures create different incentives^23,24^. In the Amazon, for example, large wildfires can act as de facto land-clearing technologies, causing beneficiaries to oppose rather than support green policies^22^. We cannot directly observe African citizens’ preferences over green policies, but wildfire exposure increases community participation among both agricultural and non-agricultural workers, with only modest differences between them. Unlike commercial producers in the Amazon, African farmers typically practise small-scale household burning and do not benefit economically from landscape-scale fires^73^. Large wildfires, therefore, impose losses on both livelihood groups.

Our research provides a foundation for climate mitigation and adaptation. Post-disaster engagement in Africa is driven primarily by immediate material and public-goods needs, and its effects are much profound for non-electoral community activities than for national elections or protests. This insight underscores the importance of designing domestic climate strategies that directly address citizens’ immediate post-disaster needs, to build trust and ensure public support for long-term adaptation measures. External aid should similarly combine emergency assistance with capacity building, including agricultural resilience, social safety nets, and insurance. Integrating material relief with sustainable adaptation can improve the effectiveness and legitimacy of interventions while promoting more resilient and politically stable communities.

Our findings are consistent with an instrumental, localised form of participation that may reflect clientelistic mobilisation through disaster relief^79,80^. While our data cannot directly test this mechanism, its moral and democratic-theory implications warrant explicit discussion. If parties use disaster relief to influence voters, this practice constitutes a form of corruption: it short-circuits voter evaluations of candidate competence and policy positions, thereby undermining democratic representation. To the extent that programmatic accountability is displaced by patronage, such practices are undemocratic and may contribute to democratic backsliding. Distinguishing genuine accountability pressures from clientelistic mobilisation, therefore, remains an important agenda for future research.

## Methods

### Data

Our primary dataset combines geocoded individual-level data from Afrobarometer Rounds 3 to 6 with 101,292 individuals across 34 African countries between 2005 and 2015, with satellite-derived wildfire records, allowing an assessment of the impact of wildfire exposure on political participation.

#### Political participation data

The political participation data comes from Afrobarometer survey data from Rounds 3 to 6, covering 34 African countries, with a total of 101,292 observations from 2005 to 2015 (see Supplementary Table 1). The dataset provides hyperlocal, time-varying information about the priorities, preferences, experiences, and opinions of African citizens. As Afrobarometer is not a tracking survey, our dataset is constructed as a pooled cross-sectional dataset. To measure respondents’ exposure to wildfire events, we obtained access to the respondent-level geocoded data from Afrobarometer^29^.

We examine five types of political participation, categorised into institutionalised and non-institutionalised forms of political participation. The former includes voting, contacting local government officials, and attending community meetings, while the latter encompasses collective issue-raising and participation in protests. In each survey round, respondents were asked the frequency with which they engaged in these five activities. For all activities except voting, we code responses based on the participation frequency. For example, 0 for “never", 1 for “Only once", 2 for “A few times", and so forth. Responses such as “Don’t know” or “Refused to answer” are coded as missing. For voting, due to inconsistencies in response coding across survey rounds, we standardise the variable by aligning all values with the response structure of Round 5. We assign a value of 1 for respondent reported “voted in the last national election", and 0 otherwise. While respondents were asked to report their frequency of participation in the four non-voting political activities over the past year when surveyed, voting behaviour was assessed based on their participation in the most recent national election (Supplementary Table 2). We provide the descriptive statistics in Supplementary Table 3. We also summarise the subsample, separated by election subsample in Supplementary Table 4 and non-election subsample in Supplementary Table 5.

#### Wildfire exposure index

The wildfire dataset is sourced from the Global Fire Atlas, a dataset that tracks the day-to-day dynamics of individual fires, including the timing and location of ignitions, fire size, duration, daily expansion, fire line length, speed, and direction of spread^28^. These characteristics were derived based on the Global Fire Atlas algorithm and estimated day of burned information at a 500 m resolution from the Moderate Resolution Imaging Spectroradiometer (MODIS) Collection 6 MCD64A1 burned area product over the 2003–2016 study period.

To isolate the effects of large-scale wildfire disasters from those of small-scale slash-and-burn agricultural practices^9,43^, we retain only fires with a burned area exceeding 500 acres, which accounts for approximately 32% of all identified fires. We then calculate the distance from each ignition point of wildfire to the village of every survey respondent. We define a dummy variable,* Wildfire*, which equals one if the nearest wildfire occurred within 5 miles of the respondent’s village during the two years preceding their reported political activity in the Afrobarometer data, and zero if the nearest wildfire occurred 7–20 miles away from the respondent’s village. We exclude the wildfire occurring at a distance of 5–7 miles from a respondent’s location to limit imprecision arising from village geocoding or wildfire coordinates and to reduce potential overlap between treated and control areas that could introduce confounding^44^.

In addition, we examine the impact of the exposure at a broader spatial scale in the sensitive analysis by generating three indicator variables denoted as* I*_0_5_,*I*_5_20_,*I*_20_50_, which equal one if a large wildfire occurred within 0–5, 5–20 or 20–50 miles of the respondent’s village, respectively^9^.

We also account for the impact of both wildfire frequency and burned area across different distance bands in the sensitivity analyses by constructing weighted average measures, as defined in Equations (1) and (2)^81,82^. Specifically, Equation (1) computes a weighted average number of wildfires occurring within each distance band in the same year, using the inverse of the distance band (*d*) as the weight. Similarly, Equation (2) applies the same weighting scheme to burned areas across distance bands:

1 $$\,{{\rm{Weighted}}} \, {{\rm{Number}}}=\mathop{\sum }\limits_{d=1}^{50}\frac{{{\rm{FireCount}}}_{d}}{d}$$

and

2 $${{\rm{Weighted}}} \, {{\rm{Area}}}=\mathop{\sum }\limits_{d=1}^{50}\frac{{{\rm{FireArea}}}_{d}}{d}.$$

To examine the long-term effects of wildfire exposure, we further construct wildfire exposure indicators based on alternative time windows, including the 0–12, 12–24, 24–36, 36–48, 48–60, 60–72, and 72–84 month periods preceding each respondent’s reported political activity in the Afrobarometer data.

#### Other data from afrobarometer

We incorporate additional demographic information and economic conditions from the Afrobarometer data into our analysis. First, in the mechanism analysis, we draw on Afrobarometer data on the frequency of household shortages of essential goods, including water, food, medicine, and cash, over the previous week when surveyed. We also assemble an indicator of the frequency of being physically attacked for the mechanism analysis.

Second, we control for a range of individual-level attributes, including urban residence, gender, age, educational attainment, and employment status in the robustness analysis. Age is log-transformed, and educational attainment is coded using a set of categorical dummy variables. All other covariates are binary indicators.

Third, we included a series of proxy variables for information and infrastructure accessibility in the heterogeneous analysis, including the presence of a tarred road, post office, police station and primary school; ownership of a personal television and ownership of a personal radio. All of these variables are coded as binary indicators.

Finally, we incorporated variables measuring respondents’ political and environmental concerns. To capture political concerns, we relied on two survey items, including the frequency of participation in political discussions during the past week when surveyed and the respondent’s level of interest in public affairs. For environmental concern, if a respondent mentioned any environmental disaster. In the 3rd and 4th rounds of surveys, the options include droughts. In the 5th round of surveys, they include droughts and floods. In the 6th round of surveys, except for droughts, these options were uniformly described as “natural disasters". Thus, we only apply the sample in round 6 to examine the effects of wildfire exposure on environmental concern. In response to any of the three questions “In your opinion, what are the most/second most/third most important problems facing this country that the government should address?", the indicator was coded as 1; otherwise, it was coded as 0. These variables were included in the mechanism analysis.

#### Conflict data

Conflict data are sourced from the Uppsala Conflict Data Programme (UCDP) database^83^. We calculated the number of armed conflicts that occurred within 20 miles of the respondents’ villages in the year of the interview and the two previous years for use in the mechanism analysis.

#### Ethnic group distribution data

The ethnic group distribution and status are sourced from the GeoEPR-ETH Version 2.0 dataset^58^. For each respondent, we calculate the distance from their location to the boundary of their own ethnic group to facilitate the heterogeneity analysis. We categorised ethnic groups’ political status into two tiers. Those designated as “dominant”, “senior partner” or “junior partner” were assigned to the high-political-status category, while those described as “irrelevant”, “discriminated” or “powerless” were classified as low-political-status.

#### Weather, Land-cover and other environmental conditions

We use weather data from ERA5, the fifth-generation ECMWF atmospheric reanalysis of the global climate, including temperature, precipitation, and wind speed, to construct the instrumental variables for wildfire exposure. These variables are measured over two years preceding the interview.

We also use land-cover data to measure flammability. As shown in Supplementary Table 6, bare land, permanent snow and ice, water bodies, and urban and built-up lands are classified as non-combustible, whereas forests, herbaceous areas, croplands, and shrublands are classified as combustible. In robustness checks, we further exclude agricultural and built-up land-cover categories to reduce concerns that the interaction term captures agricultural or urban-related channels rather than wildfire risk. The land-cover data are sourced from MODIS through Google Earth Engine.

### Estimation method

We combined the geocoded individual-level political participation data from Afrobarometer with the wildfire atlas to examine the impact of wildfire exposure on individuals’ political participation. We employ a linear probability model with a series of fixed effects as follows:

3 $${Y}_{i,t}={\alpha }^{o}+{\beta }^{o}Wildfir{e}_{v(i),t-2}+{C}_{c(i)}^{o}\times Yea{r}_{t}^{o}+{\varepsilon }_{i,t}^{o},$$

where *Y*_*it*_ denotes the frequency of respondent *i*’s engagement in political activities during the year preceding the survey conducted in year *t*. We model each intensity outcome as a linear function of (i) the village-level wildfire-exposure index *Wildfire*_*v*(*i*),*t*−2_ in the proceeding two years of the survey date(Because the survey does not record the exact dates the political activities that respondent *i* participated in, we measure wildfire exposure over the two years preceding the interview year *t*), (ii) intercept *α*^*o*^, error term ${\varepsilon }_{i,t}^{o}$ and (iii) country-by-year fixed effects ${C}_{c(i)}^{o}\times Yea{r}_{t}^{o}$. The subscript *v*(*i*) denotes villages of respondents, and *c*(*i*) denotes countries. To address spatial autocorrelation at the sub-national level, the standard errors are clustered at the sub-national level.

However, the OLS estimates from Equation (3) are likely to be endogenous. Wildfire exposure is not randomly assigned across villages, and fire-prone locations may differ systematically in unobserved social, economic, and political characteristics that also affect political participation^22^. In this case, OLS would confound the effect of wildfire exposure with these underlying local conditions. Existing literature addresses this issue by exploiting natural variation in wildfire risk or ignition conditions. For example, Pianta and Rettl^22^ instrument wildfire exposure with short-term weather fluctuations, noting that longer-term weather conditions may affect voting behaviour through channels other than fires. Umetani^84^ instead uses lightning strikes as an instrument for wildfire exposure. Related work also shows that drought is an important predictor of large-scale fire activity^85^. However, this strategy is less feasible in our setting because comparable lightning data are not consistently available across our Africa-wide sample and study period.

Because our outcomes measure political participation over the year preceding the survey, it is not appropriate to leverage short-term (e.g., one week before specific events) fluctuations of weather conditions as instrumental variables directly, as Pianta and Rettl^22^, while using weather or drought directly as instruments is less suitable in our setting. These climatic conditions may affect political behaviour through channels other than wildfire, including agricultural production, household income, and local economic stress^86^, making the exclusion restriction less plausible when they are used alone as instruments. We therefore construct an interaction-based instrument that is better aligned with our cumulative wildfire exposure measure. Specifically, we interact meteorological conditions measured over the two years preceding the survey (*Tem*_*v*(*i*),*t*−2_, *Prep*_*v*(*i*),*t*−2_, and *Windspeed*_*v*(*i*),*t*−2_) with a predetermined measure of land-cover flammability observed one year earlier (*I*(*LC*)_*v*(*i*),*t*−3_). These factors jointly shape the likelihood and intensity of wildfires^22,45^. This timing is intentional: weather is measured over the wildfire-exposure window to capture the conditions that generate fires, whereas land-cover flammability is measured earlier because it is a predetermined determinant of wildfire risk that may itself be altered by recent fires. In the estimation, we control separately for meteorological conditions and land-cover characteristics. These controls and a series of robustness checks substantially reduce concerns that the instruments capture non-wildfire channels related to weather or land cover. The remaining identifying variation is therefore tied to the interaction-based component of weather variation in more combustible areas, although the exclusion restriction ultimately remains an identifying assumption^46,47,87^.

Accordingly, the wildfire risk instrument vector is:

4 $${{{\bf{WildfireRisk}}}}_{v(i),t-2}=\left[\begin{array}{c}Pre{p}_{v(i),t-2}\cdot I{(LC)}_{v(i),t-3}\\ Te{m}_{v(i),t-2}\cdot I{(LC)}_{v(i),t-3}\\ Windspee{d}_{v(i),t-2}\end{array}\right]$$

And the full model with the first stage is derived as:

5 $$\left\{\begin{array}{l}{Y}_{i,t}=\alpha+{\beta }_{1}\cdot \widehat{Wildfir{e}_{v(i),t-2}}+{{{\mathbf{\beta }}}}_{{{\bf{2}}}}\cdot {{{\bf{CoV}}}}_{{{\bf{v(i),t}}}}+{C}_{c(i)}\times Yea{r}_{t}+{\varepsilon }_{i,t},\hfill\\ Wildfir{e}_{v(i),t-2}=\eta+{{{\mathbf{\gamma }}}}_{{{\bf{1}}}}\cdot {{{\bf{WildfireRisk}}}}_{v(i),t-2}+{{{\mathbf{\gamma }}}}_{{{\bf{2}}}}\cdot {{{\bf{CoV}}}}_{{{\bf{v(i),t}}}}+{C}_{c(i)}\times Yea{r}_{t}+{\epsilon }_{i,t}\end{array}\right.$$

where **WildfireRisk**_*v*(*i*),*t*−2_ is the instrumental variable of wildfire, calculated by Equation (4), and **CoV**_**v(i),t**_ is the temperature, precipitation, and land-cover characteristics at year *t* when surveyed. By incorporating country-by-year fixed effects *C*_*c*(*i*)_ × *Year*_*t*_, we account for unobserved county-specific time shocks such as war, structural economic change, and large-scale social unrest that could otherwise bias our estimates.

We carried out a series of robustness checks to assess potential threats to the exclusion restriction. As shown in Supplementary Table 6, the land-cover categories include not only natural environments, such as ice and snow, sandy areas, water bodies, forests, and grasslands, but also built-up areas and agricultural zones. One possible concern is that some land-cover categories correspond to agricultural or built-up environments, so the interaction term could partly reflect channels other than wildfire risk. For example, agricultural land may be associated with local production conditions, while built-up land may be correlated with settlement intensity and related social or economic activity. To assess whether our results are driven by these alternative channels, we conduct three robustness checks by excluding observations in agricultural areas, built-up areas, and both agricultural and built-up areas. As reported in Supplementary Tables 12–14, the results remain consistent with the baseline estimates. A related concern is that temperature and precipitation may affect political participation through nonlinear non-wildfire channels, such as agricultural production, livelihood stress, or other weather-sensitive local conditions. To probe this possibility, we augment the baseline specification with quadratic and cubic terms for temperature and precipitation. The results, reported in Supplementary Table 15, remain similar to the baseline estimates. These cheques reduce the concern that the estimates are driven by simple nonlinear weather effects, but they cannot rule out all unobserved or more complex nonlinear pathways.

We further conduct a falsification test to assess whether the instruments are systematically associated with potential mechanism outcomes after controlling for wildfire exposure and the full set of baseline covariates. As reported in Supplementary Table 20, the estimated coefficients on the individual instruments are generally small and statistically weak across all specifications. Moreover, the joint Wald F-statistics testing the null hypothesis that the coefficients on the three instruments are jointly equal to zero are uniformly low. After applying Holm’s step-down adjustment to account for multiple hypothesis testing across outcome groups, none of these joint tests remains statistically significant. We also perform an over-identification test (Table 1), which does not reject the null hypothesis.

Beyond these exclusion-restriction cheques, we also assess the robustness of the estimates to additional covariates and alternative model specifications. We further control for respondent characteristics, including urban residency, gender, age, and access to personal information channels (Supplementary Table 16, 17). We also adopt alternative specifications, including ordered probit and ordered instrumented probit models (Supplementary Table 21). Moreover, to facilitate interpretation, we standardised all non-binary dependent variables before estimation, so each estimated coefficient $\hat{\beta }$ represents the treatment-control difference in standard deviations of the outcome. This facilitates comparisons across different model specifications.

Taken together, these cheques show that the estimates are stable across a range of specifications and do not reveal systematic inconsistencies. However, because they cannot exhaust all possible pathways, we interpret them as supporting the plausibility of the exclusion restriction rather than proving it.

In the heterogeneity analysis, we categorise the dataset into subsamples according to the relevant variables and re-estimate the baseline specification using Equation (5). For the mechanism analysis, we fit a model in Equation (5) but employing potential mediation variables as dependent variables.

### Reporting summary

Further information on research design is available in the Nature Portfolio Reporting Summary linked to this article.

## Supplementary information

Supplementary Information

Reporting Summary

Transparent Peer Review file

## Data Availability

The Global Fire Atlas data are available at https://essd.copernicus.org/articles/11/529/2019/. The Afrobarometer geocoded data (Rounds 3–6) are available at https://www.afrobarometer.org/data. The ERA5 reanalysis data are available from the Copernicus Climate Change Service (https://cds.climate.copernicus.eu/datasets/reanalysis-era5-single-levels?tab=overview). The MODIS fire product (MOD14/MYD14) is available from NASA LP DAAC. The UCDP conflict data are available at https://ucdp.uu.se/. The GeoEPR-ETH v2 data are available at https://icr.ethz.ch/data/epr/geoepr/v2.0/. The climate-literacy dataset can be sourced from the study of Simpson et al.^36^ The individual-level analysis dataset and replication code are available in a Code Ocean capsule at https://codeocean.com/capsule/6875400/treeand will be published alongside the article with a permanent DOI. The raw geographic coordinates used in this study are subject to third-party restrictions and were obtained under license; therefore, they are not publicly available.

## Code availability

All data obtained for this study was eventually saved as “.dta" format files for analysis and edited in Stata (Version 18 MP, open source packages: ftools, require, reghdfe, ivreghdfe, coefplot, gtools, ranktest and ivreg2). During the data processing, some data was stored and edited in ArcGIS pro 3.1.5 (arcpy) through Python (Version 3.9, open source packages used: numpy, pandas and geopandas) or UI. All data was analysed in Stata (Version 18 MP, open source packages used: ftools, require, reghdfe, ivreghdfe, coefplot, gtools, ranktest and ivreg2). The replication code for all analyses and figure generation is available in the same Code Ocean capsule at https://codeocean.com/capsule/6875400/treeand will be published alongside the article with a permanent DOI. The capsule contains all the necessary code, software environment, and input data to reproduce the main findings.

## References

[1] IPCC.* Climate Change 2022: Impacts, Adaptation, and Vulnerability.* (Cambridge University Press, 2022).

[2] Cunningham, C. X., Williamson, G. J. & Bowman, D. M. Increasing frequency and intensity of the most extreme wildfires on Earth. *Nat. Ecol. Evol.* **8**, 1420–1425 (2024). 10.1038/s41559-024-02452-2 38914710

[3] Giglio, L., Randerson, J. T. & van der Werf, G. R. Analysis of daily, monthly, and annual burned area using the fourth-generation global fire emissions database (GFED4). *J. Geophys. Res. Biogeosci.* **118**, 317–328 (2013).

[4] Andela, N. & van der Werf, G. R. Recent trends in African fires driven by cropland expansion and El Niño to La Niña transition. *Nat. Clim. Change* **4**, 791–795 (2014).

[5] Yu, Y. et al. Quantifying the drivers and predictability of seasonal changes in African fire [Journal Article]. *Nat. Commun.* **11**, 2893 (2020). 10.1038/s41467-020-16692-w PMC7283213 32518232

[6] Burton, C. et al. Global burned area increasingly explained by climate change. *Nat. Clim. Change* **14**, 1186–1192 (2024).

[7] Castells-Quintana, D., Lopez-Uribe, M. P. & McDermott, T. K. J. Population displacement and urban conflict: Global evidence from more than 3300 flood events [Journal Article]. *J. Dev. Econ.* **158**, 102922 (2022).

[8] Hoffmann, R., Muttarak, R., Peisker, J. & Stanig, P. Climate change experiences raise environmental concerns and promote green voting. *Nat. Clim. Change* **12**, 148–155 (2022).

[9] Coury, M. Climate risk and preferences over the size of government: evidence from California wildfires. * Rev. Econ. Stat.* **107**, 697–710 (2025).

[10] Arias, S. B. & Blair, C. W. In the eye of the storm: hurricanes, climate migration, and climate attitudes. *Am. Political Sci. Rev.* **118**, 1593–1613 (2024).

[11] Baerlocher, D., Caldas, R., Cavalcanti, F. & Schneider, R. Natural disasters and voting behavior under authoritarian regimes: Evidence from the Brazilian shrimp vote [Journal Article]. *J. Econ. Behav. Organ.* **234**, 106998 (2025).

[12] Bratton, M. & Mattes, R. Support for democracy in Africa: intrinsic or instrumental? *Br. J. Political Sci.* **31**, 447–474 (2001).

[13] Mattes, R. & Bratton, M. Learning about democracy in Africa: awareness, performance, and experience. *Am. J. Political Sci.* **51**, 192–217 (2007).

[14] Wilfahrt, M. Citizen response to local service provision: Emerging democratic accountability in decentralized West Africa? *Elect. Stud.* **79**, 102498 (2022).

[15] Gaventa, J. & Barrett, G. Mapping the outcomes of citizen engagement. *World Dev.* **40**, 2399–2410 (2012).

[16] Ribot, J.* Democratic Decentralization of Natural Resources: Institutionalizing Popular Participation.* (2002).

[17] Bénit-Gbaffou, C. Party politics, civil society and local democracy–Reflections from Johannesburg. *Geoforum* **43**, 178–189 (2012).

[18] Smoke, P. Decentralisation in Africa: goals, dimensions, myths and challenges. *Public Adm. Dev. Int. J. Manag. Res. Pract.* **23**, 7–16 (2003).

[19] Crook, R.C., Manor, J. Democracy and Decentralisation in South Asia and West Africa: Participation, Accountability and Performance. (Cambridge University Press, 1998).

[20] Hanaoka, C., Shigeoka, H. & Watanabe, Y. Do Risk Preferences Change? Evidence from the Great East Japan Earthquake. *Am. Econo. J. Appl. Econ.* **10**, 298–330 (2018).

[21] Gärtner, M., Mollerstrom, J. & Seim, D. Individual risk preferences and the demand for redistribution. *J. Public Econ.* **153**, 49–55 (2017).

[22] Pianta, S. & Rettl, P.* Global Harms, Local Profits: How the Uneven Costs of Natural Disasters Affect Support for Green Political Platforms*. (Harvard Business School, 2023).

[23] Hilbig, H. & Riaz, S. Natural disasters and green party support. * J. Politics* **86**, 241–256 (2024).

[24] Cremaschi, S. & Stanig, P. Voting and climate change: how an extreme weather event increased support for a radical-right incumbent in Italy. * J. Politics* **87**, 1617–1622 (2025).

[25] Zuern, E.* The Politics of Necessity: Community Organizing and Democracy in South Africa.* (University of Wisconsin Press, 2011).

[26] Eifert, B., Miguel, E. & Posner, D. N. Political competition and ethnic identification in Africa. *Am. J. political Sci.* **54**, 494–510 (2010).

[27] Kuenzi, M. & Lambright, G. Party system institutionalization in 30 African countries. *Part. Politics* **7**, 437–468 (2001).

[28] Andela, N., Morton, D. C., Giglio, L. & Randerson, J. T.* Global Fire Atlas with Characteristics of Individual Fires, 2003-2016.* (219).

[29] BenYishay, A. et al. Geocoding afrobarometer rounds 1-6: Methodology & data quality. *Williamsburg, VA: AidData at William & Mary*. (2017)

[30] Gilli, M., Calcaterra, M., Emmerling, J. & Granella, F. Climate change impacts on the within-country income distributions. *J. Environ. Econ. Manag.* **127**, 103012 (2024).

[31] Henderson, J. V., Storeygard, A. & Deichmann, U. Has climate change driven urbanization in Africa? *J. Dev. Econ.* **124**, 60–82 (2017). 10.1016/j.jdeveco.2016.09.001 PMC5404830 28458445

[32] McGuirk, E. F. & Nunn, N. Transhumant pastoralism, climate change, and conflict in Africa. *Rev. Econ. Stud.* **92**, 404–441 (2024).

[33] Koren, O. & Bukari, K. N. (Re)Emerging disease and conflict risk in Africa, 1997-2019 [Journal Article]. *Nat. Hum. Behav.* **8**, 1506–1513 (2024). 10.1038/s41562-024-01929-1 39009715

[34] Zhu, Y. et al. Burden of infant mortality associated with flood in 37 African countries [Journal Article]. *Nat. Commun.* **15**, 10171 (2024). 10.1038/s41467-024-54561-y PMC11585599 39580499

[35] Mackay, R., Mavisakalyan, A. & Tarverdi, Y. Trust a few: Natural disasters and the disruption of trust in Africa [Journal Article]. *J. Behav. Exp. Econ.* **113**, 102288 (2024).

[36] Simpson, N. P. et al. Climate Change Literacy in Africa. *Nat. Clim. Change* **11**, 937–944 (2021).

[37] Hazlett, C. & Mildenberger, M. Wildfire exposure increases pro-environment voting within democratic but not republican areas. *Am. Political Sci. Rev.* **114**, 1359–1365 (2020).

[38] Aarøe, L. et al. Genetic predictors of educational attainment and intelligence test performance predict voter turnout. *Nat. Hum. Behav.* **5**, 281–291 (2021). 10.1038/s41562-020-00952-2 33168953

[39] Donati, D. Mobile Internet access and political outcomes: Evidence from South Africa. *J. Dev. Econ.* **162**, 103073 (2023).

[40] Enikolopov, R., Makarin, A. & Petrova, M. Social media and protest participation: Evidence from Russia. *Econometrica* **88**, 1479–1514 (2020).

[41] Robinson, A. L. & Gottlieb, J. How to close the gender gap in political participation: Lessons from matrilineal societies in Africa. *Br. J. Political Sci.* **51**, 68–92 (2021).

[42] Fujiwara, T., Meng, K. & Vogl, T. Habit formation in voting: Evidence from rainy elections. *Am. Econ. J. Appl. Econ.* **8**, 160–188 (2016).

[43] Bougma, P. T. C., Bondé, L., Yaro, V. S. O., Nikiéma, B. & Ouédraogo, O. Socio-ecological factors contributing to the use of slash-and-burn as cropland preparation practice in Burkina Faso. *Sci. Afr.* **28**, e02736 (2025).

[44] Druckenmiller, H., Liao, Y., Pesek, S., Walls, M. & Zhang, S. Removing development incentives in risky areas promotes climate adaptation. *Nat. Clim. Change* **14**, 936–942 (2024).

[45] Jolly, W. M. et al. Climate-induced variations in global wildfire danger from 1979 to 2013. *Nat. Commun.* **6**, 7537 (2015). 10.1038/ncomms8537 PMC4803474 26172867

[46] Borgschulte, M., Molitor, D. & Zou, E. Y. Air pollution and the labor market: Evidence from wildfire smoke. *Rev. Econ. Stat.* **106**, 1558–1575 (2024).

[47] Meier, S., Elliott, R. & Strobl, E. The regional economic impact of wildfires: Evidence from Southern Europe. *J. Environ. Econ. Manag.* **118**, 102787 (2023).

[48] Holm, S. A Simple Sequentially Rejective Multiple Test Procedure. *Scand. J. Stat.* **6**, 65–70 (1979).

[49] Modaresi Rad, A. et al. Human and infrastructure exposure to large wildfires in the United States. *Nat. Sustain.* **6**, 1343–1351 (2023).

[50] Baarsch, F. et al. The impact of climate change on incomes and convergence in Africa. *World Dev.* **126**, 104699 (2020).

[51] Harari, M. & Ferrara, E. L. Conflict, climate, and cells: A disaggregated analysis. *Rev. Econ. Stat.* **100**, 594–608 (2018).

[52] Bateson, R. Crime victimization and political participation. *Am. Political Sci. Rev.* **106**, 570–587 (2012).

[53] Fabel, M. et al. The relationship between the youth-led Fridays for Future climate movement and voting, politician and media behaviour in Germany. *Nat. Hum. Behav.* **9**, 481–495 (2025). 10.1038/s41562-024-02075-4 39715872

[54] De Juan, A., Pierskalla, J. & Schwarz, E. Natural disasters, aid distribution, and social conflict–Micro-level evidence from the 2015 earthquake in Nepal. *World Dev.* **126**, 104715 (2020).

[55] Pellegrini, A. F. A. et al. Fire frequency drives decadal changes in soil carbon and nitrogen and ecosystem productivity. *Nature* **553**, 194–198 (2018). 10.1038/nature24668 29227988

[56] Beck, H. E., Zimmermann, N. E., McVicar, T. R., Vergopolan, N., Berg, A. & Wood, E. F. Present and future Köppen-Geiger climate classification maps at 1-km resolution. *Sci. Data* **5**, 180214 (2018). 10.1038/sdata.2018.214 PMC6207062 30375988

[57] Helbling, M., Auer, D., Meierrieks, D., Mistry, M. & Schaub, M. Climate change literacy and migration potential: micro-level evidence from Africa. *Clim. Change* **169**, 9 (2021).

[58] Wucherpfennig, J., Weidmann, N. B., Girardin, L., Cederman, L. E. & Wimmer, A. Politically relevant ethnic groups across space and time: Introducing the GeoEPR dataset. *Confl. Manag. Peace Sci.* **28**, 423–437 (2011).

[59] Bratton, M. & Masunungure, E. Zimbabwe’s long agony. *J. Democr.* **19**, 41–55 (2008).

[60] Ackermann, K., Churchill, S. A. & Smyth, R. Estimating the relationship between ethnic inequality, conflict and voter turnout in Africa using geocoded data. *World Dev.* **180**, 106644 (2024).

[61] Michalopoulos, S. & Papaioannou, E. The long-run effects of the scramble for Africa. *Am. Econ. Rev.* **106**, 1802–1848 (2016).

[62] Carvalho, A. Media (ted) discourses and climate change: a focus on political subjectivity and (dis) engagement. *Wiley Interdiscip. Rev. Clim. Change* **1**, 172–179 (2010).

[63] Lorenz-Spreen, P., Oswald, L., Lewandowsky, S. & Hertwig, R. A systematic review of worldwide causal and correlational evidence on digital media and democracy. *Nat. Hum. Behav.* **7**, 74–101 (2023). 10.1038/s41562-022-01460-1 PMC9883171 36344657

[64] Cantoni, E. A precinct too far: Turnout and voting costs. *Am. Econ. J. Appl. Econ.* **12**, 61–85 (2020).

[65] Glaeser, E. L. & Ponzetto, G. A. The political economy of transportation investment. *Econ. Transp.* **13**, 4–26 (2018).

[66] Wonneberger, A., Meijers, M. H. & Schuck, A. R. Shifting public engagement: How media coverage of climate change conferences affects climate change audience segments. *Public Underst. Sci.* **29**, 176–193 (2020). 10.1177/0963662519886474 PMC7323770 31709905

[67] Brulé, R. Climate shocks and gendered political transformation: How crises alter women’s political representation. *Politics Gend.* **19**, 928–934 (2023).

[68] Willeck, C. & Mendelberg, T. Education and political participation. *Annu. Rev. Political Sci.* **25**, 89–110 (2022).

[69] Coffe, H. & Bolzendahl, C. Gender gaps in political participation across sub-Saharan African nations. *Soc. Indic. Res.* **102**, 245–264 (2011). 10.1007/s11205-010-9676-6 PMC3065638 21516140

[70] Larreguy, H. & Marshall, J. The effect of education on civic and political engagement in nonconsolidated democracies: Evidence from Nigeria. *Rev. Econ. Stat.* **99**, 387–401 (2017).

[71] Burke, M. et al. Exposures and behavioural responses to wildfire smoke. *Nat. Hum. Behav.* **6**, 1351–1361 (2022). 10.1038/s41562-022-01396-6 35798884

[72] Geys, B. & Sørensen, R. J. Public sector employment and voter turnout. *Am. Political Sci. Rev.* **116**, 367–373 (2022).

[73] Bezerra, J. S., Arroyo-Rodríguez, V., Dupuy-Rada, J. M., Leal, I. R. & Tabarelli, M. Negative impact of slash-and-burn agriculture on the seed rain in a tropical dry forest. *For. Ecol. Manag.* **531**, 120821 (2023).

[74] Chapagain, P. S. et al. Studies on adaptive capacity to climate change: a synthesis of changing concepts, dimensions, and indicators. *Humanit. Soc. Sci. Commun.* **12**, 1–10 (2025).

[75] Michaels, G. et al. Planning ahead for better neighborhoods: Long-run evidence from Tanzania. *J. Political Econ.* **129**, 2112–2156 (2021).

[76] Reining, S., Wussow, M., Zanocco, C. & Neumann, D. Roof renewal disparities widen the equity gap in residential wildfire protection [Journal Article]. *Nat. Commun.* **16**, 463 (2025). 10.1038/s41467-024-55705-w PMC11707273 39774145

[77] Garside, S. & Zhai, H. *Res. Politics* **9**, 20531680221141523 (2022).

[78] Kronborg, A., Hedegaard, F., Klindt, I. & Vandeweerdt, C. Do green parties in government benefit from natural catastrophes? How wildfires are linked to voting. *Elect. Stud.* **88**, 102749 (2024).

[79] Hicken, A. Clientelism. *Annu. Rev. Political Sci.* **14**, 289–310 (2011).

[80] Lemarchand, R. Political clientelism and ethnicity in tropical Africa:* Competing solidarities in nation-building. *Am. Political Sci. Rev.* **66**, 68–90 (1972).

[81] He, G., Liu, T. & Zhou, M. Straw burning, PM2.5, and death: Evidence from China [Journal Article]. *J. Dev. Econ.* **145**, 102468 (2020).

[82] Du, R., Mino, A., Wang, J. & Zheng, S. Transboundary vegetation fire smoke and expressed sentiment: Evidence from Twitter [Journal Article]. *J. Environ. Econ. Manag.* **124**, 102928 (2024).

[83] Sundberg, R. & Melander, E. Introducing the UCDP georeferenced event dataset. *J. Peace Res.* **50**, 523–532 (2013).

[84] Umetani, H.* Wildfires, Pollution, and Pregnancy Outcomes: Evidence from Lightning Strikes*. (2024).

[85] Aragão, L. E. et al. 21st Century drought-related fires counteract the decline of Amazon deforestation carbon emissions. *Nat. Commun.* **9**, 536 (2018). 10.1038/s41467-017-02771-y PMC5811599 29440640

[86] Mellon, J. Rain, rain, go away: 194 potential exclusion-restriction violations for studies using weather as an instrumental variable. *Am. J. Political Sci.* **69**, 881–898 (2025).

[87] Gasper, J. T. & Reeves, A. Make it rain? Retrospection and the attentive electorate in the context of natural disasters. *Am. J. Political Sci.* **55**, 340–355 (2011).

