# Supplementary material for: Wildfire exposure and political participation in Africa
Source: Nat Commun. 2026 Sep 12;17:10760. doi: 10.1038/s41467-026-77404-4 (PMC13662189; doi:10.1038/s41467-026-77404-4)
Supplement: Supplementary file 1 [file 41467_2026_77404_MOESM1_ESM.pdf]

# Supplementary Information for “Wildfire Exposure and Political Participation in Africa”

## Contents

1 Summary Statistics 2

2 Sensitivity Analysis 8

3 Mechanism Analysis 23

4 Heterogeneity Analysis 24

5 Supplementary Notes 55

# 1 Summary Statistics

**Supplementary Table 1** Survey frequency by country

| Country | Frequency | Year Started | Round |
|---|---|---|---|
| Algeria | 1,230 | 2013 | 5, 6 |
| Benin | 4,469 | 2005 | 3, 4, 5, 6 |
| Botswana | 3,340 | 2005 | 3, 4, 5, 6 |
| Burkina Faso | 2,791 | 2008 | 4, 5, 6 |
| Burundi | 1,079 | 2012 | 5, 6 |
| Cameroon | 1,721 | 2013 | 5 , 6 |
| Cote D'Ivoire | 1,926 | 2013 | 5, 6 |
| Egypt | 427 | 2013 | 5, 6 |
| Gabon | 199 | 2015 | 6 |
| Ghana | 5,031 | 2005 | 3, 4, 5, 6 |
| Guinea | 2,197 | 2013 | 5, 6 |
| Kenya | 1,656 | 2005 | 3, 4, 5, 6 |
| Lesotho | 2,500 | 2005 | 3, 4, 5, 6 |
| Liberia | 2,145 | 2008 | 5, 6 |
| Madagascar | 3,166 | 2005 | 3, 4, 5, 6 |
| Malawi | 5,579 | 2005 | 3, 4, 5, 6 |
| Mali | 4,103 | 2005 | 3, 4, 5, 6 |
| Morocco | 510 | 2013 | 5, 6 |
| Mozambique | 6,267 | 2005 | 3, 4, 5, 6 |
| Namibia | 2,818 | 2006 | 3, 4, 5, 6 |
| Niger | 256 | 2013 | 5, 6 |
| Nigeria | 7,282 | 2005 | 3, 4, 5, 6 |
| Senegal | 2,581 | 2005 | 3, 4, 5, 6 |
| Sierra Leone | 2,085 | 2012 | 5, 6 |
| South Africa | 8,499 | 2006 | 3, 4, 5, 6 |
| Sudan | 641 | 2013 | 5, 6 |
| Swaziland | 1,868 | 2013 | 5, 6 |
| Tanzania | 4,442 | 2005 | 3, 4, 5, 6 |
| Togo | 1,846 | 2012 | 5, 6 |
| Tunisia | 872 | 2013 | 5, 6 |
| Uganda | 7,996 | 2005 | 3, 4, 5, 6 |
| Zambia | 4,086 | 2005 | 3, 4, 5, 6 |
| Zimbabwe | 5,684 | 2005 | 3, 4, 5, 6 |

*Note*: The data are drawn from Afrobarometer Rounds 3 to 6 (2005–2015), accessed at https://www.afrobarometer.org/geocoded-data/.

**Supplementary Table 2** Survey questions for political participation

| Vars. | Question | Answers |
|---|---|---|
| Voting | With regard to the most recent, 20xx General elections, which statement is true for you? | 1 = You voted in the elections, 0 = You were not registered to vote; or You decided not to vote; or You could not find the polling station; or You did not have time to vote; or You did not vote because your name not on the list; or Didnot vote for some other reason |
| Contacting local govt. | During the past year, how often have you contacted any of the following persons for help to solve a problem or to give them your views: A Local Government Councillor? | 0=Never, 1=Only once, 2=A few times, 3=Often, Missing Data = Don't Know, or refused to answer |
| Attending com. meeting | Here is a list of actions that people sometimes take as citizens. For each of these, please tell me whether you, personally, have done any of these things during the past year. If not, would you do this if you had the chance: Attended a community meeting? | 0=No, would never do this, 1=No, but would do if had the chance, 2=Yes, once or twice, 3=Yes,several times, 4=Yes, often, Missing Data = Don't Know, or refused to answer |
| Collective issue-raising | Here is a list of actions that people sometimes take as citizens. For each of these, please tell me whether you, personally, have done any of these things during the past year. If not, would you do this if you had the chance: Got together with others to raise an issue? | 0=No, would never do this, 1=No, but would do if had the chance, 2=Yes, once or twice, 3=Yes,several times, 4=Yes, often, Missing Data = Don't Know, or refused to answer |
| Protest | Here is a list of actions that people sometimes take as citizens. For each of these, please tell me whether you, personally, have done any of these things during the past year. If not, would you do this if you had the chance: Attended a demonstration or protest march? | 0=No, would never do this, 1=No, but would do if had the chance, 2=Yes, once or twice, 3=Yes,several times, 4=Yes, often, Missing Data = Don't Know, or refused to answer |

*Note*: The questions and answers coding are drawn from Afrobarometer questionnaire Round 5, accessed at https://www.afrobarometer.org/geocoded-data/.

**Supplementary Table 3** Descriptive statistics: Full sample

| Variable | Obs | Mean | SD | Min | Max |
|---|---|---|---|---|---|
| *Dependent Variables* | | | | | |
| Voting (dummy) | 90,419 | 0.802 | 0.399 | 0 | 1 |
| Contacting local government | 96,262 | 0.476 | 0.889 | 0 | 3 |
| Community meetings attendance | 100,857 | 2.180 | 1.316 | 0 | 4 |
| Collective issue-raising | 100,649 | 1.906 | 1.315 | 0 | 4 |
| Protest participation | 99,323 | 0.581 | 0.875 | 0 | 4 |
| *Covariates: Demographics* | | | | | |
| Urban (dummy) | 101,292 | 0.380 | 0.485 | 0 | 1 |
| Male (dummy) | 101,292 | 0.499 | 0.500 | 0 | 1 |
| Age | 100,247 | 36.81 | 14.45 | 18 | 130 |
| Education level | 101,042 | 3.223 | 2.108 | 0 | 9 |
| Employed (dummy) | 100,903 | 0.349 | 0.477 | 0 | 1 |
| Christian (dummy) | 101,292 | 0.349 | 0.477 | 0 | 1 |
| Muslim (dummy) | 101,292 | 0.191 | 0.393 | 0 | 1 |
| Black race (dummy) | 101,292 | 0.927 | 0.260 | 0 | 1 |
| *Covariances: Information and Infrastructure Accessibility* | | | | | |
| Owns radio | 101,178 | 0.705 | 0.456 | 0 | 1 |
| Owns TV | 101,093 | 0.385 | 0.487 | 0 | 1 |
| Post office access | 100,346 | 0.223 | 0.416 | 0 | 1 |
| Primary school access | 100,795 | 0.872 | 0.334 | 0 | 1 |
| Police station access | 100,245 | 0.342 | 0.474 | 0 | 1 |
| Tarred road access | 101,289 | 0.424 | 0.494 | 0 | 1 |
| *Variables for Mechanism Analysis* | | | | | |
| Without income | 98,171 | 1.992 | 1.406 | 0 | 4 |
| Without food | 101,087 | 1.082 | 1.225 | 0 | 4 |
| Without water | 101,104 | 1.155 | 1.380 | 0 | 4 |
| Without medics | 100,832 | 1.211 | 1.294 | 0 | 4 |
| Local government performance | 85,957 | 2.498 | 0.923 | 1 | 4 |
| Conflict count | 86,386 | 0.254 | 3.841 | 0 | 211 |
| Being attacked | 99,136 | 0.167 | 0.547 | 0 | 4 |
| Public affairs interest | 100,070 | 1.812 | 1.132 | 0 | 5 |
| Political discussions | 100,149 | 1.147 | 0.983 | 0 | 5 |
| Environment concern | 100,537 | 0.0251 | 0.156 | 0 | 1 |

**Supplementary Table 4** Descriptive Statistics: Election subsample

| Variable | Obs | Mean | SD | Min | Max |
|---|---|---|---|---|---|
| *Dependent Variable* | | | | | |
| Voting (dummy) | 69,940 | 0.794 | 0.405 | 0 | 1 |
| *Covariances: Demographics* | | | | | |
| Urban (dummy) | 69,940 | 0.367 | 0.482 | 0 | 1 |
| Male (dummy) | 69,940 | 0.501 | 0.500 | 0 | 1 |
| Age | 69,151 | 38.09 | 14.19 | 18 | 105 |
| Education level | 69,749 | 3.171 | 2.141 | 0 | 9 |
| Employed (dummy) | 69,657 | 0.354 | 0.478 | 0 | 1 |
| Christian (dummy) | 69,940 | 0.329 | 0.470 | 0 | 1 |
| Muslim (dummy) | 69,940 | 0.218 | 0.413 | 0 | 1 |
| Black race (dummy) | 69,940 | 0.928 | 0.258 | 0 | 1 |
| *Covariances: Information and Infrastructure Accessibility* | | | | | |
| Owns radio | 69,868 | 0.713 | 0.453 | 0 | 1 |
| Owns TV | 69,796 | 0.395 | 0.489 | 0 | 1 |
| Post office access | 69,343 | 0.221 | 0.415 | 0 | 1 |
| Primary school access | 69,550 | 0.878 | 0.328 | 0 | 1 |
| Police station access | 69,192 | 0.346 | 0.476 | 0 | 1 |
| Tarred road access | 69,939 | 0.422 | 0.494 | 0 | 1 |
| *Variables for Constructing IVs* | | | | | |
| Land Cover Combustibility(Dummy) | 69,940 | 0.924 | 0.266 | 0 | 1 |
| Mean Temperature | 69,940 | 22.93 | 4.125 | 7.631 | 30.82 |
| Mean wind speed | 69,940 | 1.454 | 0.660 | 0.156 | 5.184 |
| Mean yearly precipitation | 69,940 | 1,113 | 699.9 | 26.77 | 7,570 |

**Supplementary Table 5** Descriptive statistics: Non-election subsample

| Variable | Obs | Mean | SD | Min | Max |
|---|---|---|---|---|---|
| *Dependent Variables* | | | | | |
| Contacting local government | 83,206 | 0.480 | 0.893 | 0 | 3 |
| Community meetings attendance | 83,206 | 2.168 | 1.307 | 0 | 4 |
| Collective issue-raising | 83,206 | 1.863 | 1.292 | 0 | 4 |
| Protest participation | 83,206 | 0.596 | 0.885 | 0 | 4 |
| *Covariates: Demographics* | | | | | |
| Urban (dummy) | 83,206 | 0.382 | 0.486 | 0 | 1 |
| Male (dummy) | 83,206 | 0.501 | 0.500 | 0 | 1 |
| Age | 82,365 | 36.68 | 14.45 | 18 | 130 |
| Education level | 83,018 | 3.235 | 2.114 | 0 | 9 |
| Employed (dummy) | 82,924 | 0.349 | 0.477 | 0 | 1 |
| Christian (dummy) | 83,206 | 0.354 | 0.478 | 0 | 1 |
| Muslim (dummy) | 83,206 | 0.191 | 0.393 | 0 | 1 |
| Black race (dummy) | 83,206 | 0.929 | 0.258 | 0 | 1 |
| *Covariates: Information and Infrastructure Accessibility* | | | | | |
| Owns radio | 83,114 | 0.709 | 0.454 | 0 | 1 |
| Owns TV | 83,049 | 0.390 | 0.488 | 0 | 1 |
| Post office access | 82,427 | 0.220 | 0.414 | 0 | 1 |
| Primary school access | 82,817 | 0.874 | 0.332 | 0 | 1 |
| Police station access | 82,354 | 0.342 | 0.474 | 0 | 1 |
| Tarred road access | 83,204 | 0.416 | 0.493 | 0 | 1 |
| *Instrumental Variables* | | | | | |
| Land Cover Combustibility (Dummy) | 83,206 | 0.913 | 0.281 | 0 | 1 |
| Mean Temperature | 83,206 | 23.02 | 4.076 | 7.461 | 30.62 |
| Mean wind speed | 83,206 | 1.457 | 0.703 | 0.152 | 5.390 |
| Mean yearly precipitation | 83,206 | 1,122 | 665.0 | 26.85 | 7,550 |
| *Mechanism Variables* | | | | | |
| Without income | 80,569 | 1.980 | 1.410 | 0 | 4 |
| Without food | 83,061 | 1.069 | 1.218 | 0 | 4 |
| Without water | 83,086 | 1.159 | 1.376 | 0 | 4 |
| Without medics | 82,868 | 1.199 | 1.287 | 0 | 4 |
| Local government performance | 73,669 | 2.506 | 0.922 | 1 | 4 |
| Conflict count | 71,182 | 0.248 | 4.083 | 0 | 211 |
| Being attacked | 82,028 | 0.166 | 0.546 | 0 | 4 |
| Public affairs interest | 82,318 | 1.812 | 1.127 | 0 | 5 |
| Political discussions | 82,368 | 1.151 | 0.985 | 0 | 5 |
| Environment concern | 82,620 | 0.0242 | 0.154 | 0 | 1 |

**Supplementary Table 6** Landcover Types and Corresponding Codes

| Landcover Type | Code | Explanation |
|---|---|---|
| No data / Fill value | 0 | No data, used as fill value. |
| Bare land / Permanent snow or ice | 1 | Sand, rock, soil, or permanent snow/ice with less than 10% vegetation cover. |
| Permanent snow and ice | 2 | At least 60% of the area is covered by snow and ice for at least 10 months of the year. |
| Water bodies | 3 | At least 60% of the area is covered by permanent water bodies. |
| Urban and built-up lands | 9 | At least 30% of the area consists of impervious surfaces, including buildings, asphalt, and vehicles. |
| Dense forests | 10 | Tree cover exceeds 60%, with canopy height greater than 2 meters. |
| Open forests | 20 | Tree cover ranges from 10% to 60%, with canopy height greater than 2 meters. |
| Forest/cropland mosaics | 25 | Mosaics of small-scale cultivation (40%–60%) with more than 10% natural tree cover. |
| Natural herbaceous | 30 | Dominated by herbaceous annual plants shorter than 2 meters, with at least 10% cover. |
| Natural herbaceous/cropland mosaics | 35 | Mosaics of small-scale cultivation (40%–60%) mixed with natural shrub or herbaceous vegetation. |
| Herbaceous croplands | 36 | Dominated by herbaceous annual plants shorter than 2 meters, with at least 60% cover and cultivated fraction above 60%. |
| Shrublands | 40 | Shrub cover exceeds 60%, with vegetation height between 1 and 2 meters. |

*Notes*: This table presents the definition of land cover types and how they are coded in our research. The data is sourced from MODIS and processed by Google Earth Engine.

## 2 Sensitivity Analysis

**Supplementary Table 7** Baseline Results Accounting for Holm Correction

| | Institutionalized Form | | | Non-institutionalized Form | |
|---|---|---|---|---|---|
| Dept. Vars. | Voting | Contacting local govt. | Attending com. meetings | Collective issue-raising | Protest |
| | (1) | (2) | (3) | (4) | (5) |
| Panel A: OLS Estimation | | | | | |
| Wildfire | 0.0045 | 0.0433*** | 0.0668*** | 0.0486*** | 0.0127 |
| | (0.0045) | (0.0145) | (0.0183) | (0.0156) | (0.0123) |
| Observations | 69,940 | 83,206 | 83,206 | 83,206 | 83,206 |
| Original P-value | 0.317 | 0.003 | 0.000 | 0.002 | 0.301 |
| Corrected P-value | 0.317 | 0.204 | 0.000 | 0.032 | 0.602 |
| Panel B: 2SLS Estimation | | | | | |
| Wildfire | 0.0728** | 0.1490* | 0.3750*** | 0.2080** | 0.0373 |
| | (0.0290) | (0.0767) | (0.0975) | (0.0830) | (0.0683) |
| Observations | 69,940 | 83,206 | 83,206 | 83,206 | 83,206 |
| Original P-value | 0.012 | 0.053 | 0.000 | 0.013 | 0.585 |
| Corrected P-value | 0.048 | 0.106 | 0.000 | 0.039 | 0.585 |
| Country-by-Year FE | Yes | Yes | Yes | Yes | Yes |
| Spatial Cut-off (miles) | 20 | 20 | 20 | 20 | 20 |
| Temporal Cut-off (months) | 24 | 24 | 24 | 24 | 24 |

*Note*: Panel A reports the OLS estimation results, panel B reports the 2sls estimation results with key independent variables instrumented by climate conditions in the last 2 years and land cover combustibility in the 3rd year prior to the interview. This table adopts the Holm correction to account for the family-wise error rate [1]. The non-binary dependent variables have been standardized before estimation. We report the coefficients from the estimations based on Equation (3) and Equation (5). The variable *Wildfire* is a binary indicator equal to one if a wildfire occurred within 5 miles of the respondent's village in two years preceding the survey, and zero if the nearest wildfire occurred between 7 miles and 20 miles during the same period. All specifications include county-by-year fixed effects to account for unobserved heterogeneity and national-level temporal trends. Robust standard errors are clustered at the subnational level, with their standardized form reported in parentheses. Statistical inference of coefficients is based on two-sided t-tests. *** $p<0.01$, ** $p<0.05$, * $p<0.1$.

**Supplementary Table 8** Robustness Check: Different Measurements of Wildfire Exposure

| Dept. Vars. | Institutionalized Form | | | Non-institutionalized Form | |
|---|---|---|---|---|---|
| | Voting | Contacting local govt. | Attending com. meetings | Collective issue-raising | Protest |
| | (1) | (2) | (3) | (4) | (5) |
| **Panel A: Weighted average number of wildfires** | | | | | |
| Weighted wildfire number | 0.0029*** | 0.0051* | 0.0140*** | 0.0081*** | 0.0019 |
| | (0.0011) | (0.0027) | (0.0038) | (0.0031) | (0.0026) |
| Observations | 97,084 | 83,206 | 83,206 | 83,206 | 83,206 |
| **Panel B: Weighted average burned area of wildfires** | | | | | |
| Weighted wildfire area | 0.0003*** | 0.0005* | 0.0014*** | 0.0008** | 0.0002 |
| | (0.0001) | (0.0003) | (0.0004) | (0.0003) | (0.0003) |
| Observations | 97,084 | 83,206 | 83,206 | 83,206 | 83,206 |
| Country-Year FE | Yes | Yes | Yes | Yes | Yes |
| Spatial Cut-off (miles) | 20 | 20 | 20 | 20 | 20 |
| Temporal Cut-off (months) | 24 | 24 | 24 | 24 | 24 |

*Note*: This table reports the 2SLS estimation results with key independent variables instrumented by climate conditions in the last 3 years and landcover combustibility in the 3rd year prior to the interview. The non-binary dependent variables have been standardized before estimation. We report the coefficients from the estimations based on Equation (5). In Panel A, the variable of *Weighted wildfire number* is weighted average of the number of wildfires, calculated by Equation (1), while the variable of *Weighted wildfire area* in Panel B is the weighted average area of wildfires, calculated by Equation (2). All specifications include county-by-year fixed effects to account for unobserved heterogeneity and national-level temporal trends. Robust standard errors are clustered at the subnational level, with their standardized form reported in parentheses. Statistical inference of coefficients is based on two-sided t-tests. *** p<0.01, ** p<0.05, * p<0.1

**Supplementary Table 9** Robustness Check: Different Measurements of Wildfire Exposure—OLS

| | Institutionalized Form | | | Non-institutionalized Form | |
|---|---|---|---|---|---|
| Dept. Vars. | Voting | Contacting local govt. | Attending com. meetings | Collective issue-raising | Protest |
| | (1) | (2) | (3) | (4) | (5) |
| **Panel A: Weighted average number of wildfires** | | | | | |
| Weighted wildfire number | -0.0002 | 0.0013** | 0.0034*** | 0.0029*** | 0.0006 |
| | (0.0002) | (0.0007) | (0.0010) | (0.0009) | (0.0006) |
| Observations | 97,084 | 83,206 | 83,206 | 83,206 | 83,206 |
| **Panel B: Weighted average burned area of wildfires** | | | | | |
| Weighted wildfire area | 0.00001 | 0.00008* | 0.00017*** | 0.00015*** | 0.00002 |
| | (0.00001) | (0.00004) | (0.00005) | (0.00004) | (0.00003) |
| Observations | 97,084 | 83,206 | 83,206 | 83,206 | 83,206 |
| Country-Year FE | Yes | Yes | Yes | Yes | Yes |
| Spatial Cut-off (miles) | 20 | 20 | 20 | 20 | 20 |
| Temporal Cut-off (months) | 24 | 24 | 24 | 24 | 24 |

*Note*: This table reports the OLS estimation results. The non-binary dependent variables have been standardized before estimation. We report the coefficients from the estimations based on Equation (3). In Panel A, the variable of *Weighted wildfire number* is weighted average of the number of wildfires, calculated by Equation (1), while the variable of *Weighted wildfire area* in Panel B is the weighted average area of wildfires, calculated by Equation (2). All specifications include county-by-year fixed effects to account for unobserved heterogeneity and national-level temporal trends. Robust standard errors are clustered at the subnational level, with their standardized form reported in parentheses. Statistical inference of coefficients is based on two-sided t-tests. *** p<0.01, ** p<0.05, * p<0.1

**Supplementary Table 10** Robustness Check: Uneven Intervals

| Dept. Vars. | Institutionalized Form | | | Non-institutionalized Form | |
|---|---|---|---|---|---|
| | Voting | Contacting local govt. | Attending com. meetings | Collective issue-raising | Protest |
| | (1) | (2) | (3) | (4) | (5) |
| **Panel A: 2SLS Estimation Results** | | | | | |
| Wildfire [0,5) | 0.1060*** | 0.1530** | 0.3720*** | 0.2060** | 0.0354 |
| | (0.0339) | (0.0762) | (0.0974) | (0.0827) | (0.0681) |
| Wildfire [5,20) | -0.0324** | 0.0538 | 0.1350* | 0.0531 | 0.0214 |
| | (0.0132) | (0.0703) | (0.0768) | (0.0637) | (0.0696) |
| Wildfire [20,50] | 0.0039 | -0.1280** | 0.0002 | 0.0127 | 0.0329 |
| | (0.0212) | (0.0516) | (0.0718) | (0.0615) | (0.0499) |
| Observations | 97,084 | 83,206 | 83,206 | 83,206 | 83,206 |
| **Panel B: OLS Estimation Results** | | | | | |
| Wildfire [0,5) | 0.0056 | 0.0444*** | 0.0651*** | 0.0473*** | 0.0123 |
| | (0.0039) | (0.0145) | (0.0185) | (0.0157) | (0.0124) |
| Wildfire [5,20) | 0.0018 | -0.0084 | -0.0402 | -0.0378 | 0.0082 |
| | (0.0062) | (0.0551) | (0.0597) | (0.0488) | (0.0571) |
| Wildfire [20,50] | 0.0097 | -0.0982** | 0.0826 | 0.0555 | 0.0391 |
| | (0.0193) | (0.0459) | (0.0653) | (0.0585) | (0.0465) |
| Observations | 97,084 | 83,206 | 83,206 | 83,206 | 83,206 |
| Country-Year FE | Yes | Yes | Yes | Yes | Yes |
| Spatial Cut-off (miles) | 50 | 50 | 50 | 50 | 50 |
| Temporal Cut-off (months) | 24 | 24 | 24 | 24 | 24 |

*Note*: Panel A reports the 2SLS estimation results with key independent variables instrumented by climate conditions in the last 2 years and landcover combustibility in the 3rd year prior to the interview (notice that only $Wildfire[0,5)$ is instrumented), panel B reports the OLS estimation results. The non-binary dependent variables have been standardized before estimation. We report the coefficients from the estimations based on Equation (5) and Equation (3). *Wildfire [0,5)*, *Wildfire [5,20)*, and *Wildfire [20,50]* is a series of binary indicators equal to one if a wildfire occurred within 0–5, 5–20, and 20–50 miles of the respondent's village in two years preceding the survey, respectively, and zero otherwise. All specifications include county-by-year fixed effects to account for unobserved heterogeneity and national-level temporal trends. Robust standard errors reported in parentheses are clustered at the subnational level. Statistical inference of coefficients is based on two-sided t-tests. *** p<0.01, ** p<0.05, * p<0.1.

**Supplementary Table 11** Robustness Check: Continuous Measurements of Wildfire Distance

| | Institutionalized Form | | | Non-institutionalized Form | |
|---|---|---|---|---|---|
| Dept. Vars. | Voting | Contacting local govt. | Attending com. meetings | Collective issue-raising | Protest |
| | (1) | (2) | (3) | (4) | (5) |
| **Panel A: 2SLS Estimation Results** | | | | | |
| Distance (miles) | -0.0041*** | -0.0097* | -0.0254*** | -0.0139** | -0.0026 |
| | (0.0015) | (0.0058) | (0.0075) | (0.0061) | (0.0049) |
| Observations | 97,084 | 83,206 | 83,206 | 83,206 | 83,206 |
| **Panel B: OLS Estimation Results** | | | | | |
| Distance (miles) | -0.0001 | -0.0012 | -0.0037*** | -0.0024** | -0.0004 |
| | (0.0002) | (0.0008) | (0.0011) | (0.0010) | (0.0008) |
| Observations | 97,084 | 83,206 | 83,206 | 83,206 | 83,206 |
| Country-Year FE | Yes | Yes | Yes | Yes | Yes |
| Spatial Cut-off (miles) | 50 | 50 | 50 | 50 | 50 |
| Temporal Cut-off (months) | 24 | 24 | 24 | 24 | 24 |

*Note*: This table reports the estimation results of applying distance between village and its nearest wildfire as key independent variable. The non-binary dependent variables have been standardized before estimation. We report the coefficients from the estimations based on Equation (5). All specifications include county-by-year fixed effects to account for unobserved heterogeneity and national-level temporal trends. Robust standard errors are clustered at the subnational level, with their standardized form reported in parentheses. Statistical inference of coefficients is based on two-sided t-tests. *** $p<0.01$, ** $p<0.05$, * $p<0.1$

**Supplementary Table 12** Robustness Check: Sample without Combustible Land Cover due to Agriculture

| | Institutionalized Form | | | Non-institutionalized Form | |
|---|---|---|---|---|---|
| Dept. Vars. | Voting | Contacting local govt. | Attending com. meetings | Collective issue-raising | Protest |
| | (1) | (2) | (3) | (4) | (5) |
| Wildfire | 0.0776** | 0.1910** | 0.4030*** | 0.2110** | 0.0420 |
| | (0.0313) | (0.0837) | (0.1080) | (0.0899) | (0.0765) |
| Observations | 55,230 | 65,981 | 65,981 | 65,981 | 65,981 |
| Country-Year FE | Yes | Yes | Yes | Yes | Yes |
| Spatial Cut-off (miles) | 20 | 20 | 20 | 20 | 20 |
| Temporal Cut-off (months) | 24 | 24 | 24 | 24 | 24 |

*Notes:* This table reports the 2SLS estimation results of the same IV setting as in Equation 5, excluding samples in which land cover combustibility comes from agricultural activities. $Wildfire$ is a binary indicator equal to one if a wildfire occurred within 5 miles of the respondent's village in two years preceding the survey, and zero if the nearest wildfire occurred between 7 miles and 20 miles during the same period. Non-binary dependent variables have been standardized before estimation. We report the coefficients from the estimations based on Equation (5). All specifications include county-by-year fixed effects to account for unobserved heterogeneity and national-level temporal trends. Robust standard errors reported in parentheses are clustered at the subnational level. Statistical inference of coefficients is based on two-sided t-tests. *** p<0.01, ** p<0.05, * p<0.1.

**Supplementary Table 13** Robustness Check: Sample without Built-in Land Cover

| Dept. Vars. | Institutionalized Form | | | Non-institutionalized Form | |
|---|---|---|---|---|---|
| | Voting | Contacting local govt. | Attending com. meetings | Collective issue-raising | Protest |
| | (1) | (2) | (3) | (4) | (5) |
| Wildfire | 0.1180*** | 0.1630* | 0.3120*** | 0.1660* | -0.0136 |
| | (0.0413) | (0.0940) | (0.1090) | (0.0910) | (0.0713) |
| Observations | 66,319 | 78,628 | 78,628 | 78,628 | 78,628 |
| Country-Year FE | Yes | Yes | Yes | Yes | Yes |
| Spatial Cut-off (miles) | 20 | 20 | 20 | 20 | 20 |
| Temporal Cut-off (months) | 24 | 24 | 24 | 24 | 24 |

*Notes:* This table reports the 2SLS estimation results of the IV setting excluding samples with built-in or urban land-cover. $Wildfire$ is a binary indicator equal to one if a wildfire occurred within 5 miles of the respondent's village in two years preceding the survey, and zero if the nearest wildfire occurred between 7 miles and 20 miles during the same period. Non-binary dependent variables have been standardized before estimation. We report the coefficients from the estimations based on Equation (5). All specifications include county-by-year fixed effects to account for unobserved heterogeneity and national-level temporal trends. Robust standard errors reported in parentheses are clustered at the subnational level. Statistical inference of coefficients is based on two-sided t-tests. *** p<0.01, ** p<0.05, * p<0.1.

**Supplementary Table 14** Robustness Check: Sample without Built-in and Agricultural Land Cover

| Dept. Vars. | Institutionalized Form | | | Non-institutionalized Form | |
|---|---|---|---|---|---|
| | Voting | Contacting local govt. | Attending com. meetings | Collective issue-raising | Protest |
| | (1) | (2) | (3) | (4) | (5) |
| Wildfire | 0.1290*** | 0.2250** | 0.3400** | 0.1780 | -0.0001 |
| | (0.0485) | (0.1060) | (0.1320) | (0.1080) | (0.0823) |
| Observations | 51,609 | 61,403 | 61,403 | 61,403 | 61,403 |
| Country-Year FE | Yes | Yes | Yes | Yes | Yes |
| Spatial Cut-off (miles) | 20 | 20 | 20 | 20 | 20 |
| Temporal Cut-off (months) | 24 | 24 | 24 | 24 | 24 |

*Notes:* This table reports the 2SLS estimation results of the IV setting excluding samples with built-in, urban or agricultural land-cover. *Wildfire* is a binary indicator equal to one if a wildfire occurred within 5 miles of the respondent's village in two years preceding the survey, and zero if the nearest wildfire occurred between 7 miles and 20 miles during the same period. Non-binary dependent variables have been standardized before estimation. We report the coefficients from the estimations based on Equation (5). All specifications include county-by-year fixed effects to account for unobserved heterogeneity and national-level temporal trends. Robust standard errors reported in parentheses are clustered at the subnational level. Statistical inference of coefficients is based on two-sided t-tests. *** p<0.01, ** p<0.05, * p<0.1.

**Supplementary Table 15** Robustness Checks: Controlling for Quadratic and Cubic Terms of Temperature and Precipitation

| Dept. Vars. | Voting | Contacting local govt. | Attending com. meetings | Collective issue-raising | Protest |
|---|---|---|---|---|---|
| | (1) | (2) | (3) | (4) | (5) |
| **Panel A: Adding Quadratic Terms** | | | | | |
| Wildfire | 0.0675*** | 0.167** | 0.388*** | 0.214*** | 0.0199 |
| | (0.0257) | (0.0759) | (0.0929) | (0.0805) | (0.0678) |
| Observations | 69,940 | 83,206 | 83,206 | 83,206 | 83,206 |
| **Panel B: Adding Cubic Terms** | | | | | |
| Wildfire | 0.0664** | 0.166** | 0.389*** | 0.213*** | 0.0200 |
| | (0.0257) | (0.0770) | (0.0941) | (0.0817) | (0.0688) |
| Observations | 69,940 | 83,206 | 83,206 | 83,206 | 83,206 |
| **Panel C: Adding Both Cubic and Quadratic Terms** | | | | | |
| Wildfire | 0.0808*** | 0.162** | 0.390*** | 0.200** | 0.000464 |
| | (0.0284) | (0.0821) | (0.101) | (0.0874) | (0.0728) |
| Observations | 69,940 | 83,206 | 83,206 | 83,206 | 83,206 |
| Country-Year FE | Yes | Yes | Yes | Yes | Yes |
| Spatial Cut-off (miles) | 20 | 20 | 20 | 20 | 20 |
| Temporal Cut-off (months) | 24 | 24 | 24 | 24 | 24 |

*Note*: This table reports the 2SLS estimation results of adding high-order term of annual temperature and precipitation as covariates. Robust standard errors are reported in parentheses. All specifications include country-year fixed effects. The spatial cut-off is 20 miles and the temporal cut-off is 24 months. Statistical inference of coefficients is based on two-sided t-tests. *** p<0.01, ** p<0.05, * p<0.1.

**Supplementary Table 16** Robustness Check: Adding Controls

| Dept. Vars. | Institutionalized Form | | | Non-institutionalized Form | |
|---|---|---|---|---|---|
| | Voting | Contacting local govt. | Attending com. meetings | Collective issue-raising | Protest |
| | (1) | (2) | (3) | (4) | (5) |
| **Panel A: Controlling Individual Characteristics** | | | | | |
| Wildfire | 0.0621** | 0.1400* | 0.3190*** | 0.1950** | 0.0975 |
| | (0.0296) | (0.0761) | (0.0934) | (0.0857) | (0.0698) |
| Observations | 68,724 | 81,940 | 81,940 | 81,940 | 81,940 |
| Ind. Char. | Yes | Yes | Yes | Yes | Yes |
| **Panel B: Controlling Infrastructure and Information Accessibility** | | | | | |
| Wildfire | 0.0694** | 0.1650** | 0.3480*** | 0.2190** | 0.1310* |
| | (0.0307) | (0.0782) | (0.0961) | (0.0883) | (0.0702) |
| Observations | 67,402 | 80,327 | 80,327 | 80,327 | 80,327 |
| Ind. Char. | Yes | Yes | Yes | Yes | Yes |
| I. & I. | Yes | Yes | Yes | Yes | Yes |

*Notes:* This table reports the 2SLS estimation results with key independent variables instrumented by climate conditions in the last 2 years and land cover combustibility in the 3rd year prior to the interview. $Wildfire$ is a binary indicator equal to one if a wildfire occurred within 5 miles of the respondent's village in two years preceding the survey, and zero if the nearest wildfire occurred between 7 miles and 20 miles during the same period. Panel A controls for individual characteristics, including urban residency (binary), age (log-transformed), gender (binary), religion (Christians, Muslim or others), race (Binary for black or not), employment status (binary) and education level (factorized). Panel B controls for individual characteristics together with infrastructure and information accessibility, which is measured by tarred road (binary), post station(binary), police station (binary), primary school(binary) accessibility and whether respondents has their own TVs(binary) and radios(binary). Non-binary dependent variables have been standardized before estimation. We report the coefficients from the estimations based on Equation (5). All specifications include county-by-year fixed effects to account for unobserved heterogeneity and national-level temporal trends. Robust standard errors reported in parentheses are clustered at the subnational level. Statistical inference of coefficients is based on two-sided t-tests. *** p<0.01, ** p<0.05, * p<0.1.

**Supplementary Table 17** Robustness Check: Adding Controls—OLS

| Dept. Vars. | Institutionalized Form | | | Non-institutionalized Form | |
|---|---|---|---|---|---|
| | Voting | Contacting local govt. | Attending com. meetings | Collective issue-raising | Protest |
| | (1) | (2) | (3) | (4) | (5) |
| **Panel A: Controlling Individual Characteristics** | | | | | |
| Wildfire | 0.0050 | 0.0410*** | 0.0541*** | 0.0460*** | 0.0168 |
| | (0.0042) | (0.0136) | (0.0157) | (0.0144) | (0.0118) |
| Observations | 68,724 | 81,940 | 81,940 | 81,940 | 81,940 |
| Ind. Char. | Yes | Yes | Yes | Yes | Yes |
| **Panel B: Controlling Infrastructure and Information Accessibility** | | | | | |
| Wildfire | 0.0052 | 0.0426*** | 0.0557*** | 0.0473*** | 0.0174 |
| | (0.0042) | (0.0136) | (0.0156) | (0.0144) | (0.0117) |
| Observations | 67,402 | 80,327 | 80,327 | 80,327 | 80,327 |
| Ind. Char. | Yes | Yes | Yes | Yes | Yes |
| I. & I. | Yes | Yes | Yes | Yes | Yes |

*Notes:* This table reports the OLS estimation results. $Wildfire$ is a binary indicator equal to one if a wildfire occurred within 5 miles of the respondent's village in two years preceding the survey, and zero if the nearest wildfire occurred between 7 miles and 20 miles during the same period. Panel A controls for individual characteristics, including urban residency (binary), age (log-transformed), gender (binary), religion (Christians, Muslim or others), race (Binary for black or not), employment status (binary) and education level (factorized). Panel B controls for individual characteristics together with infrastructure and information accessibility, which is measured by tarred road (binary), post station(binary), police station (binary), primary school(binary) accessibility and whether respondents has their own TVs(binary) and radios(binary). Non-binary dependent variables have been standardized before estimation. We report the coefficients from the estimations based on Equation (3). All specifications include county-by-year fixed effects to account for unobserved heterogeneity and national-level temporal trends. Robust standard errors reported in parentheses are clustered at the subnational level. Statistical inference of coefficients is based on two-sided t-tests. *** $p<0.01$, ** $p<0.05$, * $p<0.1$.

**Supplementary Table 18** Robustness Check: First Stage of IV Estimation

| Dept. Vars. = Wildfire | Sample | |
|---|---|---|
| | Election | Non-election |
| | (1) | (2) |
| $Tem_{t-2} \cdot I(LC)_{t-3}$ | 0.0242*** | 0.0197** |
| | (0.0088) | (0.0095) |
| $Wind_{t-2}$ | -0.1610*** | -0.1640*** |
| | (0.0248) | (0.0201) |
| $Prep_{t-2} \cdot I(LC)_{t-3}$ | 0.6780** | 0.0588 |
| | (0.3190) | (0.0504) |
| $Tem_{t-2}$ | 0.0294*** | 0.0207** |
| | (0.0086) | (0.0089) |
| $Prep_{t-2}$ | 0.4070 | 0.0464 |
| | (0.3060) | (0.0401) |
| $I(LC)_{t-3}$ | -1.6420*** | -0.6920* |
| | (0.5960) | (0.3800) |
| F-statistic | 26.74 | 31.44 |
| Observations | 69,940 | 83,206 |
| Country-Year FE | Yes | Yes |
| Spatial Cut-off (miles) | 20 | 20 |
| Temporal Cut-off (months) | 24 | 24 |

*Note*: This table reports the first stage estimation results of the IV estimation reported in panel B of Main Table 1. Annual precipitation ($Prep_{t-2}$) is log-transformed. And the land cover combustibility ($I(LC)_{t-3}$) is standardized by sample mean. The *F Statistic* reports the joint F test results of excluded IVs. All specifications include county-by-year fixed effects to account for unobserved heterogeneity and national-level temporal trends. Robust standard errors are clustered at the subnational level, with their standardized form reported in parentheses. Statistical inference of coefficients is based on two-sided t-tests. *** p<0.01, ** p<0.05, * p<0.1.

**Supplementary Table 19** Robustness Check: Reduced Estimation Results of Dependent Variables on IVs

| | Institutionalized Form | | | Non-institutionalized Form | |
|---|---|---|---|---|---|
| Dept. Vars. | Voting | Contacting local govt. | Attending com. meetings | Collective issue-raising | Protest |
| | (1) | (2) | (3) | (4) | (5) |
| $Tem_{t-2} \cdot I(LC)_{t-3}$ | -0.0004 | 0.0017 | 0.0164** | 0.0123* | 0.0065 |
| | (0.0025) | (0.0055) | (0.0083) | (0.0071) | (0.0056) |
| $Wind_{t-2}$ | -0.0162*** | -0.0228 | -0.0469*** | -0.0216 | 0.0008 |
| | (0.0060) | (0.0158) | (0.0177) | (0.0151) | (0.0119) |
| $Prep_{t-2} \cdot I(LC)_{t-3}$ | -0.0216 | 0.0480* | 0.0721* | 0.0491 | 0.0043 |
| | (0.0647) | (0.0268) | (0.0390) | (0.0375) | (0.0348) |
| $Tem_{t-2}$ | 0.0047*** | 0.0061 | 0.0185*** | 0.0144*** | 0.0120*** |
| | (0.0018) | (0.0045) | (0.0060) | (0.0049) | (0.0045) |
| $Prep_{t-2}$ | 0.0387 | 0.0258 | 0.0458 | 0.0867*** | 0.0826*** |
| | (0.0639) | (0.0216) | (0.0293) | (0.0252) | (0.0230) |
| $I(LC)_{t-3}$ | 0.0703 | -0.2660 | -0.6760** | -0.5270* | -0.1550 |
| | (0.1400) | (0.2080) | (0.3240) | (0.2720) | (0.2840) |
| Observations | 69,940 | 83,206 | 83,206 | 83,206 | 83,206 |
| Country-Year FE | Yes | Yes | Yes | Yes | Yes |
| Spatial Cut-off (miles) | 50 | 50 | 50 | 50 | 50 |
| Temporal Cut-off (months) | 24 | 24 | 24 | 24 | 24 |

*Note*: This table reports the OLS estimation results of regressing dependent variables over IVs directly, while also controlling for covariates as in baseline specification (Equation 3). The non-binary dependent variables have been standardized before estimation. Annual precipitation ($Prep_{t-2}$) is log-transformed. And the land cover combustibility ($I(LC)_{t-3}$) is standardized by sample mean. All specifications include county-by-year fixed effects to account for unobserved heterogeneity and national-level temporal trends. Robust standard errors are clustered at the subnational level, with their standardized form reported in parentheses. Statistical inference of coefficients is based on two-sided t-tests. *** p<0.01, ** p<0.05, * p<0.1.

**Supplementary Table 20** Robustness Check: Falsification Test of Instrumental Variables

| | Panel A: Material Deprivation and Government Performance | | | | |
|---|---|---|---|---|---|
| | Without Food | Without Income | Without Medics | Without Water | Local Govt. Perf. |
| | (1) | (2) | (3) | (4) | (5) |
| $Tem_{v(i),t-2} \cdot I(LC)_{v(i),t-3}$ | 0.0054 | 0.0130* | 0.0103* | -0.0041 | 0.0038 |
| | (0.0059) | (0.0069) | (0.0058) | (0.0090) | (0.0073) |
| $Windspeed_{v(i),t-2}$ | 0.0131 | -0.0078 | -0.0155 | -0.0212 | -0.0259 |
| | (0.0219) | (0.0181) | (0.0186) | (0.0208) | (0.0160) |
| $Prep_{v(i),t-2} \cdot I(LC)_{v(i),t-3}$ | -0.0225 | -0.0211 | 0.0619 | 0.0128 | -0.0223 |
| | (0.0377) | (0.0374) | (0.0391) | (0.0538) | (0.0529) |
| Observations | 83,061 | 80,569 | 82,868 | 83,086 | 73,669 |
| F-statistic | 0.4746 | 1.3064 | 2.8260 | 0.4388 | 1.1673 |
| | Panel B: Conflict, Crime, Environmental and Political Concern | | | | |
| | Conflict Count | Being Attacked | Env. Concern | Pub. Aff. Int. | Pol. Discussion |
| | (6) | (7) | (8) | (9) | (10) |
| $Tem_{v(i),t-2} \cdot I(LC)_{v(i),t-3}$ | -0.0134 | -0.0015 | 0.0018** | 0.0043 | 0.0023 |
| | (0.0130) | (0.0035) | (0.0008) | (0.0053) | (0.0055) |
| $Windspeed_{v(i),t-2}$ | 0.0578 | 0.0120 | 0.0024 | 0.0125 | 0.0047 |
| | (0.0475) | (0.0079) | (0.0023) | (0.0134) | (0.0124) |
| $Prep_{v(i),t-2} \cdot I(LC)_{v(i),t-3}$ | -0.3190* | 0.0195 | -0.0152*** | 0.0607* | 0.0457 |
| | (0.1750) | (0.0224) | (0.0045) | (0.0341) | (0.0301) |
| Observations | 71,182 | 82,028 | 26,206 | 82,318 | 82,368 |
| F-statistic | 1.2125 | 1.1173 | 4.8227 | 1.8093 | 0.8767 |
| Country-by-Year FE | Yes | Yes | Yes | Yes | Yes |
| Spatial Cut-off (miles) | 20 | 20 | 20 | 20 | 20 |
| Temporal Cut-off (months) | 24 | 24 | 24 | 24 | 24 |

*Note*: This table reports falsification tests regressing potential mechanism variables on instrumental variables constructed from climatic conditions. All specifications include country-by-year fixed effects. Robust standard errors clustered at the regional level are reported in parentheses. The F-statistic test the joint null hypothesis that the coefficients on $Tem_{v(i),t-2} \cdot I(LC)_{v(i),t-3}$, $Windspeed_{v(i),t-2}$, and $Prep_{v(i),t-2} \cdot I(LC)_{v(i),t-3}$ are jointly zero. Statistical inference of coefficients is based on two-sided t-tests. *** p<0.01, ** p<0.05, * p<0.1.

**Supplementary Table 21** Robustness Check: Different Estimated Models

| Dept. Vars. | Institutionalized Form | | | Non-institutionalized Form | |
|---|---|---|---|---|---|
| | Voting | Contacting local govt. | Attending com. meetings | Collective issue-raising | Protest |
| | (1) | (2) | (3) | (4) | (5) |
| **Panel A: Ordered Probit Model** | | | | | |
| Wildfire | 0.0188 | 0.0604*** | 0.0750*** | 0.0561*** | 0.0164 |
| | (0.0166) | (0.0199) | (0.0202) | (0.0177) | (0.0152) |
| Observations | 69,886 | 83,206 | 83,206 | 83,206 | 83,206 |
| **Panel B: Ordered Instrumented Probit Model** | | | | | |
| Wildfire | 0.0401 | 0.2360** | 0.4090*** | 0.2280** | 0.0437 |
| | (0.1180) | (0.1100) | (0.1090) | (0.0978) | (0.0800) |
| Observations | 78,227 | 83,206 | 83,206 | 83,206 | 83,206 |
| Country-Year FE | Yes | Yes | Yes | Yes | Yes |
| Spatial Cut-off (miles) | 20 | 20 | 20 | 20 | 20 |
| Temporal Cut-off (months) | 24 | 24 | 24 | 24 | 24 |

*Note*: Panel A uses the Ordered Probit Model, while Panel B uses an ordered probit model instrumented by climate conditions [2]. The variable *Wildfire* is a binary indicator equal to one if a wildfire occurred within 5 miles of the respondent's village in two years preceding the survey, and zero if the nearest wildfire occurred between 7 miles and 20 miles during the same period. All specifications include county-by-year fixed effects to account for unobserved heterogeneity and national-level temporal trends. In panel B, odds ratios are reported instead of coefficients. Robust standard errors are clustered at the subnational level. Statistical inference of coefficients is based on two-sided t-tests. *** p<0.01, ** p<0.05, * p<0.1.

# 3 Mechanism Analysis

**Supplementary Table 22** Mechanism Analysis—OLS

| | | | | | | |
|---|---|---|---|---|---|---|
| | Panel A: Material Deprivation and Government Performance | | | | | |
| | Material Deprivation | | | | Percep. Local Govt. | |
| Dep. Vars. | Without Food | Without Water | Without Medics | Without Income | Local Perf. | Govt. |
| | (1) | (2) | (3) | (4) | (5) | |
| Wildfire | 0.0267 | 0.0616** | 0.0816*** | 0.0566*** | 0.0281* | |
| | (0.0179) | (0.0307) | (0.0163) | (0.0164) | (0.0166) | |
| Observations | 83,061 | 26,347 | 82,868 | 80,569 | 73,669 | |
| | Panel B: Conflict, Crime and Concern | | | | | |
| | Conflict and Crime | | Environmental / Political Concern | | | |
| Dep. Vars. | Conflict Count | Being Attacked | Env. Concern | Pub. Aff. Int. | Pol. Discussion | |
| | (6) | (7) | (8) | (9) | (10) | |
| Wildfire | 0.0263 | 0.0021 | -0.0055 | 0.0027 | -0.0062 | |
| | (0.0474) | (0.0075) | (0.0033) | (0.0108) | (0.0103) | |
| Observations | 71,182 | 82,028 | 26,206 | 82,318 | 82,368 | |
| Country-by-Year FE | Yes | Yes | Yes | Yes | Yes | |
| Spatial Cut-off (miles) | 20 | 20 | 20 | 20 | 20 | |
| Temporal Cut-off (months) | 24 | 24 | 24 | 24 | 24 | |

*Note*: This table presents mechanism analysis using outcome variables matched to wildfire exposure in the prior two years (OLS). Robust SEs clustered at the subnational level. Statistical inference of coefficients is based on two-sided t-tests. *** p<0.01, ** p<0.05, * p<0.1.

# 4 Heterogeneity Analysis

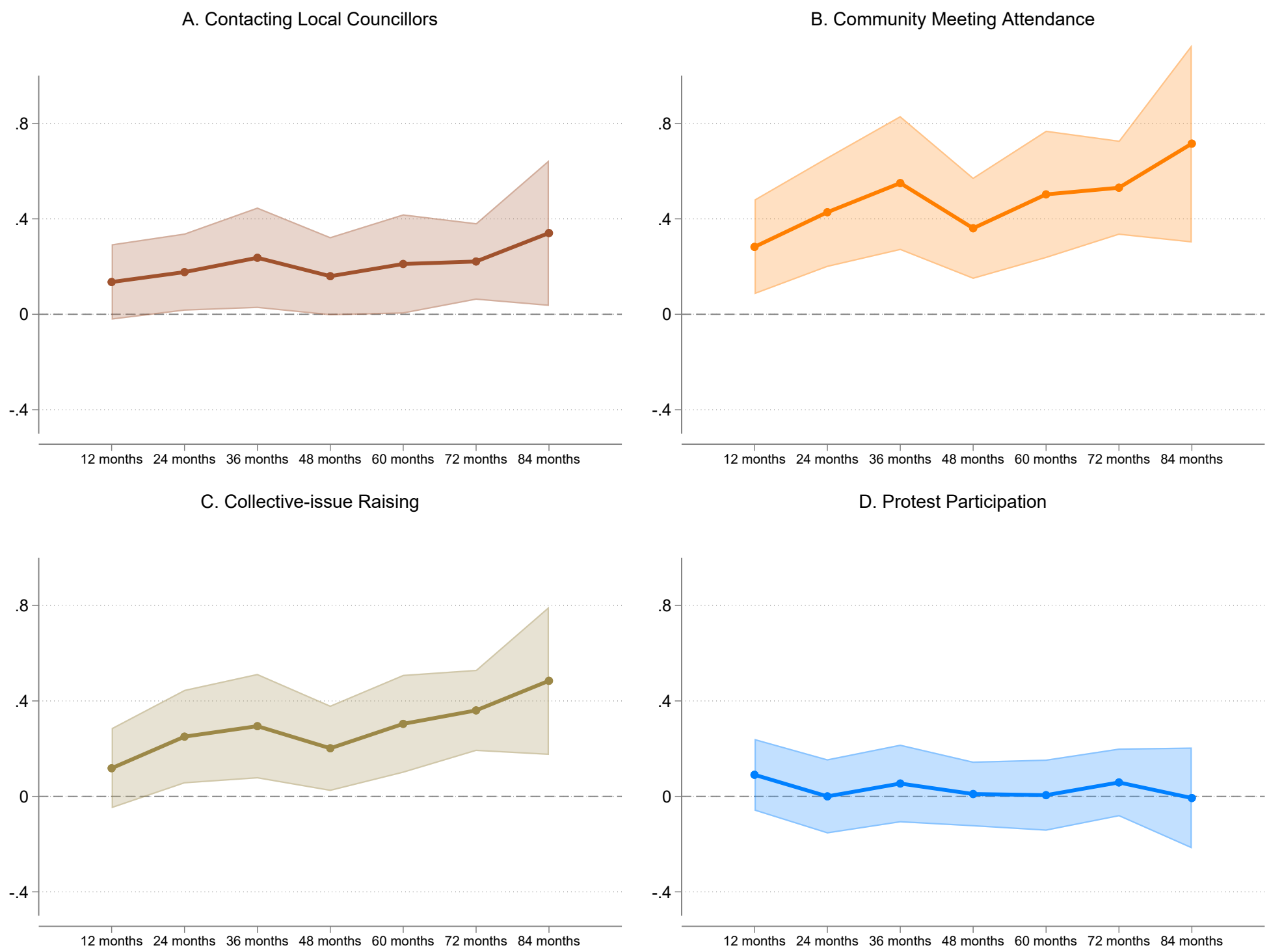


**Supplementary Figure 1** The Long-term Effect of Wildfire Exposure on Political Participation

Note: This coefficient plot reports separate 2SLS estimates for exposure windows of 12, 24, 36, 48, 60, 72, and 84 months; the shaded areas show 95% confidence intervals. The outcomes are (A) contacting local councillors, (B) attending community meetings, (C) collective issue-raising, and (D) protest participation. Sample sizes, coefficients and other information are reported through Supplementary Table 23–26. Statistical inference of coefficients is based on two-sided t-tests.

**Supplementary Table 23** Long-term Effects on Contacting Local Councillors

| Dept. Vars = Contacting local govt. | (1) | (2) | (3) | (4) | (5) | (6) | (7) |
|---|---|---|---|---|---|---|---|
| Wildfire [0m,12m] | 0.1350* | | | | | | |
| | (0.0806) | | | | | | |
| Wildfire (12m,24m] | | 0.1770** | | | | | |
| | | (0.0825) | | | | | |
| Wildfire (24m,36m] | | | 0.2370** | | | | |
| | | | (0.1070) | | | | |
| Wildfire (36m,48m] | | | | 0.1600* | | | |
| | | | | (0.0836) | | | |
| Wildfire (48m,60m] | | | | | 0.2110** | | |
| | | | | | (0.1060) | | |
| Wildfire (60m,72m] | | | | | | 0.2210*** | |
| | | | | | | (0.0818) | |
| Wildfire (72m,84m] | | | | | | | 0.3410** |
| | | | | | | | (0.1560) |
| Observations | 71,053 | 68,650 | 55,807 | 51,156 | 49,637 | 46,543 | 51,208 |
| Country-Year FE | Yes | Yes | Yes | Yes | Yes | Yes | Yes |
| Spatial Cut-off | 20 miles | 20 miles | 20 miles | 20 miles | 20 miles | 20 miles | 20 miles |
| Temporal Cut-off | 24 months | 24 months | 24 months | 24 months | 24 months | 24 months | 24 months |

*Note*: The dependent variables have been standardized before estimation. We report the coefficients from the estimations based on Equation (5). The variable *Wildfire* is a binary indicator equal to one if a wildfire occurred within 5 miles of the respondent's village in two years preceding the survey, and zero if the nearest wildfire occurred between 7 miles and 20 miles during the same period. All specifications include county-by-year fixed effects to account for unobserved heterogeneity and national-level temporal trends. Robust standard errors are clustered at the subnational level. Statistical inference of coefficients is based on two-sided t-tests. *** $p<0.01$, ** $p<0.05$, * $p<0.1$.

**Supplementary Table 24** Long-term Effects on Community Meeting Attendance

| Dept. Vars = Community meeting attendance | (1) | (2) | (3) | (4) | (5) | (6) | (7) |
|---|---|---|---|---|---|---|---|
| Wildfire [0m,12m] | 0.2820*** | | | | | | |
| | (0.1010) | | | | | | |
| Wildfire (12m,24m] | | 0.4280*** | | | | | |
| | | (0.1170) | | | | | |
| Wildfire (24m,36m] | | | 0.5500*** | | | | |
| | | | (0.1430) | | | | |
| Wildfire (36m,48m] | | | | 0.3610*** | | | |
| | | | | (0.1080) | | | |
| Wildfire (48m,60m] | | | | | 0.5020*** | | |
| | | | | | (0.1360) | | |
| Wildfire (60m,72m] | | | | | | 0.5300*** | |
| | | | | | | (0.1010) | |
| Wildfire (72m,84m] | | | | | | | 0.7150*** |
| | | | | | | | (0.2110) |
| Observations | 71,053 | 63,031 | 55,807 | 51,156 | 49,637 | 46,543 | 51,208 |
| Country-Year FE | Yes | Yes | Yes | Yes | Yes | Yes | Yes |
| Spatial Cut-off | 20 miles | 20 miles | 20 miles | 20 miles | 20 miles | 20 miles | 20 miles |
| Temporal Cut-off | 24 months | 24 months | 24 months | 24 months | 24 months | 24 months | 24 months |

*Note*: The dependent variables have been standardized before estimation. We report the standardized coefficients from the estimations based on Equation (5). The variable *Wildfire* is a binary indicator equal to one if a wildfire occurred within 5 miles of the respondent's village in two years preceding the survey, and zero if the nearest wildfire occurred between 7 miles and 20 miles during the same period. All specifications include county-by-year fixed effects to account for unobserved heterogeneity and national-level temporal trends. Robust standard errors are clustered at the subnational level. Statistical inference of coefficients is based on two-sided t-tests. *** $p<0.01$, ** $p<0.05$, * $p<0.1$

**Supplementary Table 25** Long-term Effects on Collective Issue-raising

| Dept. Vars = Collective issue-raising | (1) | (2) | (3) | (4) | (5) | (6) | (7) |
|---|---|---|---|---|---|---|---|
| Wildfire [0m,12m] | 0.1180 | | | | | | |
| | (0.0856) | | | | | | |
| Wildfire (12m,24m] | | 0.2500** | | | | | |
| | | (0.1000) | | | | | |
| Wildfire (24m,36m] | | | 0.2940*** | | | | |
| | | | (0.1120) | | | | |
| Wildfire (36m,48m] | | | | 0.2020** | | | |
| | | | | (0.0912) | | | |
| Wildfire (48m,60m] | | | | | 0.3040*** | | |
| | | | | | (0.1050) | | |
| Wildfire (60m,72m] | | | | | | 0.3600*** | |
| | | | | | | (0.0867) | |
| Wildfire (72m,84m] | | | | | | | 0.4850*** |
| | | | | | | | (0.1580) |
| Observations | 71,053 | 68,650 | 55,807 | 51,156 | 49,637 | 46,543 | 51,208 |
| Country-Year FE | Yes | Yes | Yes | Yes | Yes | Yes | Yes |
| Spatial Cut-off | 20 miles | 20 miles | 20 miles | 20 miles | 20 miles | 20 miles | 20 miles |
| Temporal Cut-off | 24 months | 24 months | 24 months | 24 months | 24 months | 24 months | 24 months |

*Note*: The dependent variables have been standardized before estimation. We report the standardized coefficients from the estimations based on Equation (5). The variable *Wildfire* is a binary indicator equal to one if a wildfire occurred within 5 miles of the respondent's village in two years preceding the survey, and zero if the nearest wildfire occurred between 7 miles and 20 miles during the same period. All specifications include county-by-year fixed effects to account for unobserved heterogeneity and national-level temporal trends. Robust standard errors are clustered at the subnational level. Statistical inference of coefficients is based on two-sided t-tests. *** p<0.01, ** p<0.05, * p<0.1.

**Supplementary Table 26** Long-term Effects on Protest Participation

| Dept. Vars = Protest | | | | | | | |
|---|---|---|---|---|---|---|---|
| | (1) | (2) | (3) | (4) | (5) | (6) | (7) |
| Wildfire [0m,12m] | 0.0906 | | | | | | |
| | (0.0767) | | | | | | |
| Wildfire (12m,24m] | | 0.0001 | | | | | |
| | | (0.0793) | | | | | |
| Wildfire (24m,36m] | | | 0.0540 | | | | |
| | | | (0.0831) | | | | |
| Wildfire (36m,48m] | | | | 0.0101 | | | |
| | | | | (0.0692) | | | |
| Wildfire (48m,60m] | | | | | 0.0051 | | |
| | | | | | (0.0760) | | |
| Wildfire (60m,72m] | | | | | | 0.0585 | |
| | | | | | | (0.0724) | |
| Wildfire (72m,84m] | | | | | | | -0.0068 |
| | | | | | | | (0.1080) |
| Observations | 71,053 | 68,650 | 55,807 | 51,156 | 49,637 | 46,543 | 51,208 |
| Country-Year FE | Yes | Yes | Yes | Yes | Yes | Yes | Yes |
| Spatial Cut-off | 20 miles | 20 miles | 20 miles | 20 miles | 20 miles | 20 miles | 20 miles |
| Temporal Cut-off | 24 months | 24 months | 24 months | 24 months | 24 months | 24 months | 24 months |

*Note*: The dependent variables have been standardized before estimation. We report the standardized coefficients from the estimations based on Equation (5). The variable *Wildfire* is a binary indicator equal to one if a wildfire occurred within 5 miles of the respondent's village in two years preceding the survey, and zero if the nearest wildfire occurred between 7 miles and 20 miles during the same period. All specifications include county-by-year fixed effects to account for unobserved heterogeneity and national-level temporal trends. Robust standard errors are clustered at the subnational level. Statistical inference of coefficients is based on two-sided t-tests. *** p<0.01, ** p<0.05, * p<0.1

**Supplementary Table 27** Heterogeneity Analysis: Climate Zones

| Dept. Vars. | Voting | Contacting local govt. | Attending com. meetings | Collective issue-raising | Protest |
|---|---|---|---|---|---|
| | (1) | (2) | (3) | (4) | (5) |
| **Panel A: Humid Areas** | | | | | |
| Wildfire | 0.2710 | 1.3710* | 1.0160 | 1.2170 | 0.8110 |
| | (0.2060) | (0.8140) | (0.9610) | (1.0410) | (0.5280) |
| Observations | 1,811 | 2,337 | 2,337 | 2,337 | 2,337 |
| **Panel B: Areas with Both Seasons** | | | | | |
| Wildfire | 0.0577 | 0.3310** | 0.4250** | 0.1490 | -0.1670 |
| | (0.0532) | (0.1550) | (0.2000) | (0.1590) | (0.1300) |
| Observations | 26,443 | 29,978 | 29,978 | 29,978 | 29,978 |
| **Panel C: Arid Areas** | | | | | |
| Wildfire | 0.0866** | 0.1320 | 0.3830*** | 0.2510** | 0.1300 |
| | (0.0403) | (0.1020) | (0.1180) | (0.1080) | (0.0821) |
| Observations | 41,685 | 50,890 | 50,890 | 50,890 | 50,890 |
| Country-Year FE | Yes | Yes | Yes | Yes | Yes |
| Spatial Cut-off (miles) | 20 | 20 | 20 | 20 | 20 |
| Temporal Cut-off (months) | 24 | 24 | 24 | 24 | 24 |

*Note*: This table reports 2SLS estimation results of the heterogeneous impact of wildfire exposure across the Köppen-Geiger typology [3]. The non-binary dependent variables have been standardized before estimation. We report the standardized coefficients from the estimations based on Equation (5). The variable *Wildfire* is a binary indicator equal to one if a wildfire occurred within 5 miles of the respondent's village in two years preceding the survey, and zero if the nearest wildfire occurred between 7 miles and 20 miles during the same period. All specifications include county-by-year fixed effects to account for unobserved heterogeneity and national-level temporal trends. Robust standard errors are clustered at the subnational level. Statistical inference of coefficients is based on two-sided t-tests. *** $p<0.01$, ** $p<0.05$, * $p<0.1$

**Supplementary Table 28** Heterogeneity Analysis: Climate Zones—OLS

| Dept. Vars. | Voting | Contacting local govt. | Attending com. meetings | Collective issue-raising | Protest |
|---|---|---|---|---|---|
| | (1) | (2) | (3) | (4) | (5) |
| **Panel A: Humid Areas** | | | | | |
| Wildfire | -0.0220 | 0.1460** | 0.1110** | 0.1150** | 0.0789 |
| | (0.0150) | (0.0674) | (0.0492) | (0.0542) | (0.0641) |
| Observations | 1,811 | 2,337 | 2,337 | 2,337 | 2,337 |
| **Panel B: Areas with Both Seasons** | | | | | |
| Wildfire | 0.0152* | 0.0628*** | 0.0862** | 0.0693** | 0.0020 |
| | (0.0080) | (0.0240) | (0.0344) | (0.0275) | (0.0189) |
| Observations | 26,443 | 29,978 | 29,978 | 29,978 | 29,978 |
| **Panel C: Arid Areas** | | | | | |
| Wildfire | -0.0021 | 0.0442*** | 0.0543*** | 0.0337** | 0.0139 |
| | (0.0054) | (0.0170) | (0.0182) | (0.0167) | (0.0157) |
| Observations | 41,685 | 50,890 | 50,890 | 50,890 | 50,890 |
| Country-Year FE | Yes | Yes | Yes | Yes | Yes |
| Spatial Cut-off (miles) | 20 | 20 | 20 | 20 | 20 |
| Temporal Cut-off (months) | 24 | 24 | 24 | 24 | 24 |

*Note*: This table reports the OLS estimation results of heterogeneous impact of wildfire exposure across the Köppen-Geiger typology [3]. The non-binary dependent variables have been standardized before estimation. We report the standardized coefficients from the estimations based on Equation (3). The variable *Wildfire* is a binary indicator equal to one if a wildfire occurred within 5 miles of the respondent's village in two years preceding the survey, and zero if the nearest wildfire occurred between 7 miles and 20 miles during the same period. All specifications include county-by-year fixed effects to account for unobserved heterogeneity and national-level temporal trends. Robust standard errors are clustered at the subnational level. Statistical inference of coefficients is based on two-sided t-tests. *** p<0.01, ** p<0.05, * p<0.1

**Supplementary Table 29** Heterogeneity: Climate Change Literacy

| Dept. Vars. | Voting | Contacting local govt. | Attending com. meetings | Collective issue-raising | Protest |
|---|---|---|---|---|---|
| | (1) | (2) | (3) | (4) | (5) |
| **Panel A: Respondents Living in Regions with High CCL** | | | | | |
| Wildfire | 0.1100*** | 0.2210** | 0.4390*** | 0.2640*** | 0.0394 |
| | (0.0366) | (0.0886) | (0.1190) | (0.1000) | (0.0823) |
| Observations | 43,382 | 50,789 | 50,789 | 50,789 | 50,789 |
| **Panel B: Respondents Living in Regions with Low CCL** | | | | | |
| Wildfire | -0.0516 | 0.0244 | 0.2500* | 0.1090 | 0.0904 |
| | (0.0478) | (0.1180) | (0.1460) | (0.1180) | (0.1010) |
| Observations | 26,558 | 32,417 | 32,417 | 32,417 | 32,417 |
| Country-Year FE | Yes | Yes | Yes | Yes | Yes |
| Spatial Cut-off (miles) | 20 | 20 | 20 | 20 | 20 |
| Temporal Cut-off (months) | 24 | 24 | 24 | 24 | 24 |

This table reports Heterogeneous analysis results with 2SLS estimation. This table separates the sample into two subgroups based on the sample median of the CCL index developed by ref. [4].The non-binary dependent variables have been standardized before estimation. We report the standardized coefficients from the estimations based on Equation (5). The variable *Wildfire* is a binary indicator equal to one if a wildfire occurred within 5 miles of the respondent's village in two years preceding the survey, and zero if the nearest wildfire occurred between 7 miles and 20 miles during the same period. All specifications include county-by-year fixed effects to account for unobserved heterogeneity and national-level temporal trends. Robust standard errors are clustered at the subnational level, with their standardized form reported in parentheses. Statistical inference of coefficients is based on two-sided t-tests. *** p<0.01, ** p<0.05, * p<0.1.

**Supplementary Table 30** Heterogeneity: Climate Change Literacy—OLS

| Dept. Vars. | Voting | Contacting local govt. | Attending com. meetings | Collective issue-raising | Protest |
|---|---|---|---|---|---|
| | (1) | (2) | (3) | (4) | (5) |
| **Panel A: Respondents Living in Regions with High CCL** | | | | | |
| Wildfire | 0.0133** | 0.0276 | 0.0605*** | 0.0464** | 0.0035 |
| | (0.0059) | (0.0181) | (0.0226) | (0.0187) | (0.0138) |
| Observations | 43,382 | 50,789 | 50,789 | 50,789 | 50,789 |
| **Panel B: Respondents Living in Regions with Low CCL** | | | | | |
| Wildfire | -0.0068 | 0.0568*** | 0.0681*** | 0.0489** | 0.0289 |
| | (0.0065) | (0.0179) | (0.0228) | (0.0224) | (0.0207) |
| Observations | 26,558 | 32,417 | 32,417 | 32,417 | 32,417 |
| Country-Year FE | Yes | Yes | Yes | Yes | Yes |
| Spatial Cut-off (miles) | 20 | 20 | 20 | 20 | 20 |
| Temporal Cut-off (months) | 24 | 24 | 24 | 24 | 24 |

This table reports Heterogeneous analysis results with OLS estimation. This table separates the sample into two subgroups based on the sample median of the CCL index developed by ref. [4].The non-binary dependent variables have been standardized before estimation. We report the standardized coefficients from the estimations based on Equation (3). The variable *Wildfire* is a binary indicator equal to one if a wildfire occurred within 5 miles of the respondent's village in two years preceding the survey, and zero if the nearest wildfire occurred between 7 miles and 20 miles during the same period. All specifications include county-by-year fixed effects to account for unobserved heterogeneity and national-level temporal trends. Robust standard errors are clustered at the subnational level. Statistical inference of coefficients is based on two-sided t-tests. *** p<0.01, ** p<0.05, * p<0.1.

**Supplementary Table 31** Effect of Wildfire Exposure on Political Participation by Ethnic Group

| Dept. Vars. | Voting | Contacting local govt. | Attending com. meetings | Collective issue-raising | Protest |
|---|---|---|---|---|---|
| | (1) | (2) | (3) | (4) | (5) |
| **Panel A: Powerful Ethnic Groups** | | | | | |
| Wildfire | 0.0786*** | 0.1850** | 0.4230*** | 0.3030*** | 0.0611 |
| | (0.0280) | (0.0817) | (0.1090) | (0.0894) | (0.0771) |
| Observations | 39,027 | 43,668 | 43,668 | 43,668 | 43,668 |
| **Panel B: Powerless Ethnic Groups** | | | | | |
| Wildfire | -0.1390 | -0.0777 | -0.0192 | -0.4100 | -0.0151 |
| | (0.2100) | (0.3940) | (0.2780) | (0.4350) | (0.2590) |
| Observations | 9,555 | 11,691 | 11,691 | 11,691 | 11,691 |
| Country-Year FE | Yes | Yes | Yes | Yes | Yes |
| Spatial Cut-off (miles) | 20 | 20 | 20 | 20 | 20 |
| Temporal Cut-off (months) | 24 | 24 | 24 | 24 | 24 |

*Notes*: This table reports the 2SLS estimation results of heterogeneous effects of wildfire exposure on political participation by ethnic group political status, as defined by the GeoEPR-ETH Version 2.0 dataset (GeoEPR-ETH V2) [5]. An ethnic group is defined as "powerful" if it's described as "dominant", "senior partner" or "junior partner" in the dataset, and is defined as "powerless" if it's described as "irrelevant", "powerless" or "discriminated". The non-binary dependent variables have been standardized before estimation. We report the standardized coefficients from the estimations based on Equation (5). The variable *Wildfire* is a binary indicator equal to one if a wildfire occurred within 5 miles of the respondent's village in two years preceding the survey, and zero if the nearest wildfire occurred between 7 miles and 20 miles during the same period. All specifications include county-by-year fixed effects to account for unobserved heterogeneity and national-level temporal trends. Robust standard errors are clustered at the subnational level. Statistical inference of coefficients is based on two-sided t-tests. *** p<0.01, ** p<0.05, * p<0.1

**Supplementary Table 32** Effect of Wildfire Exposure on Political Participation by Ethnic Group—OLS

| Dept. Vars. | Voting | Contacting local govt. | Attending com. meetings | Collective issue-raising | Protest |
|---|---|---|---|---|---|
| | (1) | (2) | (3) | (4) | (5) |
| **Panel A: Powerful Ethnic Groups** | | | | | |
| Wildfire | 0.0020 | 0.0433** | 0.0707*** | 0.0551*** | 0.0176 |
| | (0.0057) | (0.0189) | (0.0240) | (0.0206) | (0.0161) |
| Observations | 39,027 | 43,668 | 43,668 | 43,668 | 43,668 |
| **Panel B: Powerless Ethnic Groups** | | | | | |
| Wildfire | -0.0070 | 0.0403 | 0.0087 | 0.0011 | -0.0295 |
| | (0.0113) | (0.0386) | (0.0333) | (0.0316) | (0.0251) |
| Observations | 9,555 | 11,691 | 11,691 | 11,691 | 11,691 |
| Country-Year FE | Yes | Yes | Yes | Yes | Yes |
| Spatial Cut-off (miles) | 20 | 20 | 20 | 20 | 20 |
| Temporal Cut-off (months) | 24 | 24 | 24 | 24 | 24 |

*Notes*: This table reports the OLS estimation results of heterogeneous effects of wildfire exposure on political participation by ethnic group political status, as defined by the GeoEPR-ETH Version 2.0 dataset (GeoEPR-ETH V2) [5]. An ethnic group is defined as ”powerful” if it’s described as ”dominant”, ”senior partner” or ”junior partner” in the dataset, and is defined as ”powerless” if it’s described as ”irrelevant”, ”powerless” or ”discriminated”. The non-binary dependent variables have been standardized before estimation. We report the standardized coefficients from the estimations based on Equation (3). The variable *Wildfire* is a binary indicator equal to one if a wildfire occurred within 5 miles of the respondent’s village in two years preceding the survey, and zero if the nearest wildfire occurred between 7 miles and 20 miles during the same period. All specifications include county-by-year fixed effects to account for unobserved heterogeneity and national-level temporal trends. Robust standard errors are clustered at the subnational level. Statistical inference of coefficients is based on two-sided t-tests. *** $p<0.01$, ** $p<0.05$, * $p<0.1$

**Supplementary Table 33** Heterogeneity: Proximity to Ethnic Group Border

| Dept. Vars. | Voting | Contacting local govt. | Attending com. meetings | Collective issue-raising | Protest |
|---|---|---|---|---|---|
| | (1) | (2) | (3) | (4) | (5) |
| **Panel A: Respondents within Ethnic Border Areas** | | | | | |
| Wildfire | 0.0518 | 0.3160*** | 0.5840*** | 0.3140*** | 0.2370*** |
| | (0.0324) | (0.0720) | (0.0995) | (0.0803) | (0.0890) |
| Observations | 17,715 | 22,214 | 22,214 | 22,214 | 22,214 |
| **Panel B: Respondents out of Ethnic Border Areas** | | | | | |
| Wildfire | 0.1040** | -0.0036 | 0.1990 | 0.0766 | -0.1510 |
| | (0.0460) | (0.1270) | (0.1490) | (0.1320) | (0.1070) |
| Observations | 52,225 | 60,992 | 60,992 | 60,992 | 60,992 |
| Country-Year FE | Yes | Yes | Yes | Yes | Yes |
| Spatial Cut-off (miles) | 20 | 20 | 20 | 20 | 20 |
| Temporal Cut-off (months) | 24 | 24 | 24 | 24 | 24 |

*Notes*: This table reports 2SLS estimation results of heterogeneous analysis results considering different distances to ethnic group border. Respondents within ethnic border areas are defined as those whose villages are located within five miles of the nearest ethnic group boundary, while respondents outside ethnic border areas are those residing beyond this five-mile threshold. Non-binary dependent variables have been standardized before estimation. We report the standardized coefficients from the estimations based on Equation (5). The variable *Wildfire* is a binary indicator equal to one if a wildfire occurred within 5 miles of the respondent's village in two years preceding the survey, and zero if the nearest wildfire occurred between 7 miles and 20 miles during the same period. All specifications include county-by-year fixed effects to account for unobserved heterogeneity and national-level temporal trends. Robust standard errors are clustered at the subnational level. Statistical inference of coefficients is based on two-sided t-tests. *** p<0.01, ** p<0.05, * p<0.1.

**Supplementary Table 34** Heterogeneity: Proximity to Ethnic Group Border—OLS

| Dept. Vars. | Voting | Contacting local govt. | Attending com. meetings | Collective issue-raising | Protest |
|---|---|---|---|---|---|
| | (1) | (2) | (3) | (4) | (5) |
| **Panel A: Respondents within Ethnic Border Areas** | | | | | |
| Wildfire | 0.0068 | 0.0748*** | 0.1200*** | 0.0938*** | 0.0230 |
| | (0.0088) | (0.0245) | (0.0331) | (0.0268) | (0.0216) |
| Observations | 17,715 | 22,214 | 22,214 | 22,214 | 22,214 |
| **Panel B: Respondents out of Ethnic Border Areas** | | | | | |
| Wildfire | 0.0028 | 0.0342** | 0.0504*** | 0.0364** | 0.0112 |
| | (0.0053) | (0.0151) | (0.0180) | (0.0168) | (0.0143) |
| Observations | 52,225 | 60,992 | 60,992 | 60,992 | 60,992 |
| Country-Year FE | Yes | Yes | Yes | Yes | Yes |
| Spatial Cut-off (miles) | 20 | 20 | 20 | 20 | 20 |
| Temporal Cut-off (months) | 24 | 24 | 24 | 24 | 24 |

*Notes*: This table reports OLS estimation results of heterogeneous analysis results considering different distances to ethnic group border. Respondents within ethnic border areas are defined as those whose villages are located within five miles of the nearest ethnic group boundary, while respondents outside ethnic border areas are those residing beyond this five-mile threshold. Non-binary dependent variables have been standardized before estimation. We report the standardized coefficients from the estimations based on Equation (3). The variable *Wildfire* is a binary indicator equal to one if a wildfire occurred within 5 miles of the respondent's village in two years preceding the survey, and zero if the nearest wildfire occurred between 7 miles and 20 miles during the same period. All specifications include county-by-year fixed effects to account for unobserved heterogeneity and national-level temporal trends. Robust standard errors are clustered at the subnational level. Statistical inference of coefficients is based on two-sided t-tests. *** p<0.01, ** p<0.05, * p<0.1.

**Supplementary Table 35** Heterogeneity: The Presence of A Tarred Road

| Dept. Vars. | Voting | Contacting local govt. | Attending com. meetings | Collective issue-raising | Protest |
|---|---|---|---|---|---|
| | (1) | (2) | (3) | (4) | (5) |
| **Panel A: With A Tarred Road Nearby** | | | | | |
| Wildfire | 0.1330*** | 0.2620*** | 0.4490*** | 0.2390** | 0.0435 |
| | (0.0359) | (0.0913) | (0.1170) | (0.1020) | (0.0916) |
| Observations | 29,535 | 34,645 | 34,645 | 34,645 | 34,645 |
| **Panel B: Without A Tarred Road Nearby** | | | | | |
| Wildfire | 0.0266 | 0.0794 | 0.3660*** | 0.2430** | 0.1000 |
| | (0.0361) | (0.1170) | (0.1140) | (0.1110) | (0.0984) |
| Observations | 40,404 | 48,559 | 48,559 | 48,559 | 48,559 |
| Country-Year FE | Yes | Yes | Yes | Yes | Yes |
| Spatial Cut-off (miles) | 20 | 20 | 20 | 20 | 20 |
| Temporal Cut-off (months) | 24 | 24 | 24 | 24 | 24 |

*Note*: This table reports 2SLS estimation results of heterogeneous analysis results by dividing the sample into two subgroups based on self-reported tarred road accessibility in the Afrobarometer survey. The non-binary dependent variables have been standardized before estimation. We report the standardized coefficients from the estimations based on Equation (5). The variable *Wildfire* is a binary indicator equal to one if a wildfire occurred within 5 miles of the respondent's village in two years preceding the survey, and zero if the nearest wildfire occurred between 7 miles and 20 miles during the same period. All specifications include county-by-year fixed effects to account for unobserved heterogeneity and national-level temporal trends. Robust standard errors are clustered at the subnational level. Statistical inference of coefficients is based on two-sided t-tests. *** $p<0.01$, ** $p<0.05$, * $p<0.1$.

**Supplementary Table 36** Heterogeneity: The Presence of A Tarred Road–OLS

| Dept. Vars. | Voting | Contacting local govt. | Attending com. meetings | Collective issue-raising | Protest |
|---|---|---|---|---|---|
| | (1) | (2) | (3) | (4) | (5) |
| | | **Panel A: With A Tarred Road Nearby** | | | |
| Wildfire | 0.0134** | 0.0511*** | 0.0725*** | 0.0664*** | 0.0396** |
| | (0.0066) | (0.0171) | (0.0237) | (0.0210) | (0.0176) |
| Observations | 29,535 | 34,645 | 34,645 | 34,645 | 34,645 |
| | | **Panel B: Without A Tarred Road Nearby** | | | |
| Wildfire | -0.0007 | 0.0407** | 0.0688*** | 0.0377** | -0.0078 |
| | (0.0053) | (0.0167) | (0.0181) | (0.0166) | (0.0135) |
| Observations | 40,404 | 48,559 | 48,559 | 48,559 | 48,559 |
| Country-Year FE | Yes | Yes | Yes | Yes | Yes |
| Spatial Cut-off (miles) | 20 | 20 | 20 | 20 | 20 |
| Temporal Cut-off (months) | 24 | 24 | 24 | 24 | 24 |

*Note*: This table reports OLS estimation results of heterogeneous analysis results by dividing the sample into two subgroups based on self-reported tarred road accessibility in the Afrobarometer survey. The non-binary dependent variables have been standardized before estimation. We report the standardized coefficients from the estimations based on Equation (3). The variable *Wildfire* is a binary indicator equal to one if a wildfire occurred within 5 miles of the respondent's village in two years preceding the survey, and zero if the nearest wildfire occurred between 7 miles and 20 miles during the same period. All specifications include county-by-year fixed effects to account for unobserved heterogeneity and national-level temporal trends. Robust standard errors are clustered at the subnational level. Statistical inference of coefficients is based on two-sided t-tests. *** p<0.01, ** p<0.05, * p<0.1.

**Supplementary Table 37** Heterogeneity on Information Accessibility: TV Ownership

| Dept. Vars. | Voting | Contacting local govt. | Attending com. meetings | Collective issue-raising | Protest |
|---|---|---|---|---|---|
| | (1) | (2) | (3) | (4) | (5) |
| **Panel A: Owning a TV** | | | | | |
| Wildfire | 0.0604* | 0.3030*** | 0.4400*** | 0.3490*** | 0.0690 |
| | (0.0315) | (0.0824) | (0.1230) | (0.1020) | (0.0900) |
| Observations | 27,581 | 32,371 | 32,371 | 32,371 | 32,371 |
| **Panel B: Without a TV** | | | | | |
| Wildfire | 0.0915* | 0.0957 | 0.3060*** | 0.1610 | 0.1380 |
| | (0.0507) | (0.1190) | (0.1120) | (0.1050) | (0.0975) |
| Observations | 42,214 | 50,677 | 50,677 | 50,677 | 50,677 |
| Country-Year FE | Yes | Yes | Yes | Yes | Yes |
| Spatial Cut-off (miles) | 20 | 20 | 20 | 20 | 20 |
| Temporal Cut-off (months) | 24 | 24 | 24 | 24 | 24 |

*Note*: This table reports 2SLS estimation results of heterogeneous analysis results by dividing The non-binary dependent variables have been standardized before estimation. We report the standardized coefficients from the estimations based on Equation (5). The variable *Wildfire* is a binary indicator equal to one if a wildfire occurred within 5 miles of the respondent's village in two years preceding the survey, and zero if the nearest wildfire occurred between 7 miles and 20 miles during the same period. All specifications include county-by-year fixed effects to account for unobserved heterogeneity and national-level temporal trends. Robust standard errors are clustered at the subnational level. Statistical inference of coefficients is based on two-sided t-tests. *** p<0.01, ** p<0.05, * p<0.1.

**Supplementary Table 38** Heterogeneity on Information Accessibility: TV Ownership–OLS

| Dept. Vars. | Voting | Contacting local govt. | Attending com. meetings | Collective issue-raising | Protest |
|---|---|---|---|---|---|
| | (1) | (2) | (3) | (4) | (5) |
| **Panel A: Owning a TV** | | | | | |
| Wildfire | 0.0164** | 0.0830*** | 0.0924*** | 0.0977*** | 0.0553*** |
| | (0.0069) | (0.0181) | (0.0253) | (0.0233) | (0.0185) |
| Observations | 27,581 | 32,371 | 32,371 | 32,371 | 32,371 |
| **Panel B: Without a TV** | | | | | |
| Wildfire | -0.0023 | 0.0283* | 0.0504*** | 0.0236 | -0.0017 |
| | (0.0052) | (0.0155) | (0.0168) | (0.0147) | (0.0126) |
| Observations | 42,214 | 50,677 | 50,677 | 50,677 | 50,677 |
| Country-Year FE | Yes | Yes | Yes | Yes | Yes |
| Spatial Cut-off (miles) | 20 | 20 | 20 | 20 | 20 |
| Temporal Cut-off (months) | 24 | 24 | 24 | 24 | 24 |

*Note*: This table reports OLS estimation results of heterogeneous analysis results by dividing the non-binary dependent variables have been standardized before estimation. We report the standardized coefficients from the estimations based on Equation (3). The variable *Wildfire* is a binary indicator equal to one if a wildfire occurred within 5 miles of the respondent's village in two years preceding the survey, and zero if the nearest wildfire occurred between 7 miles and 20 miles during the same period. All specifications include county-by-year fixed effects to account for unobserved heterogeneity and national-level temporal trends. Robust standard errors are clustered at the subnational level. Statistical inference of coefficients is based on two-sided t-tests. *** p<0.01, ** p<0.05, * p<0.1.

**Supplementary Table 39** Heterogeneity on Information Accessibility: Radio Ownership

| Dept. Vars. | Voting | Contacting local govt. | Attending com. meetings | Collective issue-raising | Protest |
|---|---|---|---|---|---|
| | (1) | (2) | (3) | (4) | (5) |
| **Panel A: With Own Radio** | | | | | |
| Wildfire | 0.0724** | 0.2050** | 0.4380*** | 0.2470*** | 0.0876 |
| | (0.0296) | (0.0827) | (0.1060) | (0.0922) | (0.0761) |
| Observations | 49,786 | 58,895 | 58,895 | 58,895 | 58,895 |
| **Panel B: Without Own Radio** | | | | | |
| Wildfire | 0.0788 | 0.0771 | 0.2500** | 0.2030* | -0.0661 |
| | (0.0553) | (0.1180) | (0.1210) | (0.1160) | (0.0987) |
| Observations | 20,081 | 24,218 | 24,218 | 24,218 | 24,218 |
| Country-Year FE | Yes | Yes | Yes | Yes | Yes |
| Spatial Cut-off (miles) | 20 | 20 | 20 | 20 | 20 |
| Temporal Cut-off (months) | 24 | 24 | 24 | 24 | 24 |

*Note*: This table reports 2SLS estimation results of heterogeneous analysis results by dividing the sample into two subgroups based on self-reported TV ownership. Non-binary dependent variables have been standardized before estimation. We report the standardized coefficients from the estimations based on Equation (5). The variable *Wildfire* is a binary indicator equal to one if a wildfire occurred within 5 miles of the respondent's village in two years preceding the survey, and zero if the nearest wildfire occurred between 7 miles and 20 miles during the same period. All specifications include county-by-year fixed effects to account for unobserved heterogeneity and national-level temporal trends. Robust standard errors are clustered at the subnational level. Statistical inference of coefficients is based on two-sided t-tests. *** p<0.01, ** p<0.05, * p<0.1

**Supplementary Table 40** Heterogeneity on Information Accessibility: Radio Ownership–OLS

| Dept. Vars. | Voting | Contacting local govt. | Attending com. meetings | Collective issue-raising | Protest |
|---|---|---|---|---|---|
| | (1) | (2) | (3) | (4) | (5) |
| | | | **Panel A: With Own Radio** | | |
| Wildfire | 0.0064 | 0.0574*** | 0.0813*** | 0.0569*** | 0.0234 |
| | (0.0051) | (0.0165) | (0.0200) | (0.0173) | (0.0142) |
| Observations | 49,786 | 58,895 | 58,895 | 58,895 | 58,895 |
| | | | **Panel B: Without Own Radio** | | |
| Wildfire | 0.0018 | 0.0287* | 0.0425** | 0.0428** | -0.0099 |
| | (0.0073) | (0.0162) | (0.0215) | (0.0217) | (0.0167) |
| Observations | 20,081 | 24,218 | 24,218 | 24,218 | 24,218 |
| Country-Year FE | Yes | Yes | Yes | Yes | Yes |
| Spatial Cut-off (miles) | 20 | 20 | 20 | 20 | 20 |
| Temporal Cut-off (months) | 24 | 24 | 24 | 24 | 24 |

*Note*: This table reports OLS estimation results of heterogeneous analysis results by dividing the sample into two subgroups based on self-reported TV ownership. Non-binary dependent variables have been standardized before estimation. We report the standardized coefficients from the estimations based on Equation (3). The variable *Wildfire* is a binary indicator equal to one if a wildfire occurred within 5 miles of the respondent's village in two years preceding the survey, and zero if the nearest wildfire occurred between 7 miles and 20 miles during the same period. All specifications include county-by-year fixed effects to account for unobserved heterogeneity and national-level temporal trends. Robust standard errors are clustered at the subnational level. Statistical inference of coefficients is based on two-sided t-tests. *** p<0.01, ** p<0.05, * p<0.1

**Supplementary Table 41** Heterogeneity: Gender

| Dept. Vars. | Voting | Contacting local govt. | Attending com. meetings | Collective issue-raising | Protest |
|---|---|---|---|---|---|
| | (1) | (2) | (3) | (4) | (5) |
| **Panel A: Female** | | | | | |
| Wildfire | 0.0990*** | 0.0975 | 0.3500*** | 0.1510* | 0.0475 |
| | (0.0333) | (0.0757) | (0.0972) | (0.0899) | (0.0666) |
| Observations | 34,866 | 41,543 | 41,543 | 41,543 | 41,543 |
| **Panel B: Male** | | | | | |
| Wildfire | 0.0472 | 0.1950** | 0.3940*** | 0.2590*** | 0.0221 |
| | (0.0319) | (0.0990) | (0.1120) | (0.0892) | (0.0853) |
| Observations | 35,074 | 41,663 | 41,663 | 41,663 | 41,663 |
| Country-Year FE | Yes | Yes | Yes | Yes | Yes |
| Spatial Cut-off (miles) | 20 | 20 | 20 | 20 | 20 |
| Temporal Cut-off (months) | 24 | 24 | 24 | 24 | 24 |

*Note*: This table reports 2SLS estimation results of heterogeneous analysis. We separate the sample into two subgroups based on respondents' gender. The non-binary dependent variables have been standardized before estimation. We report the standardized coefficients from the estimations based on Equation (5). The variable *Wildfire* is a binary indicator equal to one if a wildfire occurred within 5 miles of the respondent's village in two years preceding the survey, and zero if the nearest wildfire occurred between 7 miles and 20 miles during the same period. All specifications include county-by-year fixed effects to account for unobserved heterogeneity and national-level temporal trends. Robust standard errors are clustered at the subnational level. Statistical inference of coefficients is based on two-sided t-tests. *** p<0.01, ** p<0.05, * p<0.1

**Supplementary Table 42** Heterogeneity: Gender—OLS

| Dept. Vars. | Voting | Contacting local govt. | Attending com. meetings | Collective issue-raising | Protest |
|---|---|---|---|---|---|
| | (1) | (2) | (3) | (4) | (5) |
| **Panel A: Female** | | | | | |
| Wildfire | 0.0065 | 0.0411*** | 0.0572*** | 0.0505*** | -0.0013 |
| | (0.0061) | (0.0138) | (0.0186) | (0.0167) | (0.0131) |
| Observations | 34,866 | 41,543 | 41,543 | 41,543 | 41,543 |
| **Panel B: Male** | | | | | |
| Wildfire | 0.0025 | 0.0454** | 0.0768*** | 0.0471*** | 0.0271* |
| | (0.0052) | (0.0184) | (0.0206) | (0.0174) | (0.0158) |
| Observations | 35,074 | 41,663 | 41,663 | 41,663 | 41,663 |
| Country-Year FE | Yes | Yes | Yes | Yes | Yes |
| Spatial Cut-off (miles) | 20 | 20 | 20 | 20 | 20 |
| Temporal Cut-off (months) | 24 | 24 | 24 | 24 | 24 |

*Note*: This table reports OLS estimation results of heterogeneous analysis. We separate the sample into two subgroups based on respondents' gender. The non-binary dependent variables have been standardized before estimation. We report the standardized coefficients from the estimations based on Equation (3). The variable *Wildfire* is a binary indicator equal to one if a wildfire occurred within 5 miles of the respondent's village in two years preceding the survey, and zero if the nearest wildfire occurred between 7 miles and 20 miles during the same period. All specifications include county-by-year fixed effects to account for unobserved heterogeneity and national-level temporal trends. Robust standard errors are clustered at the subnational level. Statistical inference of coefficients is based on two-sided t-tests. *** p<0.01, ** p<0.05, * p<0.1

**Supplementary Table 43** Heterogeneity: Education

| Dept. Vars. | Voting | Contacting local govt. | Attending com. meetings | Collective issue-raising | Protest |
|---|---|---|---|---|---|
| | (1) | (2) | (3) | (4) | (5) |
| **Panel A: Having Attended Secondary School** | | | | | |
| Wildfire | 0.0797** | 0.2880*** | 0.4180*** | 0.2570*** | 0.0864 |
| | (0.0326) | (0.0848) | (0.1190) | (0.0951) | (0.0909) |
| Observations | 32,517 | 40,665 | 40,665 | 40,665 | 40,665 |
| **Panel B: Without Secondary School Experiences** | | | | | |
| Wildfire | 0.0610 | 0.1030 | 0.3790*** | 0.2330** | 0.1110 |
| | (0.0383) | (0.1060) | (0.1210) | (0.1150) | (0.0879) |
| Observations | 37,423 | 42,541 | 42,541 | 42,541 | 42,541 |
| Country-Year FE | Yes | Yes | Yes | Yes | Yes |
| Spatial Cut-off (miles) | 20 | 20 | 20 | 20 | 20 |
| Temporal Cut-off (months) | 24 | 24 | 24 | 24 | 24 |

*Note*: This table reports 2SLS estimation results of heterogeneous analysis. We separate the sample into two subgroups based on respondents' gender. The non-binary dependent variables have been standardized before estimation. We report the standardized coefficients from the estimations based on Equation (5). The variable *Wildfire* is a binary indicator equal to one if a wildfire occurred within 5 miles of the respondent's village in two years preceding the survey, and zero if the nearest wildfire occurred between 7 miles and 20 miles during the same period. All specifications include county-by-year fixed effects to account for unobserved heterogeneity and national-level temporal trends. Robust standard errors are clustered at the subnational level. Statistical inference of coefficients is based on two-sided t-tests. *** p<0.01, ** p<0.05, * p<0.1

**Supplementary Table 44** Heterogeneity: Education—OLS

| Dept. Vars. | Voting | Contacting local govt. | Attending com. meetings | Collective issue-raising | Protest |
|---|---|---|---|---|---|
| | (1) | (2) | (3) | (4) | (5) |
| **Panel A: Having Attended Secondary School** | | | | | |
| Wildfire | 0.0147** | 0.0508*** | 0.0898*** | 0.0690*** | 0.0265 |
| | (0.0062) | (0.0185) | (0.0227) | (0.0196) | (0.0161) |
| Observations | 32,517 | 40,665 | 40,665 | 40,665 | 40,665 |
| **Panel B: Without Secondary School Experiences** | | | | | |
| Wildfire | -0.0053 | 0.0484*** | 0.0529*** | 0.0417** | 0.0132 |
| | (0.0053) | (0.0164) | (0.0177) | (0.0175) | (0.0142) |
| Observations | 37,423 | 42,541 | 42,541 | 42,541 | 42,541 |
| Country-Year FE | Yes | Yes | Yes | Yes | Yes |
| Spatial Cut-off (miles) | 20 | 20 | 20 | 20 | 20 |
| Temporal Cut-off (months) | 24 | 24 | 24 | 24 | 24 |

*Note*: This table reports OLS estimation results of heterogeneous analysis. We separate the sample into two subgroups based on respondents' gender. The non-binary dependent variables have been standardized before estimation. We report the standardized coefficients from the estimations based on Equation (3). The variable *Wildfire* is a binary indicator equal to one if a wildfire occurred within 5 miles of the respondent's village in two years preceding the survey, and zero if the nearest wildfire occurred between 7 miles and 20 miles during the same period. All specifications include county-by-year fixed effects to account for unobserved heterogeneity and national-level temporal trends. Robust standard errors are clustered at the subnational level. Statistical inference of coefficients is based on two-sided t-tests. *** p<0.01, ** p<0.05, * p<0.1

**Supplementary Table 45** Heterogeneity: Urban Residency

| Dept. Vars. | Voting | Contacting local govt. | Attending com. meetings | Collective issue-raising | Protest |
|---|---|---|---|---|---|
| | (1) | (2) | (3) | (4) | (5) |
| **Panel A: Respondents Living in Urban Areas** | | | | | |
| Wildfire | 0.0789*** | 0.2140*** | 0.4600*** | 0.3060*** | 0.1000 |
| | (0.0261) | (0.0681) | (0.1030) | (0.0875) | (0.0828) |
| Observations | 25,655 | 31,823 | 31,823 | 31,823 | 31,823 |
| **Panel B: Respondents Living in Non-Urban Areas** | | | | | |
| Wildfire | 0.0171 | 0.0154 | 0.0568 | -0.0593 | -0.0680 |
| | (0.0610) | (0.1560) | (0.1280) | (0.1400) | (0.1300) |
| Observations | 44,285 | 51,382 | 51,382 | 51,382 | 51,382 |
| Country-Year FE | Yes | Yes | Yes | Yes | Yes |
| Spatial Cut-off (miles) | 20 | 20 | 20 | 20 | 20 |
| Temporal Cut-off (months) | 24 | 24 | 24 | 24 | 24 |

*Note*: This table reports 2SLS estimation results of heterogeneous analysis. We separate the sample into two subgroups based on respondents' place of residence: urban versus non-urban areas. The non-binary dependent variables have been standardized before estimation. We report the standardized coefficients from the estimations based on Equation (5). The variable *Wildfire* is a binary indicator equal to one if a wildfire occurred within 5 miles of the respondent's village in two years preceding the survey, and zero if the nearest wildfire occurred between 7 miles and 20 miles during the same period. All specifications include county-by-year fixed effects to account for unobserved heterogeneity and national-level temporal trends. Robust standard errors are clustered at the subnational level. Statistical inference of coefficients is based on two-sided t-tests. *** p<0.01, ** p<0.05, * p<0.1.

**Supplementary Table 46** Heterogeneity: Urban Residency—OLS

| Dept. Vars. | Voting | Contacting local govt. | Attending com. meetings | Collective issue-raising | Protest |
|---|---|---|---|---|---|
| | (1) | (2) | (3) | (4) | (5) |
| **Panel A: Respondents Living in Urban Areas** | | | | | |
| Wildfire | 0.0182*** | 0.0645*** | 0.1000*** | 0.0939*** | 0.0427** |
| | (0.0063) | (0.0173) | (0.0253) | (0.0233) | (0.0182) |
| Observations | 25,655 | 31,823 | 31,823 | 31,823 | 31,823 |
| **Panel B: Respondents Living in Non-Urban Areas** | | | | | |
| Wildfire | -0.0047 | 0.0181 | 0.0284* | 0.0092 | -0.0048 |
| | (0.0050) | (0.0154) | (0.0146) | (0.0143) | (0.0138) |
| Observations | 44,285 | 51,382 | 51,382 | 51,382 | 51,382 |
| Country-Year FE | Yes | Yes | Yes | Yes | Yes |
| Spatial Cut-off (miles) | 20 | 20 | 20 | 20 | 20 |
| Temporal Cut-off (months) | 24 | 24 | 24 | 24 | 24 |

*Note*: This table reports OLS estimation results of heterogeneous analysis. We separate the sample into two subgroups based on respondents' place of residence: urban versus non-urban areas. The non-binary dependent variables have been standardized before estimation. We report the standardized coefficients from the estimations based on Equation (3). The variable *Wildfire* is a binary indicator equal to one if a wildfire occurred within 5 miles of the respondent's village in two years preceding the survey, and zero if the nearest wildfire occurred between 7 miles and 20 miles during the same period. All specifications include county-by-year fixed effects to account for unobserved heterogeneity and national-level temporal trends. Robust standard errors are clustered at the subnational level. Statistical inference of coefficients is based on two-sided t-tests. *** p<0.01, ** p<0.05, * p<0.1.

**Supplementary Table 47** Heterogeneity: Occupation

| Dept. Vars. | Voting | Contacting local govt. | Attending com. meetings | Collective issue-raising | Protest |
|---|---|---|---|---|---|
| | (1) | (2) | (3) | (4) | (5) |
| **Panel A: Respondents Working in Agricultural Sector** | | | | | |
| Wildfire | 0.0029 | 0.1360 | 0.4980*** | 0.4760*** | 0.2900 |
| | (0.1370) | (0.1640) | (0.1730) | (0.1680) | (0.1760) |
| Observations | 4,665 | 7,258 | 7,258 | 7,258 | 7,258 |
| **Panel B: Respondents Working in Non-agricultural Sector** | | | | | |
| Wildfire | 0.0830 | 0.2000** | 0.5130*** | 0.3570*** | 0.0259 |
| | (0.0555) | (0.0979) | (0.1030) | (0.0986) | (0.1170) |
| Observations | 12,911 | 19,581 | 19,581 | 19,581 | 19,581 |
| Country-Year FE | Yes | Yes | Yes | Yes | Yes |
| Spatial Cut-off (miles) | 20 | 20 | 20 | 20 | 20 |
| Temporal Cut-off (months) | 24 | 24 | 24 | 24 | 24 |

*Note*: This table reports 2SLS estimation results of heterogeneous analysis. We separate the sample into two subgroups based on respondents' occupations: working in agricultural sectors versus non-agricultural sectors. Notice that only samples in survey round 3 and 6 are included in this analysis since this question is only included in these two survey rounds. The non-binary dependent variables have been standardized before estimation. We report the standardized coefficients from the estimations based on Equation (5). The variable *Wildfire* is a binary indicator equal to one if a wildfire occurred within 5 miles of the respondent's village in two years preceding the survey, and zero if the nearest wildfire occurred between 7 miles and 20 miles during the same period. All specifications include county-by-year fixed effects to account for unobserved heterogeneity and national-level temporal trends. Robust standard errors are clustered at the subnational level. Statistical inference of coefficients is based on two-sided t-tests. *** p<0.01, ** p<0.05, * p<0.1.

**Supplementary Table 48** Heterogeneity: Occupation—OLS

| Dept. Vars. | Voting | Contacting local govt. | Attending com. meetings | Collective issue-raising | Protest |
|---|---|---|---|---|---|
| | (1) | (2) | (3) | (4) | (5) |
| **Panel A: Respondents Working in Agricultural Sector** | | | | | |
| Wildfire | -0.0336* | 0.0296 | 0.0473 | 0.0302 | -0.0142 |
| | (0.0179) | (0.0302) | (0.0323) | (0.0362) | (0.0272) |
| Observations | 4,665 | 7,258 | 7,258 | 7,258 | 7,258 |
| **Panel B: Respondents Working in Non-agricultural Sector** | | | | | |
| Wildfire | -0.0029 | 0.0602*** | 0.0930*** | 0.0631*** | 0.0306 |
| | (0.0083) | (0.0215) | (0.0255) | (0.0212) | (0.0247) |
| Observations | 12,911 | 19,581 | 19,581 | 19,581 | 19,581 |
| Country-Year FE | Yes | Yes | Yes | Yes | Yes |
| Spatial Cut-off (miles) | 20 | 20 | 20 | 20 | 20 |
| Temporal Cut-off (months) | 24 | 24 | 24 | 24 | 24 |

*Note*: This table reports OLS estimation results of heterogeneous analysis. We separate the sample into two subgroups based on respondents' occupations: working in agricultural sectors versus non-agricultural sectors. Notice that only samples in survey round 3 and 6 are included in this analysis since this question is only included in these two survey rounds. The non-binary dependent variables have been standardized before estimation. We report the standardized coefficients from the estimations based on Equation (3). The variable *Wildfire* is a binary indicator equal to one if a wildfire occurred within 5 miles of the respondent's village in two years preceding the survey, and zero if the nearest wildfire occurred between 7 miles and 20 miles during the same period. All specifications include county-by-year fixed effects to account for unobserved heterogeneity and national-level temporal trends. Robust standard errors are clustered at the subnational level. Statistical inference of coefficients is based on two-sided t-tests. *** p<0.01, ** p<0.05, * p<0.1.

**Supplementary Table 49** Heterogeneity: Shelter Type

| Dept. Vars. | Voting | Contacting local govt. | Attending com. meetings | Collective issue-raising | Protest |
|---|---|---|---|---|---|
| | (1) | (2) | (3) | (4) | (5) |
| **Panel A: With Formal Shelter** | | | | | |
| Wildfire | 0.0980*** | 0.2860*** | 0.6060*** | 0.4770*** | 0.0550 |
| | (0.0338) | (0.0858) | (0.1320) | (0.1020) | (0.0815) |
| Observations | 33,068 | 34,987 | 34,987 | 34,987 | 34,987 |
| **Panel B: With Informal Shelter** | | | | | |
| Wildfire | 0.0155 | -0.1250 | -0.0981 | -0.1410 | 0.0790 |
| | (0.0821) | (0.2600) | (0.2050) | (0.2160) | (0.1780) |
| Observations | 17,265 | 16,903 | 16,903 | 16,903 | 16,903 |
| Country-Year FE | Yes | Yes | Yes | Yes | Yes |
| Spatial Cut-off (miles) | 20 | 20 | 20 | 20 | 20 |
| Temporal Cut-off (months) | 24 | 24 | 24 | 24 | 24 |

*Note*: This table reports 2SLS estimation results of heterogeneous analysis. We separate the sample into two subgroups based on respondents' shelter types. The non-binary dependent variables have been standardized before estimation. We report the standardized coefficients from the estimations based on Equation (5). The variable *Wildfire* is a binary indicator equal to one if a wildfire occurred within 5 miles of the respondent's village in two years preceding the survey, and zero if the nearest wildfire occurred between 7 miles and 20 miles during the same period. All specifications include county-by-year fixed effects to account for unobserved heterogeneity and national-level temporal trends. Robust standard errors are clustered at the subnational level. Statistical inference of coefficients is based on two-sided t-tests. *** p<0.01, ** p<0.05, * p<0.1.

**Supplementary Table 50** Heterogeneity: Shelter Type—OLS

| Dept. Vars. | Voting | Contacting local govt. | Attending com. meetings | Collective issue-raising | Protest |
|---|---|---|---|---|---|
| | (1) | (2) | (3) | (4) | (5) |
| **Panel A: With Formal Shelter** | | | | | |
| Wildfire | 0.0138** | 0.0463** | 0.0714** | 0.0808*** | 0.0223 |
| | (0.0070) | (0.0203) | (0.0277) | (0.0231) | (0.0172) |
| Observations | 33,068 | 34,987 | 34,987 | 34,987 | 34,987 |
| **Panel B: With Informal Shelter** | | | | | |
| Wildfire | -0.0115* | 0.0387 | 0.0542** | 0.0423* | -0.0095 |
| | (0.0068) | (0.0277) | (0.0244) | (0.0256) | (0.0206) |
| Observations | 17,265 | 16,903 | 16,903 | 16,903 | 16,903 |
| Country-Year FE | Yes | Yes | Yes | Yes | Yes |
| Spatial Cut-off (miles) | 20 | 20 | 20 | 20 | 20 |
| Temporal Cut-off (months) | 24 | 24 | 24 | 24 | 24 |

*Note*: This table reports OLS estimation results of heterogeneous analysis. We separate the sample into two subgroups based on respondents' shelter types. The non-binary dependent variables have been standardized before estimation. We report the standardized coefficients from the estimations based on Equation (3). The variable *Wildfire* is a binary indicator equal to one if a wildfire occurred within 5 miles of the respondent's village in two years preceding the survey, and zero if the nearest wildfire occurred between 7 miles and 20 miles during the same period. All specifications include county-by-year fixed effects to account for unobserved heterogeneity and national-level temporal trends. Robust standard errors are clustered at the subnational level, with their standardized form reported in parentheses. Statistical inference of coefficients is based on two-sided t-tests. *** $p<0.01$, ** $p<0.05$, * $p<0.1$.

**Supplementary Table 51** Heterogeneity: Roof flammability

| Dept. Vars. | Voting | Contacting local govt. | Attending com. meetings | Collective issue-raising | Protest |
|---|---|---|---|---|---|
| | (1) | (2) | (3) | (4) | (5) |
| **Panel A: With Inflammable Roof** | | | | | |
| Wildfire | 0.1150*** | 0.2590*** | 0.6260*** | 0.5210*** | 0.0652 |
| | (0.0344) | (0.0890) | (0.1390) | (0.1050) | (0.0793) |
| Observations | 35,499 | 37,859 | 37,859 | 37,859 | 37,859 |
| **Panel B: With Non-inflammable Roof** | | | | | |
| Wildfire | -0.1850 | -0.3190 | -0.1300 | -0.2970 | -0.0141 |
| | (0.1430) | (0.4780) | (0.2830) | (0.3640) | (0.2710) |
| Observations | 13,459 | 12,467 | 12,467 | 12,467 | 12,467 |
| Country-Year FE | Yes | Yes | Yes | Yes | Yes |
| Spatial Cut-off (miles) | 20 | 20 | 20 | 20 | 20 |
| Temporal Cut-off (months) | 24 | 24 | 24 | 24 | 24 |

*Note*: This table reports 2SLS estimation results of heterogeneous analysis. We separate the sample into two subgroups based on respondents' housing materials. The non-binary dependent variables have been standardized before estimation. We report the standardized coefficients from the estimations based on Equation (5). The variable *Wildfire* is a binary indicator equal to one if a wildfire occurred within 5 miles of the respondent's village in two years preceding the survey, and zero if the nearest wildfire occurred between 7 miles and 20 miles during the same period. All specifications include county-by-year fixed effects to account for unobserved heterogeneity and national-level temporal trends. Robust standard errors are clustered at the subnational level. Statistical inference of coefficients is based on two-sided t-tests. *** $p<0.01$, ** $p<0.05$, * $p<0.1$.

**Supplementary Table 52** Heterogeneity: Roof flammability–OLS

| Dept. Vars. | Voting | Contacting local govt. | Attending com. meetings | Collective issue-raising | Protest |
|---|---|---|---|---|---|
| | (1) | (2) | (3) | (4) | (5) |
| **Panel A: With Inflammable Roof** | | | | | |
| Wildfire | 0.0173*** | 0.0500** | 0.0767*** | 0.0842*** | 0.0261 |
| | (0.0066) | (0.0197) | (0.0270) | (0.0234) | (0.0172) |
| Observations | 35,499 | 37,859 | 37,859 | 37,859 | 37,859 |
| **Panel B: With Non-inflammable Roof** | | | | | |
| Wildfire | -0.0251*** | 0.0277 | 0.0209 | 0.0261 | -0.0130 |
| | (0.0083) | (0.0332) | (0.0248) | (0.0305) | (0.0239) |
| Observations | 13,459 | 12,467 | 12,467 | 12,467 | 12,467 |
| Country-Year FE | Yes | Yes | Yes | Yes | Yes |
| Spatial Cut-off (miles) | 20 | 20 | 20 | 20 | 20 |
| Temporal Cut-off (months) | 24 | 24 | 24 | 24 | 24 |

*Note*: This table reports OLS estimation results of heterogeneous analysis. We separate the sample into two subgroups based on respondents' housing materials. The non-binary dependent variables have been standardized before estimation. We report the standardized coefficients from the estimations based on Equation (3). The variable *Wildfire* is a binary indicator equal to one if a wildfire occurred within 5 miles of the respondent's village in two years preceding the survey, and zero if the nearest wildfire occurred between 7 miles and 20 miles during the same period. All specifications include county-by-year fixed effects to account for unobserved heterogeneity and national-level temporal trends. Robust standard errors are clustered at the subnational level. Statistical inference of coefficients is based on two-sided t-tests. *** p<0.01, ** p<0.05, * p<0.1.

## 5 Supplementary Notes

Owing to space limitations and for the sake of maintaining logical flow in the main text, we have relegated the following discussion, which emerged during the peer-review process, to this supplementary section.

**OLS–IV gap.** This gap can be interpreted in light of two complementary considerations: possible downward bias in OLS and the local nature of the IV estimand. OLS may understate the effect of wildfire exposure if wildfire-prone areas are negatively correlated with unobserved determinants of political participation, such as remoteness, weaker electoral integration, or lower access to public institutions [6, 7]. In addition, because the instrument shifts wildfire exposure through meteorological fire-risk variation interacted with predetermined land-cover flammability, the IV estimate is closer to a local average treatment effect (LATE) for observations whose wildfire exposure is shifted by the instrument, rather than a population-wide ATE.

**Migration.** We regress LandScan grid-level population counts on wildfire exposure within 5 miles by the same IV specification. The results show a statistically significant negative impact of wildfire exposure on local population size, suggesting that some wildfire-affected individuals are "missing" from the Afrobarometer survey. Due to the unavailability of migration information in the Afrobarometer sample, we cautiously conclude that our estimated coefficients provide a lower bound on the average effect for the ex ante at-risk population living within 20 miles of a wildfire.

## References


[1] Holm, S. A simple sequentially rejective multiple test procedure. Scandinavian Journal of Statistics **6**, 65–70 (1979).

[2] Roodman, D. Fitting fully observed recursive mixed-process models with cmp. The Stata Journal **11**, 159–206 (2011).

[3] Beck, H. E. et al. Present and future köppen-geiger climate classification maps at 1-km resolution. Scientific Data **5**, 180214 (2018).

[4] Simpson, N. P. et al. Climate change literacy in Africa. Nature Climate Change **11**, 937–944 (2021).

[5] Wucherpfennig, J., Weidmann, N. B., Girardin, L., Cederman, L.-E. & Wimmer, A. Politically relevant ethnic groups across space and time: Introducing the geoepr dataset. Conflict Management and Peace Science **28**, 423–437 (2011).

[6] Cantoni, E. A precinct too far: Turnout and voting costs. American Economic Journal: Applied Economics **12**, 61–85 (2020).

[7] Dyck, J. J. & Gimpel, J. G. Distance, turnout, and the convenience of voting. Social Science Quarterly **86**, 531–548 (2005).
