# Supplementary material for: Wildfire exposure and political participation in Africa
Source: Nat Commun. 2026 Sep 12;17:10760. doi: 10.1038/s41467-026-77404-4 (PMC13662189; doi:10.1038/s41467-026-77404-4)
Supplement: Supplementary file 2 [file 41467_2026_77404_MOESM2_ESM.pdf]

nature portfolio

Corresponding author(s): Lina Meng
Pengfei Liu
Lu lin

Last updated by author(s): Aug 20, 2026

# Reporting Summary

Nature Portfolio wishes to improve the reproducibility of the work that we publish. This form provides structure for consistency and transparency in reporting. For further information on Nature Portfolio policies, see our Editorial Policies and the Editorial Policy Checklist.

## Statistics

For all statistical analyses, confirm that the following items are present in the figure legend, table legend, main text, or Methods section.

| n/a | Confirmed | |
|---|---|---|
| ☐ | ☒ | The exact sample size (*n*) for each experimental group/condition, given as a discrete number and unit of measurement |
| ☐ | ☒ | A statement on whether measurements were taken from distinct samples or whether the same sample was measured repeatedly |
| ☐ | ☒ | The statistical test(s) used AND whether they are one- or two-sided<br>*Only common tests should be described solely by name; describe more complex techniques in the Methods section.* |
| ☐ | ☒ | A description of all covariates tested |
| ☐ | ☒ | A description of any assumptions or corrections, such as tests of normality and adjustment for multiple comparisons |
| ☐ | ☒ | A full description of the statistical parameters including central tendency (e.g. means) or other basic estimates (e.g. regression coefficient) AND variation (e.g. standard deviation) or associated estimates of uncertainty (e.g. confidence intervals) |
| ☐ | ☒ | For null hypothesis testing, the test statistic (e.g. *F*, *t*, *r*) with confidence intervals, effect sizes, degrees of freedom and *P* value noted<br>*Give P values as exact values whenever suitable.* |
| ☒ | ☐ | For Bayesian analysis, information on the choice of priors and Markov chain Monte Carlo settings |
| ☐ | ☒ | For hierarchical and complex designs, identification of the appropriate level for tests and full reporting of outcomes |
| ☐ | ☒ | Estimates of effect sizes (e.g. Cohen's *d*, Pearson's *r*), indicating how they were calculated |

*Our web collection on statistics for biologists contains articles on many of the points above.*

## Software and code

Policy information about availability of computer code

| | |
|---|---|
| Data collection | All data obtained for this study was eventually saved as ".dta" format files for analysis and edited in Stata(Version 18 MP, open source packages: ftools, require, reghdfe, ivreghdfe, coefplot, gtools, ranktest and ivreg2). During the data processing, some data was stored and edited in ArcGIS pro 3.1.5 (arcpy) through Python(Version 3.9, open source packages used: numpy, pandas and geopandas) or UI. |
| Data analysis | All data was analysed in Stata(Version 18 MP, open source packages used: ftools, require, reghdfe, ivreghdfe, coefplot, gtools, ranktest and ivreg2) |

For manuscripts utilizing custom algorithms or software that are central to the research but not yet described in published literature, software must be made available to editors and reviewers. We strongly encourage code deposition in a community repository (e.g. GitHub). See the Nature Portfolio guidelines for submitting code & software for further information.

## Data

Policy information about availability of data

All manuscripts must include a data availability statement. This statement should provide the following information, where applicable:

- Accession codes, unique identifiers, or web links for publicly available datasets
- A description of any restrictions on data availability
- For clinical datasets or third party data, please ensure that the statement adheres to our policy

The Global Fire Atlas data are available at https://essd.copernicus.org/articles/11/529/2019/.
The Afrobarometer geocoded data (Rounds 3--6) are available at https://www.afrobarometer.org/data.

The ERA5 reanalysis data are available from the Copernicus Climate Change Service (https://cds.climate.copernicus.eu/datasets/reanalysis-era5-single-levels?tab=overview).
The MODIS fire product (MOD14/MYD14) is available from NASA LP DAAC (https://lpdaac.usgs.gov).
The UCDP conflict data are available at https://ucdp.uu.se/.
The GeoEPR-ETH v2 data are available at https://icr.ethz.ch/data/epr/geoepr/v2.0/.
The climate-literacy dataset can be sourced from the study of Simpson et al.
The individual-level analysis dataset and replication code are available in a Code Ocean capsule at https://codeocean.com/capsule/6875400/tree and will be published alongside the article with a permanent DOI.

## Research involving human participants, their data, or biological material

Policy information about studies with human participants or human data. See also policy information about sex, gender (identity/presentation), and sexual orientation and race, ethnicity and racism.

| | |
|---|---|
| Reporting on sex and gender | The term age and gender used in this study is based on Self-reporting measurement. |
| Reporting on race, ethnicity, or other socially relevant groupings | Race and ethnicity group are used as proxy of social status. The groupings are based on self-reporting race and GeoEPR-ETH Version 2.0 dataset. |
| Population characteristics | No |
| Recruitment | No |
| Ethics oversight | No |

Note that full information on the approval of the study protocol must also be provided in the manuscript.

# Field-specific reporting

Please select the one below that is the best fit for your research. If you are not sure, read the appropriate sections before making your selection.

☐ Life sciences ☒ Behavioural & social sciences ☐ Ecological, evolutionary & environmental sciences

For a reference copy of the document with all sections, see nature.com/documents/nr-reporting-summary-flat.pdf

# Behavioural & social sciences study design

All studies must disclose on these points even when the disclosure is negative.

| | |
|---|---|
| Study description | This study uses a quantitative longitudinal research design to explore the influences from large-scale wildfires on Africans' political participation. The analysis is carried out at the individual level and is based on information from over 100, 000 individual observations across 34 African countries. By combining high-resolution wildfire data with geo-coded Afrobarometer data on political participation, we estimate the causal impact of large-scale wildfire and mechanism behind with an instrumental variable research design. Abundant individual level dataset also allows us to investigate how different demographic factors, natural factors and even individual characteristics would moderate this effect. |
| Research sample | The analysis of this study was carried out at the individual level. In total, the sample covers 101, 292 individual observations from 34 African countries in year 2005, 2006, 2008, 2010 and 2011 to 2015. Dependent variables all come from Afrobarometer, which is a pooled cross-sectional series of public opinion surveys and is based on a random, multistage sampling procedure. The sample is divided into 2 parts. Part 1 is used for analysis about election participation, covering 69, 940 observations; while part 2 is for analysis about participation frequency of contacting local councilors, attendance of community meeting, collective issue-raising and demonstration, covering 83, 206 observations. There's some overlap between these 2 subsamples. |
| Sampling strategy | To construct the analytical sample for our baseline model, firstly, we gather the survey data covering 101, 292 observations from Afrobarometer, which produces its large and representative survey sample by random, multi-stage sampling procedure. Then we combine this dataset with high-resolution wildfire data. |
| Data collection | A range of data sources are used to study the impacts of large-scale wildfire on political participation. To measure political participation, we attain geo-coded survey data about individuals political participation and other characteristics from Afrobarometer organization. The wildfire dataset is sourced from the Global Fire Atlas, a dataset that tracks the day-to-day dynamics of individual fires. Conflict data is sourced from the Uppsala Conflict Data Program (UCDP) database. The ethnic group distribution and status are sourced from the GeoEPR-ETH Version 2.0 dataset. We construct instrumental variables for wildfire exposure by leveraging annual temperature, precipitation and windspeed data from fifth generation ECMWF atmospheric reanalysis of the global climate(ERA5), together with landcover data from Moderate Resolution Imaging Spectroradiometer (MODIS). |
| Timing | Data collection and preparation took place from 2023 to 2026. |
| Data exclusions | As we use the individual level dataset, we inevitably lose some observations. In baseline analysis, some data was excluded due to "doughnut" identification setting. In the baseline analysis, mechanism analysis, sensitivity analysis or heterogeneous analysis, there could also be omitted observations because of missing values of additional variables in those observation. And it was verified by |

| | |
|---|---|
| | other specifications in the sensitivity analysis that these observations would not influence the conclusions. In the analysis of election or long-term impacts, some observations are lost since our wildfire data does not contain data from years earlier than 2000. |
| Non-participation | *State how many participants dropped out/declined participation and the reason(s) given OR provide response rate OR state that no participants dropped out/declined participation.* |
| Randomization | Our identification relies on wildfire exposure induced by exogenous metrological conditions and pre-determined land cover combustibility. Specifically, interaction between temperature and land cover combustibility, interaction between precipitation and land cover combustibility, together with wind speed are applied as instrumental variables, while temperature, precipitation and land cover combustibility are controlled throughout all specifications to satisfy the exclusion restriction. We performed various sensitivity tests to show that our models are robust to variations in the sample, measurement, covariates controlled and specifications used. |

# Reporting for specific materials, systems and methods

We require information from authors about some types of materials, experimental systems and methods used in many studies. Here, indicate whether each material, system or method listed is relevant to your study. If you are not sure if a list item applies to your research, read the appropriate section before selecting a response.

## Materials & experimental systems

| n/a | Involved in the study |
|---|---|
| ☒ | ☐ Antibodies |
| ☒ | ☐ Eukaryotic cell lines |
| ☒ | ☐ Palaeontology and archaeology |
| ☒ | ☐ Animals and other organisms |
| ☒ | ☐ Clinical data |
| ☒ | ☐ Dual use research of concern |
| ☒ | ☐ Plants |

## Methods

| n/a | Involved in the study |
|---|---|
| ☒ | ☐ ChIP-seq |
| ☒ | ☐ Flow cytometry |
| ☒ | ☐ MRI-based neuroimaging |

## Plants

| | |
|---|---|
| Seed stocks | *Report on the source of all seed stocks or other plant material used. If applicable, state the seed stock centre and catalogue number. If plant specimens were collected from the field, describe the collection location, date and sampling procedures.* |
| Novel plant genotypes | *Describe the methods by which all novel plant genotypes were produced. This includes those generated by transgenic approaches, gene editing, chemical/radiation-based mutagenesis and hybridization. For transgenic lines, describe the transformation method, the number of independent lines analyzed and the generation upon which experiments were performed. For gene-edited lines, describe the editor used, the endogenous sequence targeted for editing, the targeting guide RNA sequence (if applicable) and how the editor was applied.* |
| Authentication | *Describe any authentication procedures for each seed stock used or novel genotype generated. Describe any experiments used to assess the effect of a mutation and, where applicable, how potential secondary effects (e.g. second site T-DNA insertions, mosiacism, off-target gene editing) were examined.* |
