# Supplementary material for: Wildfire exposure and political participation in Africa
Source: Nat Commun. 2026 Sep 12;17:10760. doi: 10.1038/s41467-026-77404-4 (PMC13662189; doi:10.1038/s41467-026-77404-4)
Supplement: Supplementary file 3 [file 41467_2026_77404_MOESM3_ESM.pdf]

nature portfolio

Peer Review File

Wildfire Exposure and Political Participation in Africa

Corresponding Author: Dr Lina Meng

This file contains all reviewer reports in order by version, followed by all author rebuttals in order by version.

Version 0:

Reviewer comments:

Reviewer #1

(Remarks to the Author)
This paper is novel in that (a) the impact of wildfires on political attitudes and behavior has been understudied in the African context; and (b) most studies focus on electoral behavior and attitudinal change; there is less analysis in the literature of other forms of behavior, so the analysis presented here is welcome.

Suggestions for improvement:

The paper is very undertheorized; there is no theoretical framework and little indication of how the study builds on previous research. This makes it difficult to interpret the substantive import of the findings.

How do the authors know that the location of the respondents at the time of interview is the same as their location at the time of fire? Wildfires often displace people, such that the location of the respondents after the fact may well not correspond to their fire experience.

Two-way fixed effects can generate misleading casual inferences, as detailed by Imai and Kim (2021), de Chaisemartin and d'Haultfoeille (2020; 2024) and Callaway and Sant'Anna (2020). The authors should consider using one of the estimators developed by these authors.

The authors employ exposure windows of 12 and 60 months. This seems somewhat arbitrary. The analysis of persistence would be more convincing if a large number of windows were examined.

The relationship between fires and economic wellbeing needs to be unpacked more fully. Who is being negatively impacted and how? It would be useful to examine heterogeneity by livelihood category. And also by prior asset accumulation, in that those with assets are typically better able to weather disasters.

References

Callaway B and Sant'Anna PHC. 2020. "Difference in-differences with multiple time periods." Journal of Econometrics 225(2): 200–230.

de Chaisemartin C and D'Haultfœuille X. 2020. "Two-way fixed effects estimators with heterogeneous treatment effects." American Economic Review 110(9): 2964–2996.

de Chaisemartin, Clément Xavier D'Haultfœuille. 2024. Two-way Fixed Effects and Differences-in-Differences Estimators in Heterogeneous Adoption Designs," working paper, https://arxiv.org/pdf/2405.04465.

Imai K and Kim IS. 2021. "On the use of two-way fixed effects regression models for causal inference with panel data." Political Analysis 29(3): 405–415.

(Remarks on code availability)

Reviewer #2

(Remarks to the Author)
This paper addresses an important question: how exposure to extreme climate events affects political participation, by examining the effect of wildfires on a range of political engagement indicators across African countries. It relies on survey data from Afrobarometer, which allows the authors to account for a wide range of individual-level characteristics. While the authors' efforts to assemble such a massive dataset are commendable, and the potential for making important contributions to the scientific debate is clear, I have two central concerns with the study.

* 1. Causality

The paper does not employ a research design that allows it to establish a causal link between exposure to wildfires and political behaviors. The analysis is based on repeated cross-sectional survey data, which are analyzed with a model where the unit of analysis is the individual. The treatment is defined by respondents' location within 5 miles of a wildfire during the 24 months preceding the survey. The control group includes respondents residing in areas between 7 and 20 miles from the nearest wildfire. This choice is justified as a way to “reduce measurement error” (p. 4), but I suppose the intention is rather to mitigate concerns about confounders. The model further includes fixed effects by year and country.

The key point is that exposure to a wildfire is not a random event. Wildfires likely happen in areas having certain characteristics. The use of country-level fixed effects cannot account for the subnational differences that are likely to lead to both the eruption of wildfires and differences in political participation. The evidence from Table 2, which is now presented as evidence on the mechanism, may as well be (and most likely is) evidence of such a selection problem, because it does not present evidence on changes in material deprivation following the fire. It rather shows that areas closer to wildfires are systematically different from control areas.

The instrumental variable approach used in Table 2 is also problematic. The models assess the effect of deprivation on political mobilization by instrumenting deprivation with exposure to wildfires. This is presented as a strategy to “address potential endogeneity” (p. 6). However, what is missing is a discussion of the assumptions needed for causal identification. To be valid, an instrumental variable needs to meet the exclusion restriction: wildfires should affect political participation only through material deprivation. This is hard to prove.

Research in political science has so far developed more appropriate techniques to address these kinds of questions. One example is Pianta and Rettl 2025 (forthcoming in the American Journal of Political Science, not cited). They examine the effect of wildfires in Brazil on support for green policies. They adopt a range of strategies to assess causality, including difference-in-differences and (well-justified) instrumental variables.

My recommendation for the present study is to move the unit of analysis from the individual to the smallest subnational unit at which Afrobarometer is representative and implement a staggered difference-in-differences design. This would allow the authors to test pre-treatment trends and provide evidence of a causal linkage.

* 2. Framing

The paper currently engages too superficially with prior political science research on extreme weather events. Key recent publications are not included (e.g., Pianta and Rettl AJPS 2025; Hilbig and Riaz JOP 2024; Cremaschi and Stanig JOP 2025). The result is that the paper does not clearly highlight that, even in places where climate literacy is high, exposure to extreme weather events does not necessarily translate into higher environmental concerns and pro-environment mobilization. Preoccupation with the material consequences of disasters has already been identified as a key channel that can nullify the pro-environmental effects of exposure to extreme weather events. Furthermore, these papers also highlight how the response by incumbent politicians is a key channel that can shape the effect of extreme weather events on political behavior. This key aspect is not mentioned in the current draft paper and should be integrated into the theoretical framework. I would recommend checking these papers and recent references therein to provide a more accurate picture of the state of the literature and clarify the authors' contribution.

(Remarks on code availability)

Reviewer #3

(Remarks to the Author)
This article intervenes in growing debates about the relationship between extreme weather or disaster exposure and political attitudes, linking wildfire exposure in Africa to increased political participation. I think topically this is quite

interesting, and the geo-located Afrobarometer data that the authors use here is a dataset I view as an underutilized as a resource in climate opinion scholarship. Efforts like this one to merge this expansive dataset with geophysical data are thus welcome.

However, I am not yet persuaded that the article sustains it's key claims, and would need the authors to much more fulsomely explicate and justify their inferential strategy before seeing this research published in a venue such as Nature Communications.

Here, my major concerns relate to the article's causal identification and, in particular, the minimal discussion provided to elaborate this identification despite the substantial causal language deployed throughout.

First, the article's core specification presumes exogeneity of wildfire exposure. The robustness checks used here are, on balance, helpful and appropriately vary the way that the treatment is operationalized. Other specifications are reported, but I agree with the paper's decision to foreground a simple LPM model in this instance as the core reported specification.

What is less clear though is whether this exogeneity assumption itself is credible. The authors restrict their non-treated data to people living in villages between 7 and 20 miles away from a wildfire, something they ascribe to an effort to "reduce measurement error" (page 4). This did not quite make sense to me, as restricting the control group presumably would have been done in order to make more plausible the as-if random assignment of wildfires between treated and non-treated villages. But the authors make no particular effort to guide the reader in assessing the viability of this assumption. It is, of course, helpful that the authors run a model controlling for various potential confounders, but they do not report anywhere the relative balance/imbalance on these measures for the treatment vs. control groups. To the degree that the implicit exogeneity assumptions here hold, we should expect largely balanced results on this front, but the absence of such a table makes me suspect imbalance and, in turn, does moderate the degree to which I am persuaded by the key finding. We are offered little to no discussion of treatment assignment dynamics (e.g. where and how wildfires occur, and how their spatial extents might correlate with patterns of economic and political development).

This opaque empirical consideration then structures so much of the follow-up mechanism and heterogeneous effect work that follows. For instance, Section 2.3 is somewhat surprising relative to existing literatures, but without having a better sense of whether the underlying specification is credibly identified from a causal perspective, it is equally possible that this stability is merely an artifact of the self-reported variables as measured in the Afrobarometer survey. The same is true for the many heterogenous treatment effects subsequently probed. The concerns for the reader remain. For example, is the relationship between ethnic groups + participation structured by considerations beyond wildfire exposure? Could wildfire exposure simply be picking up some variable that is correlated with both exposure and socioeconomic issues, creating apparent correlations. The reader can't fully decide based on the evidence presented.

A second example of the inadequate elaboration of causal considerations is in the mechanism section and the presentation of a very ambiguous instrumental variable model. Noting the serious endogeneity concerns here, they propose an instrumental variable solution, but offer no real rationale for why and how their chosen instrument meet the necessary assumptions for the paper's analysis. (In particular, they do not discuss the necessary exclusion restrictions that must hold and, in fact, given their instrument is "first principal component of household shortages of essential goods, including water, food, medicine and cash, over the previous week when surveyed, calculated by PCA method" - I cannot follow why applying a PCA analysis to self-reported endogenous measures from the same survey as the outcome variable offers any causal leverage.) I therefore find unpersuasive the sustained claims of causality that accompany this section too.

Finally, I would encourage the article to dialog more with several literatures in political science on related topics. First, there is a growing literature on crime victimization and political participation (including a Bateson American Political Science Review article from several years ago that also looked at within-Afrobarometer correlations on this topic). Second, since wildfire exposure is a bundled treatment with incumbent / government responses to that wildfire and its aftereffects, consideration of whether and how potential participation impacts are a function of the disaster itself or policy responses to it could be elaborated. This type of mechanism is not tested or discussed theoretically, with the focus being on reactions to wildfire exposure only, and not its bundled consequences.

(Remarks on code availability)

Version 1:

Reviewer comments:

Reviewer #1

(Remarks to the Author)
I am satisfied that the authors have addressed my concerns, and this paper is close to being ready to publish.

There are only two small points that I would suggest the authors consider:

I am pleased to see that the analysis of mechanism has been further developed. The mechanisms, as described, look very much like they involve clientelistic mobilization. It would make sense in the conclusion to comment on the normative implications of the use of disaster relief for clientelistic purposes.

Page 3, penultimate line “while not concentrating” should be “while concentrating less”, as there are papers on this topic.

(Remarks on code availability)

Reviewer #2

(Remarks to the Author)
I find that the paper has improved substantially. The revised empirical strategy based on an IV design is more convincing, and the theoretical discussion is now more thorough and better integrated with the empirical analysis. That said, I still have a few outstanding concerns regarding the empirical implementation that require clarification.

1. The treatment variable (proximity to wildfires) is now operationalized as a dichotomous indicator distinguishing respondents who live close to a wildfire from those who live farther away. This choice introduces an arguably arbitrary modeling assumption. Using continuous distance from the wildfire would seem a more natural specification. In addition, I would like to see results that include respondents located between 7 and 20 miles from the nearest wildfire. Their exclusion is currently justified by the dichotomous operationalization of the treatment and the need to reduce measurement error. However, it would be reassuring to see robustness checks showing how sensitive the results are to this modeling choice. I would strongly encourage using a continuous measure of distance to wildfires—including all respondents within the relevant radius—as the main specification.

2. First-stage estimates and the corresponding F-statistics for the main models are neither reported nor discussed. This is a notable omission, given their central role in assessing instrument relevance and strength. In addition, reporting reduced-form estimates would also be considered good practice.

3. The instruments are measured well before the occurrence of the wildfire. Weather variables (precipitation, temperature, and wind speed) are measured two years prior to the interview, while land flammability is measured three years before. It is unclear why this timing is chosen. If the IV strategy is intended to exploit exogenous variation in wildfire exposure, it seems that the relevant variation should be measured as close as possible to the event itself. For instance, Pianta and Rettl (2025) rely on weather measures from the week preceding the wildfire. Relatedly, I do not understand why land flammability is operationalized as a binary indicator, given that it is likely available as a continuous index in the original data.

(Remarks on code availability)

Reviewer #3

(Remarks to the Author)
The authors here should be commended for the seriousness with which they took a very challenging set of initial reviews, including my own. Those reviews particularly pushed on whether the article could defend its causal claims, which is a large focus of the revisions offered here and the authors' response memo.

I had mixed reactions to this revised manuscript. It is much cleaner and stronger than the initial version, focussing attention on the empirical claims that are most credible and offering a stronger theoretical and mechanismic account for these findings.

However, despite these improvements, one large reservation remains. The manuscript now pivots to (more appropriately) anchor it's identification strategy with an instrumental variables approach. Yet, the manuscript still does not provide enough context and information to allow for a reader to fully adjudicate this approach's credibility.

Specifically, the paper now proposes that a custom measure of "wildfire risk" can instrument for wildfire exposure and therefore circumvent the serious endogeneity concerns that challenged some of the first draft's findings. To the best of my knowledge, the specific instrument advanced here is novel (and, indeed, the manuscript does not cite other literature in advancing this specific instrument). But this also means that there is a higher bar for unpacking the instrument here. I have a few comments on this.

First, this is not the first paper to instrument for wildfire exposure, and it would be good to review and contrast the papers' instrument with the other approaches taken in the literature. Some of these are similar in part (e.g. lagged drought) while others are different and less applicable to this specific dataset (e.g. lightning strike intensity).

Second, the paper needs to make its instrument more clear. In particular, the "flammability" metric that is used to construct the instrument is left underspecified. What does this metric measure in terms of land use? How is it measured? What is the data-generating process that shapes land-use flammability, and does the exclusion restriction plausibly hold with respect to that data-generating process? It may well do so! But the manuscript does not provide the detail or information necessary for that determination to be made, either by the paper or by interested readers. Even the methods section only provides a single

line on this flammability metric and offers no real clarity on what it measures and how it is constructed.

Third, the discussion of the exclusion restriction left me with some questions. It seems that the authors are concerned that the exclusion restriction does not hold in this case with simple measures of precipitation or temperature alone, and thus feel the need to interact these with the flammability measure. But this is very hard to follow for the reader, and I think requires more clear writing and justification. It is also possible that changing the measurement strategy for some of the temperature/precipitation measurements (to focus on long-term structural and lagged conditions) might be a better approach to making the exclusion restriction more credible.

As before much of the paper is very interesting and I appreciate its topic and geographic focus. Were this current draft the original submission, I would have encouraged a revise and resubmit in a journal like Nature Communications. However, I still think a far more fulsome theoretical explanation of the instrument's logic is important, particularly with detail and clarity on the individual elements that go into the instrument's constructions, and their individual and joint data-generating processes. In turn, linking this logic to a literature that has considered this specific treatment before would be useful. The credibility of these identification assumptions are the fulcrum of the paper, and so despite appreciating its other merits, this still feels like a necessary improvement before recommending for publication.

(Remarks on code availability)

Version 2:

Reviewer comments:

Reviewer #1

(Remarks to the Author)
The authors appear to have misunderstood my request for “comment on the normative implications of the use of disaster relief for clientelistic purposes”. When I referred to the normative implications of the clientelization of disaster relief, what I was referring to was the moral and democratic theory implications of these practices. If parties are using disaster relief to bribe voters, this is a form of corruption. It also undermines democratic representation, because it short-circuits voter evaluations of candidate competence and policy positions. It appears, therefore, that this practice is profoundly undemocratic and may lead to democratic backsliding. I invite the authors to reflect on this.

(Remarks on code availability)

Reviewer #2

(Remarks to the Author)
The authors have done a great job to address the challenging comments raised by reviews, including my own, throughout the review process and in this round in particular. This is a strong paper that I feel confident recommending for publication.

(Remarks on code availability)

Reviewer #3

(Remarks to the Author)
Again, I want to commend the authors for their engaged response to my comments, which have remained more challenging than the other reviewers. My reaction to this paper remains mixed, even after the additional (and substantial) revisions, but I am do recognize that the other reviewers are on balance quite positive and that the authors have done serious empirical legwork here to address broader concerns. This empirical work does reassure me in part by offering a suite of robustness checks that help buttress some of the core claims.

Where I am less persuaded, this has to do with what a view as this revision's weaker effort to reassure the reader on the credibility of the instrument itself and the instrument's exclusion restriction. For example, the authors now write: "In the estimation, we control separately for meteorological conditions and land-cover characteristics, so identification comes from the interaction-based component of weather variation in more combustible areas. These controls effectively absorb any alternative pathways through which these meteorological variables might influence political participation". But this is not true mechnically. The controls absorb linear pathways associated with observables, but not unobservables and, critically, not the likely non-linear pathways associated with these observables. My intuition is that metereological settings are precisely where we would expect to see non-linear effects of rainfall on crop yield, livestock, etc. and follow-on interactions with land-cover variables. In short, I think the paper needs to still be softened to make clear that there are substantial causal identification questions that remain unresolved.

In addition, in reading the paper another time, the substantial gap between the magnitude of OLS and 2SLS estimates surprised me. (I should have seen this earlier!) I do think the authors should add some explicit recognition of this, and

provide some speculation on why there is severe downward bias in the OLS.

With these issues made more transparent, I am comfortable deferring to the more positive assessments of the other reviewers, largely because I do think the empirical setting here is understudied and there is real value in careful empirical work in non-US/Europe on this topic.

(Remarks on code availability)

Version 3:

Reviewer comments:

Reviewer #3

(Remarks to the Author)
My comments have been the most demanding of the reviewers and, per my last review, I am nonetheless willing to see this accepted for publication given the importance of the setting. I appreciate the authors further adding caution to their causal identification assumptions, and for adding an interpretation of the OLS/2SLS divergent estimates. I have no further issues beyond the ones I raised previously that the authors have made a good faith effort to address here.

(Remarks on code availability)

Open Access This Peer Review File is licensed under a Creative Commons Attribution 4.0 International License, which permits use, sharing, adaptation, distribution and reproduction in any medium or format, as long as you give appropriate credit to the original author(s) and the source, provide a link to the Creative Commons license, and indicate if changes were made.
In cases where reviewers are anonymous, credit should be given to 'Anonymous Referee' and the source.
The images or other third party material in this Peer Review File are included in the article's Creative Commons license, unless indicated otherwise in a credit line to the material. If material is not included in the article's Creative Commons license and your intended use is not permitted by statutory regulation or exceeds the permitted use, you will need to obtain permission directly from the copyright holder.
To view a copy of this license, visit https://creativecommons.org/licenses/by/4.0/

Authors' Response to Reviews of

Fires and Votes: How Wildfire Exposure Affects Africans' Political Participation

Nature Communications,

RC: ***Reviewers' Comment***, AR: Authors' Response, ☐ Manuscript Text

We sincerely appreciate your constructive comments and valuable suggestions on the previous version of the manuscript. Following the comments, we have substantially revised the paper and provided detailed, point-by-point responses below. These revisions have significantly strengthened the manuscript, and we believe the current version successfully addresses all major concerns. Revisions made in the manuscript are highlighted in BLUE for ease of reference.

1. Reviewer #1

1.1. Comment

RC: ***This paper is novel in that (a) the impact of wildfires on political attitudes and behavior has been understudied in the African context; and (b) most studies focus on electoral behavior and attitudinal change; there is less analysis in the literature of other forms of behavior, so the analysis presented here is welcome.***

AR: We sincerely thank you for the positive evaluation and for recognizing the contributions of our study. We appreciate the reviewer's encouraging comments, and below we provide detailed responses to the concerns raised in the remainder of the report.

1.2. Comment

RC: ***The paper is very undertheorized; there is no theoretical framework and little indication of how the study builds on previous research. This makes it difficult to interpret the substantive import of the findings.***

AR: We thank you for this important comment. We agree with the reviewer that the earlier version did not clearly situate our study within the existing political science literature on climate extremes. In the revised manuscript, we have substantially expanded the "*Introduction*" section to develop a structural theoretical framework, clarify our contribution, and explicitly connect our arguments to recent advances in the literature. These additions are now highlighted in BLUE in the revised text. Below we summarize the major improvements.

First, the revised manuscript provides clearer theoretical clarification on why community-level participation, particularly collective issue-raising, is central in the African context. Prior research on instrumental democracy and local governance shows that political engagement in Africa is driven less by ideological commitments than by expectations of immediate, tangible benefits [1, 2, 3, 4]. Because national elections are infrequent and many communities face material deprivation and limited institutional capacity, activities such as contacting local officials, attending meetings, and raising collective concerns serve as the most direct channels for responding to climate-induced shocks. Exploring the community-level political participations within this institutional landscape extends existing work, which has focused primarily on electoral behaviour in advanced democracies [5, 6, 7, 8, 9].

Second, in response to your comment and in light of other reviewers' suggestions, we incorporate insights from the recent political science literature on climate and environmental shocks to ground a formal theoretical framework for understanding political response to wildfires in Africa [10, 11, 12]. The revised manuscript now includes a framework that identifies material deprivation and government responsiveness as the primary mechanisms linking wildfire exposure to political participation in Africa. This framework clarifies the conditions under which climate-induced disasters generate political action in low-capacity settings and highlights how such mechanisms diverge from patterns documented in high-capacity democracies.

We also cite the revised contents in the manuscript within the box for ease of reference.

Paragraph 2 on Page 3 – Paragraph 1 on Page 4:

Understanding how climate-induced disasters shape political participation in Africa requires situating the analysis in the continent's unique institutional and political realities. Existing research on climate extremes has mainly examined electoral behaviour and environmental attitudes in developed democracies [5, 6, 7, 8, 9], whereas political engagement in Africa often follows a more instrumental logic: citizens assess institutions primarily by their ability to deliver immediate, tangible benefits rather than ideological commitments [1, 3, 13]. This instrumental approach has significant implications for how citizens mobilize politically after climate disasters. Furthermore, a notable gap exists in the literature's focus on electoral participation, with insufficient attention given to community-level civic engagement—a gap especially relevant for Africa, where local participation often serves as the main avenue through which citizens influence governance and express demands [4, 2]. Unlike national elections, which are infrequent, community activities such as contacting local officials, attending meetings, and raising collective issues provide ongoing opportunities for citizens to seek redress for immediate concerns. In Africa, with around 70 % of the population under 30 and many communities facing material deprivation and weak institutional capacity, local governance mechanisms are vital channels for grass-roots democracy. The significance of community participation also varies across different African contexts, shaped by decentralization frameworks, ethnic compositions, and institutional capacities, with countries like South Africa, Kenya, Ghana and Rwanda developing distinct local governance innovations that establish formal channels for disaster-affected communities to articulate demands [14, 15, 16].

We propose a theoretical framework centered on material deprivation and government responsiveness as the primary mechanisms linking wildfire exposure to political participation in Africa, departing from models developed in Global North contexts where climate disasters increase environmental concern and generate support for climate-friendly policies [6, 7, 17, 18]. In settings characterized by limited climate literacy, immediate material scarcity, and instrumental orientations toward democracy, wildfires mobilize citizens primarily through disruption of daily life and creation of urgent needs for government response [12, 10, 11]. Climate-induced wildfires generate immediate material consequences, destruction of property, loss of agricultural assets, disruption of food and water access, creating pressing needs that cannot be addressed through individual coping strategies alone. Where democracy is valued instrumentally as a means to securing tangible benefits, material deprivation creates powerful incentives for political mobilization [1, 3]. Citizens engage in community-level political activities not primarily from ideological commitment to environmental protection, but as strategic responses to immediate threats to well-being. Our framework distinguishes between institutionalized participation (voting, contacting councilors, community meetings), representing low-cost activities within existing governance structures, and non-institutionalized participation (collective issue-raising, protests) involving higher costs but necessary when institutionalized channels prove unresponsive [19]. Political responses

vary systematically across populations based on factors affecting both the severity of material losses and the capacity for mobilization, including climate vulnerability, ethnic political status, information accessibility, and socioeconomic resources [20, 21]. By centering community-level participation and theorizing material deprivation and government's bundled reactions as primary mechanisms, we extend understanding of how citizens in the Global South respond to climate extremes through diverse forms of civic engagement, illuminate essential differences between political responses in developed versus developing countries, and provide insights into which communities can mobilize effectively to secure disaster assistance.

1.3. Comment

RC: ***How do the authors know that the location of the respondents at the time of interview is the same as their location at the time of fire? Wildfires often displace people, such that the location of the respondents after the fact may well not correspond to their fire experience.***

AR: We thank you for this comment. Yes, we agree with you that if respondents relocated after experiencing a wildfire, then the exposure measured at their interview location may underestimate their true exposure, biasing the OLS estimates toward zero. In this sense, the OLS results are likely to be a lower bound of the true value. To respond to your comments, we check this issue in the following way.

Given that the Afrobarometer does not contain direct migration information, we examine population changes using high-resolution LandScan grid-level population data[1] , which provides annual estimates of population distributions. We regress annual grid-level population counts on wildfire exposure within five miles by the same IV specification. The results show that there is a statistically significant negative impact of wildfire exposure on local population size, suggesting that some wildfire-affected individuals are "missing" from the Afrobarometer survey. Given the presence of this population loss, our IV estimates remain positive and statistically significant for multiple measures of political participation. Taken together, we cautiously conclude that our estimated coefficients provide a lower bound on the average effect for the ex ante at-risk population living within 20 miles of a wildfire.

For ease of reference, we report the corresponding results below. We also added a footnote in the revised manuscript to classify this issue for readers.

Footnote 1 on Page 9:

[1] We regress LandScan grid-level population counts on wildfire exposure within 5 miles by the same IV specification. The results show a statistically significant negative impact of wildfire exposure on local population size, suggesting that some wildfire-affected individuals are "missing" from the Afrobarometer survey. Due to the unavailability of migration information in the Afrobarometer sample, we cautiously conclude that our estimated coefficients provide a lower bound on the average effect for the ex ante at-risk population living within 20 miles of a wildfire.

[1]LandScan is a globally recognized, R&D100-winning population data platform developed at Oak Ridge National Laboratory (ORNL), `https://landscan.ornl.gov/`.

Table: The relationship between wildfire exposure and population counts

	(1)
VARIABLES	Population
Wildfire	-0.454**
	(0.199)
Observations	83,206
Country-Year FE	Yes
Spatial Cut-off	20 miles
Temporal Cut-off	24 months

Robust standard errors in parentheses

*** p<0.01, ** p<0.05, * p<0.1

Note: The table is estimated by IV specifications, as described in Equation (5) in the revised manuscript. The dependent variable is annual grid-level population counts, sourced from LandScan.

1.4. Comment

RC: ***Two-way fixed effects can generate misleading casual inferences, as detailed by Imai and Kim (2021), de Chaisemartin and d'Haultfoeille (2020; 2024) and Callaway and Sant'Anna (2020). The authors should consider using one of the estimators developed by these authors.***

AR: We thank you for the comment. Following suggestions from other reviewers, we adopt an IV approach to solve the potential endogeneity. As we explained in the correspondence to comment 1.3, we construct wildfire-risk instruments by interacting lagged land-cover flammability with exogenous weather conditions measured two years prior to the interview (average temperature and precipitation), and by including average wind speed from the same period. We also control for the direct effects of temperature, precipitation and land cover to satisfy the exclusion restriction. Under the revised baseline models, our major conclusions still hold. Specifically, wildfire exposure significantly increases both institutionalized and non-institutionalized political participation, especially community meeting attendance, while the effects on frequency of protest participation turn out to be insignificant under the IV strategy. The following Table 1 and text present and illustrate the revised baseline results.

Paragraph 3 on Page 24 – Paragraph 4 on Page 25:

However, the OLS results of Equation (3) are difficult to interpret directly as a causal effect. Causal identification would require that, among respondents living within 20 miles of a wildfire in the two years preceding the survey, their distance to the wildfire is as good as random. This assumption may be difficult to satisfy as human activity and the occurrence of wildfires are often correlated, leading to an omitted variable bias [12]. For example, settlements located in more ecologically fragile areas may be both more fire-prone and more politically active as residents seek government assistance to cope with environmental stress. In such cases, the OLS would conflate the effect of wildfire exposure with underlying social or political conditions.

To address the potential endogeneity, we construct our instrumental variables, named as wildfire risk, by interacting exogenous weather conditions with lagged land-cover flammability. Specifically, we use average precipitation, average temperature, and average wind speed measured two years prior to the interview year ($Tem_{v(i),t-2}, Prep_{v(i),t-2}, Windspeed_{v(i),t-2}$), together with a dummy whether the land cover at the respondent's location was flammable in year three years earlier ($I(LC)_{v(i),t-3}$). Accordingly, the wildfire risk instrument vector is:

$$\mathbf{WildfireRisk}_{v(i),t-2} = \begin{bmatrix} Prep_{v(i),t-2} \cdot I(LC)_{v(i),t-3} \\ Tem_{v(i),t-2} \cdot I(LC)_{v(i),t-3} \\ Windspeed_{v(i),t-2} \end{bmatrix} \tag{4}$$

Our identification strategy relies on that temperature, precipitation, wind speed and land-cover characteristics are plausibly exogenous environmental determinants of wildfire occurrence. These factors shape the likelihood and intensity of wildfires [22, 12]. After controlling for baseline climatic conditions and socioeconomic factors, these environmental variables are not chosen by local communities.

To address the exclusion restriction, we adopt the following approach. By interacting temperature and precipitation with an indicator for whether local land cover is combustible, we isolate the specific

channel through which climatic conditions affect political participation via their impact on wildfire risk. We also control for temperature, precipitation, and land-cover characteristics in the estimation. These controls effectively absorb alternative pathways through which these meteorological variables might influence political participation. As a result, the residual variation in the interaction terms identifies exclusively the component of weather variation that operates through wildfire incidence [23, 12, 24, 25].

We further conduct a falsification test to verify the exclusion restriction by regressing a set of potential mechanism outcomes on the instrumental variables, while simultaneously controlling for wildfire exposure and the full set of baseline covariates. As reported in Table **??**, the estimated coefficients on the individual instruments are generally small and statistically weak across all specifications. Moreover, the joint Wald F-statistics testing the null hypothesis that the coefficients on the three instruments are jointly equal to zero are uniformly low. After applying Holm's step-down adjustment to account for multiple hypothesis testing across outcome groups, none of these joint tests remains statistically significant. Taken together, these results provide additional support for the exclusion restriction by failing to detect systematic associations between the instruments and the proposed mechanism variables, conditional on wildfire exposure and observed confounders.

The full model with the first stage is derived as:

$$\begin{cases} Y_{i,t} = \alpha + \beta_1 \cdot \widehat{Wildfire}_{v(i),t-2} + \beta_{\mathbf{2}} \cdot \mathbf{Control}_{\mathbf{v(i),t}} + C_{c(i)} \times Year_t + \varepsilon_{i,t}, \\ Wildfire_{v(i),t-2} = \eta + \gamma_{\mathbf{1}} \cdot \mathbf{WildfireRisk}_{v(i),t-2} + \gamma_{\mathbf{2}} \cdot \mathbf{Control}_{\mathbf{v(i),t}} + C_{c(i)} \times Year_t + \epsilon_{i,t} \end{cases} \tag{5}$$

where $\mathbf{WildfireRisk}_{v(i),t-2}$ is the instrumental variables of wildfire, calculated by Equation (4), and $\mathbf{Control}_{\mathbf{v(i),t}}$ is the temperature, precipitation, and land-cover characteristics at year t when surveyed. By incorporating country-by-year fixed effects $C_{c(i)} \times Year_t$, we account for unobserved county-specific time shocks such as war, structural economic change, and large-scale social unrest that could otherwise bias our estimates. In the robustness checks, we further control for respondent characteristics, including urban residency status, gender, age, and access to personal information channels. We also adopt alternative model specifications including ordered probit model and ordered instrumented probit model to test the robustness of the estimation.

Table 1 on Page 8:

Table 1: Baseline Effects of Wildfire Exposure on Political Participation

	Institutionalized Form			Non-institutionalized Form	
Dept. Vars.	Voting	Contacting local govt.	Attending com. meetings	Collective issue-raising	Protest
	(1)	(2)	(3)	(4)	(5)
Panel A: OLS Estimation					
Wildfire	0.008*	0.052***	0.084***	0.061***	0.019
	(0.0046)	(0.0147)	(0.0181)	(0.0155)	(0.0119)
Observations	69,940	83,206	83,206	83,206	83,206
Panel B: 2SLS Estimation					
Wildfire	0.078***	0.227***	0.466***	0.270***	0.073
	(0.0204)	(0.0567)	(0.0687)	(0.0563)	(0.0519)
Observations	69,940	83,206	83,206	83,206	83,206
Country-by-Year FE	Yes	Yes	Yes	Yes	Yes
Spatial Cut-off (miles)	20	20	20	20	20
Temporal Cut-off (months)	24	24	24	24	24

Note: Panel A reports the OLS estimation results by Equation (**??**), panel B reports the 2SLS estimation results based on Equation (5). We instrument $Wildfire_{v(i),t-2}$ by $\mathbf{WildfireRisk}_{v(i),t-2}$, constructed by climate conditions in the last two years. The variable *Wildfire* is a binary indicator equal to one if a wildfire occurred within 5 miles of the respondent's village in two years preceding the survey, and zero if the nearest wildfire occurred between 7 miles and 20 miles during the same period. The non-binary dependent variables, including contacting local government officials, attending community meetings, collective issue-raising, and participation in protests, are standardized before estimation. All specifications include country-by-year fixed effects to account for unobserved heterogeneity and national-level temporal trends. Robust standard errors are clustered at the subnational level. *** p<0.01, ** p<0.05, * p<0.1.

1.5. Comment

RC: ***The authors employ exposure windows of 12 and 60 months. This seems somewhat arbitrary. The analysis of persistence would be more convincing if a large number of windows were examined.***

AR: We thank you for this comment. We have now extended our time windows to 7 years (84 months) prior to the interview to assess the robustness of our results. The conclusion remains consistent under this longer observation window. We do not extend the window further because larger lags substantially reduce the number of usable Afrobarometer observations (2005–2015), which would compromise statistical power and cross-country comparability.

We cite the updated results as well as the related contents in the revised manuscript for ease of reference.

Paragraph 1 on Page 12:

We next investigate how long the effect of wildfire exposure persists by changing the exposure window from 12 to 84 months prior to the interview date by the baseline model specified in Equation (5). The results in Extended Figure D1 of Appendix **??** show that, the effect of wildfires on political engagement remains stable over time.[a] The estimated effects on individuals' participation in various political activities are largely stable when the length of the exposure window varies from 12 to 84 months. Panel A in Extended Figure D1 shows that individuals residing closer to wildfire events increase the intensity of contacting local governments by 20.1% to 35.1% standard deviations (S.D. = 0.890). For attendance at community meetings (Panel B), increment of intensity ranges from 37.4% to 64.3% standard deviations (S.D. = 1.310). Regarding collective issue-raising and community engagement, proximity to recent wildfires increases intensity by 17.9% to 35.6% standard deviations (S.D. = 1.294). The effect of wildfire exposure on participation in protests and demonstrations is insignificant. According to the stable effects on contacting local councillors, community meetings attendance and collective issue-raising across different windows, we use a 24-month event window to capture the impact of recent large-scale wildfires in the subsequent analysis. Overall, the results in Extended Figure D1 suggest that wildfire exposure has a persistent and relatively stable effect on individuals' political participation. Prolonged disruption caused by large-scale wildfires could lead to material hardship over time, particularly through the adverse effects on soil quality and agricultural productivity [26].

[a] Given that the dataset lacks wildfire information before 2000, extending the exposure window to 36 months prior to the election would entail substantial sample attrition. Accordingly, we refrain from analysing the long-term impact of wildfire exposure on voting intentions.

Figure D1 on Page 49:

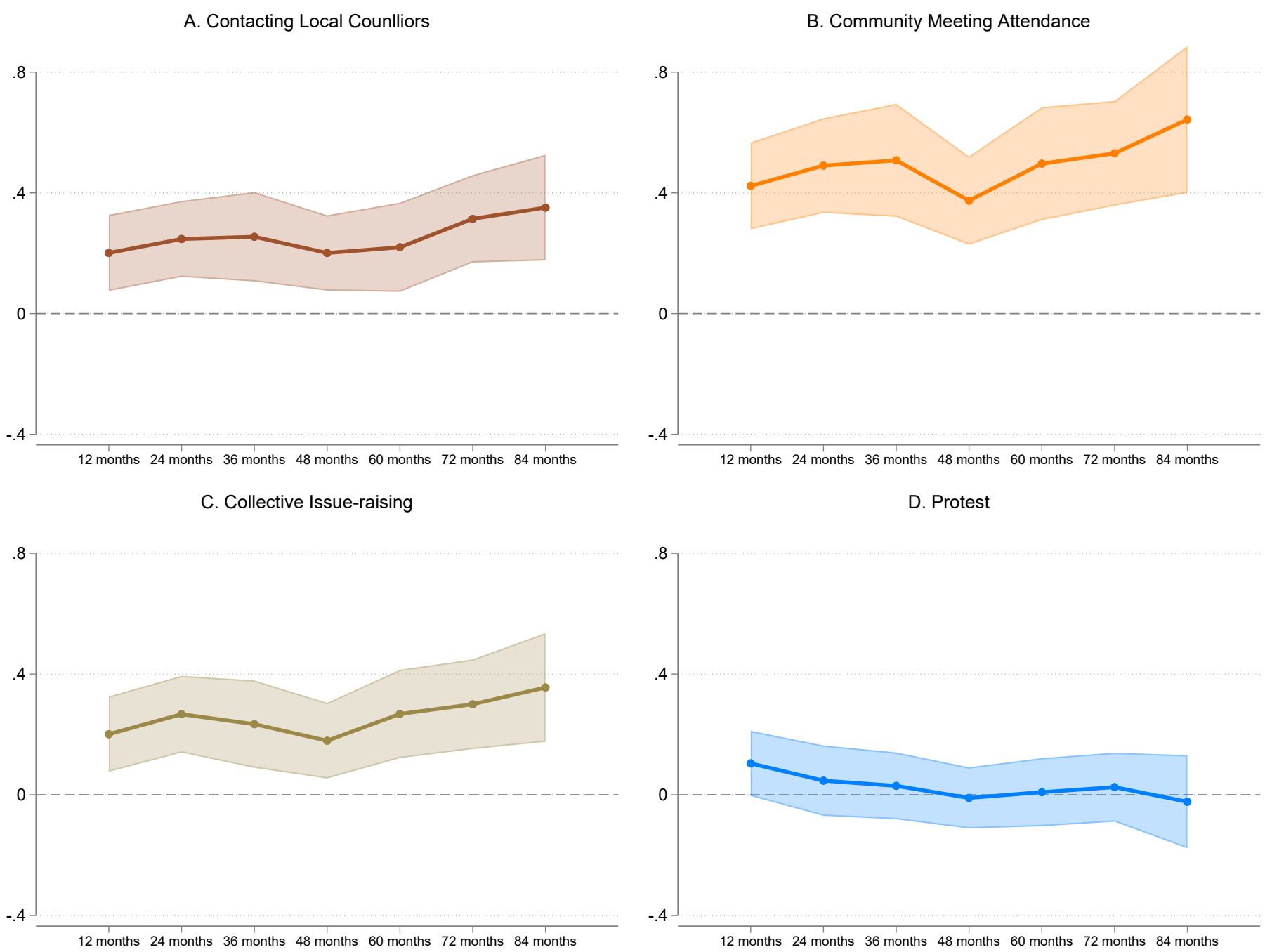

Figure D1: The Long-term Effect of Wildfire Exposure on Political Participation

Note: The figure shows the long-term effect of wildfire exposure on political participation by changing the time window from 0-12 to 73-84 months preceding the survey based on Equation (5), (A) the long-term effect on contacting local councilors; (B) attending community conference; (C) collective issue-raising; (D) participation in protest. Each dot represents a separate estimation, and the gray area indicates the 95% CIs of the estimation. The full tables are reported in Tables **??**–**??**. Given that the dataset lacks wildfire information before 2000, extending the exposure window to 36 months prior to the election would entail substantial sample attrition. Accordingly, we refrain from analysing the long-term impact of wildfire exposure on voting intentions.

1.6. Comment

RC: ***The relationship between fires and economic wellbeing needs to be unpacked more fully. Who is being negatively impacted and how? It would be useful to examine heterogeneity by livelihood category. And also by prior asset accumulation, in that those with assets are typically better able to weather disasters.***

AR: We thank you for the insightful comment. We agree with the reviewer that differences in economic well-being may shape how individuals experience and respond to wildfire exposure. In the revised manuscript, we incorporate heterogeneity analyses by occupation and by wealth. Because the Afrobarometer does not provide detailed measures of household wealth, we use respondents' shelter type as a proxy for household wealth, an established indicator of durable assets in the African context. For occupation, data are available only in Rounds 3 and 6, and the heterogeneity analysis is therefore conducted for those subsamples. Both sets of analyses are estimated using the IV strategy outlined in Equation (5).

We first distinguish between agricultural and non-agricultural workers. While both groups exhibit similar overall sensitivities to wildfire exposure, only non-agricultural workers show increased voting participation following wildfire exposure.

We then classify respondents by housing type: formal versus informal houses (e.g., huts and shacks), to capture differences in household wealth accumulation. Individuals living in formal houses display significantly higher increases in voting, contacting local government, attending community meetings, and participating in collective issue-raising following wildfire exposure. No comparable effects are observed from respondents in informal housing.

In the following, we cite the revised contexts of the heterogeneous analysis by occupation and wealth, and discuss them in the context of the existing literature.

Paragraphs 3 on Page 17 – Paragraphs 2 on Page 18:

> Differences between respondents in agricultural and non-agricultural sectors are modest (Panel D in Figure 5).[a] Individuals in both groups increase their engagement with local officials, attend community meetings more frequently, and participate in collective issue-raising when exposed to nearby wildfires. The main divergence appears in electoral behaviour: only non-agricultural workers show higher voting participation, likely reflecting the correlation between non-agricultural occupations and individuals with higher education levels [27].
>
> For other forms of political activity, we do not detect meaningful differences between the two groups. This pattern contrasts with findings from the Amazon rainforest region, where agricultural producers often benefit economically from large-scale wildfires [12]. The situation in Africa differs substantially from that in South America's Amazon basin: the scale of slash-and-burn activities is much smaller [28], agricultural commercialization is limited, thus wildfires typically generate losses rather than economic gains. These structural differences may explain the absence of divergent political responses across livelihood types.
>
> [a] Notice that only samples in survey Rounds 3 and 6 are included in this analysis since this question is only included in these two survey rounds.

Paragraph 3 on Page 18 – Paragraph 4 on Page 18:

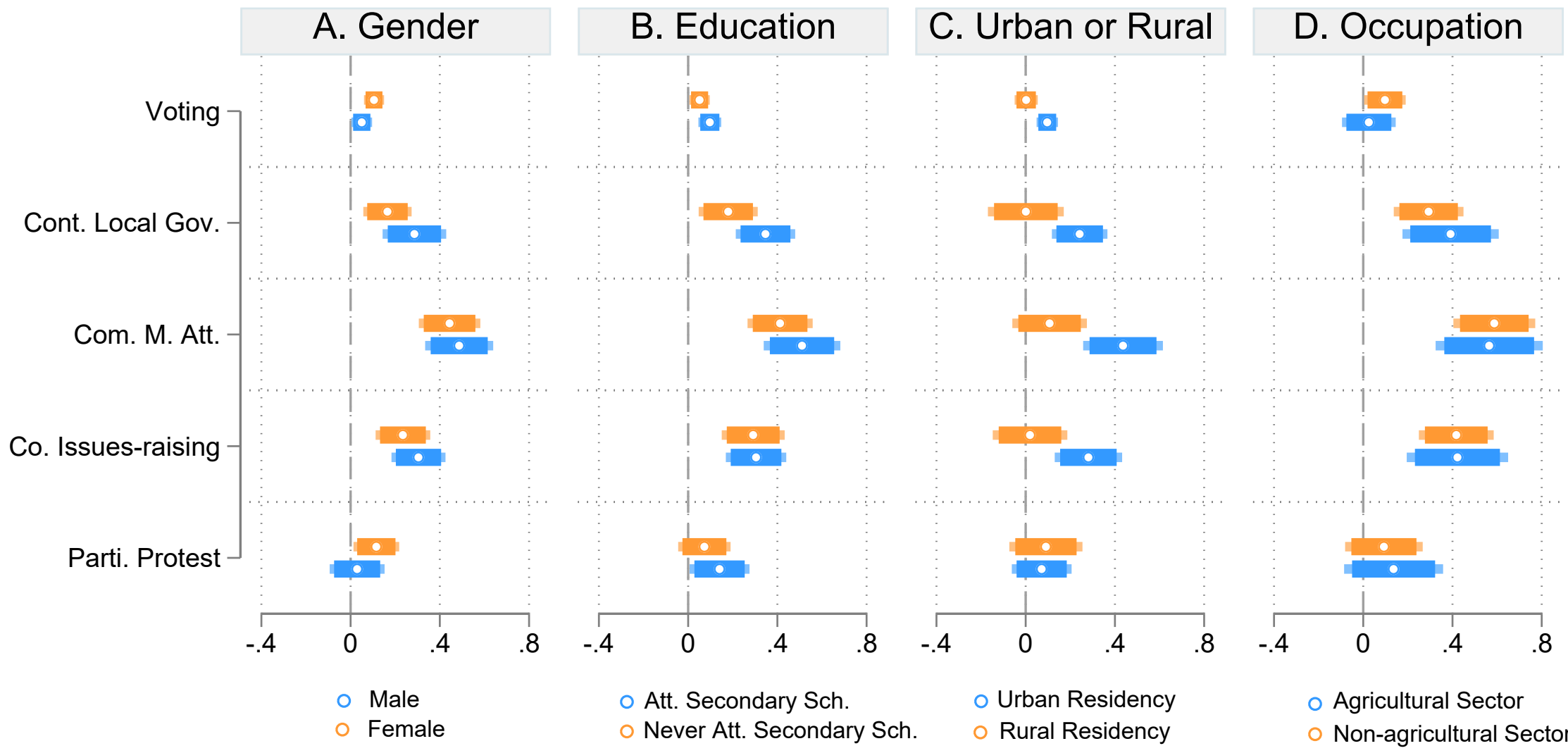

Figure 5: Heterogeneity Analysis: Demographic Characteristics and Economic Conditions

Household wealth is widely recognized as an important source of heterogeneity in response to climate shocks [29]. We focus on housing, a central component of wealth portfolios in rapidly urbanizing African economies [30]. Firstly, we divide the samples based on the flammability of the roofs of the respondents, and find that individuals living in houses with flammable roofs increase their political participation when exposed to nearby wildfires, whereas no such effect is observed among those with non-flammable roofs (Panel A in Fig. 6).

We then distinguish between formal and informal housing structures. As shown by Panel B of Fig. 6, respondents living in informal dwellings show no detectable changes in political engagement following wildfire exposure, in fact they even reduce the participation of demonstration. In contrast, individuals residing in formal housing exhibit increases across nearly all forms of participation (except protest). Specifically, among the latter group, closer wildfire exposure increases the probability of voting by 10.4 per cent (95% CI: 0.051–0.157), and raises the intensity of contacting local government officials, attending community meetings, and engaging in collective issue initiation by 0.306 (95% CI: 0.159–0.454), 0.616 (95% CI: 0.409–0.823), and 0.430 (95% CI: 0.268–0.591) standard deviations respectively. These patterns may appear counter-intuitive, yet they are consistent with wealth dynamics in Africa, where formal housing represents a disproportionately large share of household assets. Consequently, wildfires impose greater economic losses on households living in formal structures, generating stronger incentives for political action.

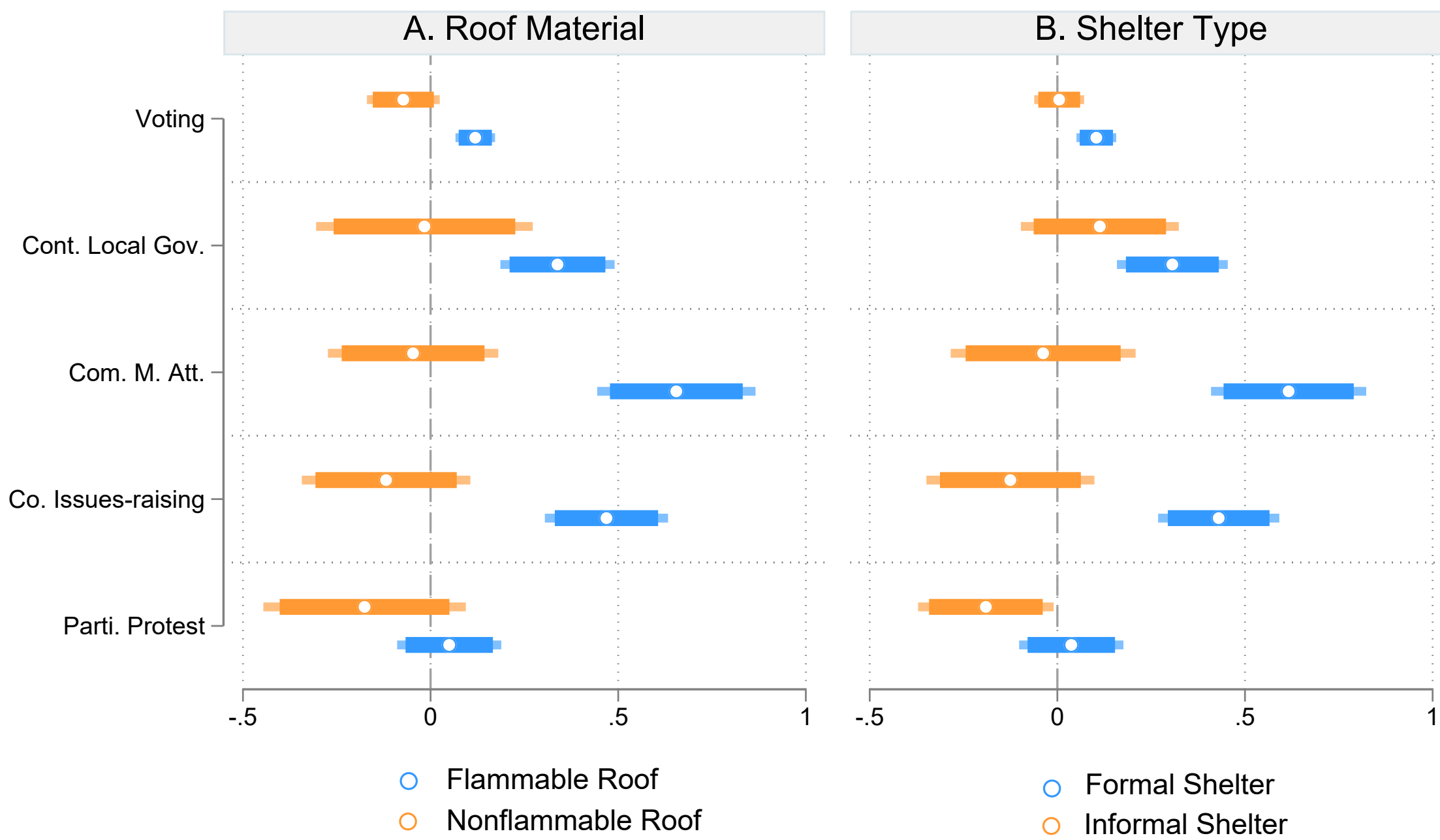

Figure 6: Heterogeneity Analysis: House Types and Roof Materials

We again appreciate your thoughtful feedback and we think the revisions have addressed the concerns raised. We are glad to incorporate any additional suggestions you may have to further improve the manuscript.

2. Reviewer #2

RC: ***This paper addresses an important question: how exposure to extreme climate events affects political participation, by examining the effect of wildfires on a range of political engagement indicators across African countries. It relies on survey data from Afrobarometer, which allows the authors to account for a wide range of individual-level characteristics. While the authors' efforts to assemble such a massive dataset are commendable, and the potential for making important contributions to the scientific debate is clear, I have two central concerns with the study.***

AR: We thank you for highlighting the importance of the research question and for recognizing the effort involved in assembling the dataset. Below, we address the central concerns you raised in detail, and we have revised the manuscript accordingly.

2.1. Comment

RC: ***The paper does not employ a research design that allows it to establish a causal link between exposure to wildfires and political behaviors. The analysis is based on repeated cross-sectional survey data, which are analyzed with a model where the unit of analysis is the individual. The treatment is defined by respondents' location within 5 miles of a wildfire during the 24 months preceding the survey. The control group includes respondents residing in areas between 7 and 20 miles from the nearest wildfire. This choice is justified as a way to "reduce measurement error" (p. 4), but I suppose the intention is rather to mitigate concerns about confounders. The model further includes fixed effects by year and country.***

AR: We thank you for this helpful suggestion. We excluded respondents residing in areas between 5 and 7 miles from the nearest wildfire to reduce geocoding-related error and to avoid borderline cases that may introduce confounding between exposed and non-exposed areas. We have revised the relevant sentences in the manuscript, which we reproduce below for ease of reference.

Paragraph 3 on Page 6:

> To reduce the potential confounders caused by geocoding-related errors and ensure a clearer separation between exposed and non-exposed areas, the control group comprises respondents residing in villages of 7 to 20 miles to the nearest wildfires [31].

Paragraph 2 on Page 22:

> We exclude the wildfire that occurred at a distance of 5 to 7 miles from a respondent's location to limit imprecision arising from village geocoding or wildfire coordinates and to reduce potential overlap between treated and control areas that could introduce confounding factors [31].

2.2. Comment

RC: ***The key point is that exposure to a wildfire is not a random event. Wildfires likely happen in areas having certain characteristics. The use of country-level fixed effects cannot account for the subnational differences that are likely to lead to both the eruption of wildfires and differences in political participation. The evidence from Table 2, which is now presented as evidence on the mechanism, may as well be (and***

most likely is) evidence of such a selection problem, because it does not present evidence on changes in material deprivation following the fire. It rather shows that areas closer to wildfires are systematically different from control areas.

AR: We thank you for the comments. To address the reviewer's comment and also based on the suggestions from other reviewers, we adopt an IV approach to solve the potential endogeneity. Specifically, we construct wildfire-risk instruments by interacting lagged land-cover flammability with exogenous weather conditions measured two years prior to the interview (average temperature and precipitation), and by including average wind speed from the same period, while we also control for the direct effects of temperature, precipitation and land cover to meet the exclusion restriction. Under the IV models, our major conclusions still hold. Specifically, wildfire exposure significantly increases both institutionalized and non-institutionalized political participation, especially community meeting attendance, while the effects on frequency of protest participation turn out to be insignificant under the IV strategy. We would further address the mechanism issues related to Table 2 in our following reply to Comment 2.3. The following Table 1 and text present and illustrate the revised baseline results.

Paragraph 3 on Page 24 – Paragraph 5 on Page 25:

However, the OLS results of Equation (3) are difficult to interpret as a causal effect. Causal identification would require that, among respondents living within 20 miles of a wildfire in the two years preceding the survey, their distance to the wildfire is as good as random. This assumption is difficult to sustain because human activity and the occurrence of wildfires are often correlated, leading to an omitted variable bias [12]. For example, settlements located in more ecologically fragile areas may be both more fire-prone and more politically active as residents seek government assistance to cope with environmental stress. In such cases, the OLS would conflate the effect of wildfire exposure with underlying social or political conditions.

To address the potential endogeneity, we construct our instrumental variables, named as wildfire risk, by interacting exogenous weather conditions with lagged land-cover flammability. Specifically, we use average precipitation, average temperature, and average wind speed measured two years prior to the interview year ($Tem_{v(i),t-2}, Prep_{v(i),t-2}, Windspeed_{v(i),t-2}$), together with a dummy whether the land cover at the respondent's location was flammable in year three years earlier ($I(LC)_{v(i),t-3}$). Accordingly, the wildfire risk instrument vector is:

$$\mathbf{WildfireRisk}_{v(i),t-2} = \begin{bmatrix} Prep_{v(i),t-2} \cdot I(LC)_{v(i),t-3} \\ Tem_{v(i),t-2} \cdot I(LC)_{v(i),t-3} \\ Windspeed_{v(i),t-2} \end{bmatrix} \quad (4)$$

Our identification strategy relies on that temperature, precipitation, wind speed and land-cover characteristics are plausibly exogenous environmental determinants of wildfire occurrence. These factors shape the likelihood and intensity of wildfires [22, 12]. After controlling for baseline climatic conditions and socioeconomic factors, these environmental variables are not chosen by local communities.

To address the exclusion restriction, we adopt the following approach. By interacting temperature and precipitation with an indicator for whether local land cover is combustible, we isolate the specific channel through which climatic conditions affect political participation via their impact on wildfire risk.

We also control for temperature, precipitation, and land-cover characteristics in the estimation. These controls absorb any alternative pathways through which these meteorological variables might influence political participation. As a result, the residual variation in the interaction terms identifies exclusively the component of weather variation that operates through wildfire incidence [23, 12, 24, 25].

We further conduct a falsification test to verify the exclusion restriction by regressing a set of potential mechanism outcomes on the instrumental variables, while simultaneously controlling for wildfire exposure and the full set of baseline covariates. As reported in Table **??**, the estimated coefficients on the individual instruments are generally small and statistically weak across all specifications. Moreover, the joint Wald F-statistics testing the null hypothesis that the coefficients on the three instruments are jointly equal to zero are uniformly low. After applying Holm's step-down adjustment to account for multiple hypothesis testing across outcome groups, none of these joint tests remains statistically significant. Taken together, these results provide additional support for the exclusion restriction by failing to detect systematic associations between the instruments and the proposed mechanism variables, conditional on wildfire exposure and observed confounders.

The full model with the first stage is derived as:

$$\begin{cases} Y_{i,t} = \alpha + \beta_1 \cdot \widehat{Wildfire}_{v(i),t-2} + \beta_{\mathbf{2}} \cdot \mathbf{Control_{v(i),t}} + C_{c(i)} \times Year_t + \varepsilon_{i,t}, \\ Wildfire_{v(i),t-2} = \eta + \gamma_{\mathbf{1}} \cdot \mathbf{WildfireRisk}_{v(i),t-2} + \gamma_{\mathbf{2}} \cdot \mathbf{Control_{v(i),t}} + C_{c(i)} \times Year_t + \epsilon_{i,t} \end{cases} \tag{5}$$

where $\mathbf{WildfireRisk}_{v(i),t-2}$ is the instrumental variables of wildfire, calculated by Equation (4), and $\mathbf{Control_{v(i),t}}$ is the temperature, precipitation, and land-cover characteristics at year t when surveyed. By incorporating country-by-year fixed effects $C_{c(i)} \times Year_t$, we account for unobserved county-specific time shocks such as war, structural economic change, and large-scale social unrest that could otherwise bias our estimates. In the robustness checks, we further control for respondent characteristics, including urban residency status, gender, age, and access to personal information channels. We also adopt alternative model specifications including ordered probit model and ordered instrumented probit model to test the robustness of the estimation.

Paragraph 2 on Page 8 – Paragraph 3 on Page 8:

To mitigate this potential endogeneity, we construct an instrumental variable (IV) for wildfire proximity using meteorological and land cover data. Research demonstrates that exogenous climate factors, specifically temperature, precipitation, wind speed, and land cover, are key determinants of wildfire incidence [22, 12]. We thus construct a wildfire risk index instrumented for the wildfire exposure index by interacting the annual average temperature and precipitation with lagged land-cover flammability, and wind speed (As calculated by Equation (4)). Concurrently, in the Two-Stage Least Squares (2SLS) estimation, we additionally control for the direct effects of these three natural environment indicators. Under this specification, our identification assumption is that: after controlling for country-year fixed effects, temperature, precipitation, and land cover combustibility, the aforementioned interaction terms and wind speed influence respondent political participation solely through their impact on the distance of the wildfire from the respondent's village. This constructive logic is analogous to that employed in a series of related studies.[25, 24]

The 2SLS estimations presented in Panel B of Table 1 indicate that wildfire exposure increases both

institutionalized and non-institutionalized forms of political participation, ranging from voting to collective-issue raising, but not protest, which is consistent with our endogeneity analysis. Compared to individuals living at a distance of 7–20 miles, those residing within 5 miles of recent wildfires are significantly more engaged in a variety of civic activities. Compared to individuals who live farther away from the fire, those who live closer are approximately 7.8% (95% confidence intervals (CIs): 0.038–0.118) more likely to vote. Proximity to a wildfire increases citizens' reported frequency of contacting local councilors by 22.7% (95%CI: 0.116–0.339) of the standard deviations (S.D. = 0.89), which also boosts community meetings attendance by 46.6% (95%CI: 0.331–0.601) of the overall variation (S.D. = 1.31), and raises joint issues-setting by 27.0% (95% CI: 0.160–0.381) of the overall variation (S.D. = 1.29). In contrast, the effect on protest participation is insignificant. Protests may involve greater personal risk, such as potential confrontation with authorities or social backlash, which raises the threshold for participation compared to low-cost actions like voting or attending community meetings. As Table 1 illustrates, the OLS estimates are generally smaller, yet the significance and signs of the coefficients remain unchanged compared with the 2SLS estimates. We take the IV strategy as our preferred estimation.

Table 1: Baseline Effects of Wildfire Exposure on Political Participation

Dept. Vars.	Institutionalized Form			Non-institutionalized Form	
	Voting	Contacting local govt.	Attending com. meetings	Collective issue-raising	Protest
	(1)	(2)	(3)	(4)	(5)
Panel A: OLS Estimation					
Wildfire	0.008*	0.052***	0.084***	0.061***	0.019
	(0.0046)	(0.0147)	(0.0181)	(0.0155)	(0.0119)
Observations	69,940	83,206	83,206	83,206	83,206
Panel B: 2SLS Estimation					
Wildfire	0.078***	0.227***	0.466***	0.270***	0.073
	(0.0204)	(0.0567)	(0.0687)	(0.0563)	(0.0519)
Observations	69,940	83,206	83,206	83,206	83,206
Country-by-Year FE	Yes	Yes	Yes	Yes	Yes
Spatial Cut-off (miles)	20	20	20	20	20
Temporal Cut-off (months)	24	24	24	24	24

Note: Panel A reports the OLS estimation results by Equation (3), panel B reports the 2SLS estimation results based on Equation (5). We instrument $Wildfire_{v(i),t-2}$ by $\mathbf{WildfireRisk}_{v(i),t-2}$, constructed by climate conditions in the last two years. The variable *Wildfire* is a binary indicator equal to one if a wildfire occurred within 5 miles of the respondent's village in two years preceding the survey, and zero if the nearest wildfire occurred between 7 miles and 20 miles during the same period. The non-binary dependent variables, including contacting local government officials, attending community meetings, collective issue-raising, and participation in protests, are standardized before estimation. All specifications include country-by-year fixed effects to account for unobserved heterogeneity and national-level temporal trends. Robust standard errors are clustered at the subnational level. *** p<0.01, ** p<0.05, * p<0.1.

2.3. Comment

RC: ***The instrumental variable approach used in Table 2 is also problematic. The models assess the effect of deprivation on political mobilization by instrumenting deprivation with exposure to wildfires. This is presented as a strategy to "address potential endogeneity" (p. 6). However, what is missing is a discussion of the assumptions needed for causal identification. To be valid, an instrumental variable needs to meet the exclusion restriction: wildfires should affect political participation only through material deprivation. This is hard to prove.***

AR: Thank you for raising this comment. In the revised manuscript and in light of the concerns regarding model identification, we now adopt the instrumental variables (IV) specification as our primary estimation strategy. Further details are provided in our responses to Comments 2.2.

We agree with the reviewer that the earlier IV specification, which used wildfires as an instrument for material deprivation, relied on a strong exclusion restriction assumption that may be difficult to justify. This specification has therefore been removed. The revised analysis treats wildfire exposure as the endogenous variable, instrumented using exogenous weather conditions and land-cover flammability. These factors affect the likelihood of wildfires but are not plausibly linked to political participation through other channels.

We have also revised the mechanism analysis by treating material deprivation and perceptions of government performance as dependent variables to assess whether these channels respond to wildfire exposure. While the main conclusions remain unchanged (e.g. wildfire exposure does not appear to increase conflict or shift environmental or political attitudes), the revised analysis indicates that evaluations of government performance represent an additional mechanism beyond material deprivation. Relevant text and tables from the updated manuscript are provided below.

Paragraph 2 on Page 9 – Paragraph 5 on Page 10:

> Experiencing climate-related shocks can undermine individuals' living conditions and lead to economic losses [32, 33, 34], which may exacerbate social tensions,highlight the necessity of government aid [11] and in some cases trigger conflict and crime [35, 36, 37], thereby influencing political participation. Direct exposure may also enhance awareness of climate risks [6, 38], potentially increasing environmental concern and, in turn, the willingness to participate in collective action.
>
> To explore these channels, we examine these potential mechanisms, including material deprivations, perceptions of government performance, conflict exposure, and public concern by regressing each mechanism variable on wildfire exposure using the IV specification in Equation (5).
>
> As shown in Panel A of Table 2, individuals living within 5 miles of recent wildfires are significantly more likely to experience medicine, water and income deprivations than those farther away (Columns (1)–(4)). Notably, wildfire exposure also increases respondents' favourable evaluations of local councillors' performance (Column (5) of Panel A). This pattern is consistent with greater demand for public goods and local government responsiveness following climate-related shocks [8].
>
> We find that wildfire exposure reduces the likelihood of conflict and physical attack occurrence (Columns (6) and (7) of Panel B). Although this may appear counter-intuitive, most existing evidence links conflict to slower-moving climate pressures that reshape economic opportunities and resource competition [35]. Wildfires, by contrast, are episodic and destructive events, more akin to sudden shocks such as earthquakes, which have been shown to reduce conflict and foster cooperation [39]. Our baseline results also align with this logic: wildfire exposure increases participation in community-level

activities but does not affect protest involvement.

By contrast, we find no evidence that wildfire exposure increases general public concern (Column (9) and (10) of Panel B in Table 2). We further examine whether wildfires influence individuals' concern about environmental risks.[a] Column (8) of Panel B shows no significance for individuals who express concern about these environmental issues following wildfire exposure, compared to those farther from wildfire zones. These findings are consistent with the relatively low levels of climate literacy documented in Africa [40], which may limit the extent to which climate shocks translate into heightened environmental concern.

In conclusion, the results in Table 2 show that wildfire exposure increases political participation primarily through the wildfire-caused material deprivations and government reactions, rather than abstract public concerns or environmental concerns.

[a]We only leverage data from Afrobarometer Survey Round 6 here since only this round lists all natural risks in the questionnaire.

Table 2: Mechanism Analysis

Panel A: Material Deprivation and Government Performance					
	Material Deprivation				Percep. Local Govt.
Dep. Vars.	Without Food	Without Income	Without Medics	Without Water	Local Govt. Perf.
	(1)	(2)	(3)	(4)	(5)
Wildfire	0.0360	0.194***	0.281***	0.203**	0.218***
	(0.0812)	(0.0677)	(0.0763)	(0.0952)	(0.0689)
Observations	83,061	80,569	82,868	83,086	73,669
Panel B: Conflict,Crime and Concern					
	Conflict and Crime		Environmental / Political Concern		
Dep. Vars.	Conflict Count	Being Attacked	Env. Concern	Pub. Aff. Int.	Pol. Discussion
	(6)	(7)	(8)	(9)	(10)
Wildfire	-0.180**	-0.053*	-0.0120	0.0505	-0.0618
	(0.0700)	(0.0301)	(0.0105)	(0.0553)	(0.0474)
Observations	71,182	82,028	26,206	82,318	82,368
Country-by-Year FE	Yes	Yes	Yes	Yes	Yes
Spatial Cut-off (miles)	20	20	20	20	20
Temporal Cut-off (months)	24	24	24	24	24

Note: This table presents mechanism analysis using different outcome variables instrumented by climate conditions in the last 3 years. All non-binary dependent variables are standardised before estimation. The variable *Wildfire* is a binary indicator equal to one if a wildfire occurred within 5 miles of the respondent's village in two years preceding the survey, and zero if the nearest wildfire occurred between 7 miles and 20 miles during the same period. All specifications include country-by-year fixed effects to account for unobserved heterogeneity and national-level temporal trends. Robust standard errors are clustered at the subnational level and reported in parentheses. *** $p<0.01$, ** $p<0.05$, * $p<0.1$.

2.4. Comment

RC: ***Research in political science has so far developed more appropriate techniques to address these kinds of questions. One example is Pianta and Rettl 2025 (forthcoming in the American Journal of Political Science, not cited). They examine the effect of wildfires in Brazil on support for green policies. They adopt a range of strategies to assess causality, including difference-in-differences and (well-justified) instrumental variables. My recommendation for the present study is to move the unit of analysis from the individual to the smallest subnational unit at which Afrobarometer is representative and implement a staggered difference-in-differences design. This would allow the authors to test pre-treatment trends and provide evidence of a causal linkage.***

AR: Thank you for this valuable suggestion. We agree with the reviewer that the two-way fixed effects specification may generate misleading causal inferences. The difference-in-difference design by Pianta and Rettl [12] offers an important framework for establishing causal effects in settings with clear subnational treatment assignment and repeated observations at the same geographic units.

In our case, however, Afrobarometer's sampling design makes this approach difficult to implement. The survey is not structured as a panel at the subnational level, and many clusters are sampled only once across rounds. And collapsing the data to larger geographic units would reduce the individual-level heterogeneity that is central to our research question.

In light of these constraints, and to address the reviewer's concern about selection and endogeneity, we adopt an instrumental variable approach as our primary identification strategy. As detailed in the response to Comment 2.2, following Pianta and Rettl's method [12], this strategy uses the exogenous variation in weather conditions and land-cover flammability as instruments to identify the effect of wildfire exposure and allows us to retain the individual-level richness of the Afrobarometer data. The revised manuscript presents the main results based on this IV specification. For brevity, we refer the reader to our response to Comment 2.2 for the revised contents.

2.5. Comment

RC: ***The paper currently engages too superficially with prior political science research on extreme weather events. Key recent publications are not included (e.g., Pianta and Rettl AJPS 2025; Hilbig and Riaz JOP 2024; Cremaschi and Stanig JOP 2025). The result is that the paper does not clearly highlight that, even in places where climate literacy is high, exposure to extreme weather events does not necessarily translate into higher environmental concerns and pro-environment mobilization. Preoccupation with the material consequences of disasters has already been identified as a key channel that can nullify the pro-environmental effects of exposure to extreme weather events. Furthermore, these papers also highlight how the response by incumbent politicians is a key channel that can shape the effect of extreme weather events on political behavior. This key aspect is not mentioned in the current draft paper and should be integrated into the theoretical framework. I would recommend checking these papers and recent references therein to provide a more accurate picture of the state of the literature and clarify the authors' contribution.***

AR: Thank you for raising this important point. Following the reviewer's comments, we have substantially expanded the theoretical framework and literature review to incorporate insights from recent studies in the revised manuscript, including Pianta and Rettl (2025), Hilbig and Riaz (2024), and Cremaschi and Stanig (2025). Recent political science research has recognized that the impact of exposure to extreme climate events on political participation and environmental support is complex, and that material loss may be a key factor that could mitigate pro-environment concerns. We also observe that current research focuses on the conditions under which exposure to extreme weather disasters increases public support for environmental

protection. We have now explicitly integrated these mechanisms into our theoretical framework and used them to motivate our focus on material deprivation and government responsiveness in the African context.

We have also clarified our contribution. Beyond examining the political consequences of climate-related disasters in low-income settings using individual-level data, we argue that this paper enriches the discussion on how exposure to weather disasters influences community-level participation. This helps extend the literature that primarily concentrates on electoral outcomes and environmental attitudes in advanced democracies, where institutional responsiveness and risk perceptions differ markedly from those in African political contexts.

Below we present the theoretical framework and contribution in the revised manuscript for ease of reference.

Paragraph 2 on Page 3 – Paragraph 1 on Page 4:

Understanding how climate-induced disasters shape political participation in Africa requires situating the analysis in the continent's unique institutional and political realities. Existing research on climate extremes has mainly examined electoral behaviour and environmental attitudes in developed democracies [5, 6, 7, 8, 9], whereas political engagement in Africa often follows a more instrumental logic: citizens assess institutions primarily by their ability to deliver immediate, tangible benefits rather than ideological commitments [1, 3, 13]. This instrumental approach has significant implications for how citizens mobilize politically after climate disasters. Furthermore, a notable gap exists in the literature's focus on electoral participation, with insufficient attention given to community-level civic engagement—a gap especially relevant for Africa, where local participation often serves as the main avenue through which citizens influence governance and express demands [4, 2]. Unlike national elections, which are infrequent, community activities such as contacting local officials, attending meetings, and raising collective issues provide ongoing opportunities for citizens to seek redress for immediate concerns. In Africa, with around 70 % of the population under 30 and many communities facing material deprivation and weak institutional capacity, local governance mechanisms are vital channels for grass-roots democracy. The significance of community participation also varies across different African contexts, shaped by decentralization frameworks, ethnic compositions, and institutional capacities, with countries like South Africa, Kenya, Ghana and Rwanda developing distinct local governance innovations that establish formal channels for disaster-affected communities to articulate demands [14, 15, 16].

We propose a theoretical framework centered on material deprivation and government responsiveness as the primary mechanisms linking wildfire exposure to political participation in Africa, departing from models developed in Global North contexts where climate disasters increase environmental concern and generate support for climate-friendly policies [6, 7, 17, 18]. In settings characterized by limited climate literacy, immediate material scarcity, and instrumental orientations toward democracy, wildfires mobilize citizens primarily through disruption of daily life and creation of urgent needs for government response [12, 10, 11]. Climate-induced wildfires generate immediate material consequences, destruction of property, loss of agricultural assets, disruption of food and water access, creating pressing needs that cannot be addressed through individual coping strategies alone. Where democracy is valued instrumentally as a means to securing tangible benefits, material deprivation creates powerful incentives for political mobilization [1, 3]. Citizens engage in community-level political activities not primarily from ideological commitment to environmental protection, but as strategic responses to immediate threats to well-being. Our framework distinguishes between institutionalized participation (voting, contacting councilors, community meetings), representing low-cost activities within existing governance structures, and non-institutionalized participation (collective issue-raising, protests) involving higher

costs but necessary when institutionalized channels prove unresponsive [19]. Political responses vary systematically across populations based on factors affecting both the severity of material losses and the capacity for mobilization, including climate vulnerability, ethnic political status, information accessibility, and socioeconomic resources [20, 21]. By centering community-level participation and theorizing material deprivation and government's bundled reactions as primary mechanisms, we extend understanding of how citizens in the Global South respond to climate extremes through diverse forms of civic engagement, illuminate essential differences between political responses in developed versus developing countries, and provide insights into which communities can mobilize effectively to secure disaster assistance.

Paragraph 4 on Page 5:

Furthermore, our study also speaks to recent debates on the heterogeneous contexts in which extreme climate events affect political participation. While many studies find that climate disasters generally increase political engagement and support for climate or green policies [41, 42]. Emerging evidence shows that these effects can weaken or even reverse depending on local economic structures and political incentives [10, 11]. Specifically, in Amazon, large-scale wildfires function as de facto land-clearing mechanisms, leading affected groups to oppose green policies rather than support them [12]. Although our data do not allow us to examine African citizens' views on green policies directly, the heterogeneity analysis provides complementary insights. Wildfire exposure increases community-level participation among both agricultural and non-agricultural workers, but the differences between the two groups are modest. This likely reflects the African context, where agricultural burning occurs on a small, household scale and farmers do not benefit economically from large, landscape-scale wildfires [28].

We again appreciate your thoughtful feedback and hope the revisions have addressed the concerns raised. We would be glad to incorporate any additional suggestions to further improve the manuscript.

3. Reviewer #3

3.1. Comment

RC: ***This article intervenes in growing debates about the relationship between extreme weather or disaster exposure and political attitudes, linking wildfire exposure in Africa to increased political participation. I think topically this is quite interesting, and the geo-located Afrobarometer data that the authors use here is a dataset I view as an underutilized as a resource in climate opinion scholarship. Efforts like this one to merge this expansive dataset with geophysical data are thus welcome. However, I am not yet persuaded that the article sustains it's key claims, and would need the authors to much more fulsomely explicate and justify their inferential strategy before seeing this research published in a venue such as Nature Communications.***

AR: Thank you for the thoughtful comment. We appreciate your recognition of the study's potential and have substantially revised the manuscript to clarify and strengthen our identification strategy, as detailed below.

3.2. Comment

RC: ***Here, my major concerns relate to the article's causal identification and, in particular, the minimal discussion provided to elaborate this identification despite the substantial causal language deployed throughout. First, the article's core specification presumes exogeneity of wildfire exposure. The robustness checks used here are, on balance, helpful and appropriately vary the way that the treatment is operationalized. Other specifications are reported, but I agree with the paper's decision to foreground a simple LPM model in this instance as the core reported specification.***

AR: We thank you for the comments. To address the reviewer's comment and together with comments raised by other reviewers, in the revised manuscript, we leverage average temperature, precipitation, wind speed in the present year and past two years and land cover in the 3rd year prior to the interview to construct instrumental variables for wildfire exposure (defined according to the distance between respondents and the nearest wildfire). We also control for the direct effects of temperature, precipitation, and land cover to help satisfy the exclusion restriction [12].

Paragraph 3 on Page 24 – Paragraph 4 on Page 25:

> However, the OLS results of Equation (3) are different to interpret as a causal effect. Causal identification would require that, among respondents living within 20 miles of a wildfire in the two years preceding the survey, their distance to the wildfire is as good as random. This assumption is difficult to sustain because human activity and the occurrence of wildfires are often correlated, leading to an omitted variable bias [12]. For example, settlements located in more ecologically fragile areas may be both more fire-prone and more politically active as residents seek government assistance to cope with environmental stress. In such cases, the OLS would conflate the effect of wildfire exposure with underlying social or political conditions.
>
> To address the potential endogeneity, we construct our instrumental variables, named as wildfire risk, by interacting exogenous weather conditions with lagged land-cover flammability. Specifically, we use average precipitation, average temperature, and average wind speed measured two years prior to the interview year ($Tem_{v(i),t-2}, Prep_{v(i),t-2}, Windspeed_{v(i),t-2}$), together with a dummy whether the land cover at the respondent's location was flammable in year three years earlier ($I(LC)_{v(i),t-3}$).

Accordingly, the wildfire risk instrument vector is:

$$\mathbf{WildfireRisk}_{v(i),t-2} = \begin{bmatrix} Prep_{v(i),t-2} \cdot I(LC)_{v(i),t-3} \\ Tem_{v(i),t-2} \cdot I(LC)_{v(i),t-3} \\ Windspeed_{v(i),t-2} \end{bmatrix} \tag{4}$$

Our identification strategy relies on that temperature, precipitation, wind speed and land-cover characteristics are plausibly exogenous environmental determinants of wildfire occurrence. These factors shape the likelihood and intensity of wildfires [22, 12]. After controlling for baseline climatic conditions and socioeconomic factors, these environmental variables are not chosen by local communities.

To address the exclusion restriction, we adopt the following approach. By interacting temperature and precipitation with an indicator for whether local land cover is combustible, we isolate the specific channel through which climatic conditions affect political participation via their impact on wildfire risk. We also control for temperature, precipitation, and land-cover characteristics in the estimation. These controls absorb any alternative pathways through which these meteorological variables might influence political participation. As a result, the residual variation in the interaction terms identifies exclusively the component of weather variation that operates through wildfire incidence [23, 12, 24, 25].

We further conduct a falsification test to verify the exclusion restriction by regressing a set of potential mechanism outcomes on the instrumental variables, while simultaneously controlling for wildfire exposure and the full set of baseline covariates. As reported in Table B2, the estimated coefficients on the individual instruments are generally small and statistically weak across all specifications. Moreover, the joint Wald F-statistics testing the null hypothesis that the coefficients on the three instruments are jointly equal to zero are uniformly low. After applying Holm's step-down adjustment to account for multiple hypothesis testing across outcome groups, none of these joint tests remains statistically significant. Taken together, these results provide additional support for the exclusion restriction by failing to detect systematic associations between the instruments and the proposed mechanism variables, conditional on wildfire exposure and observed confounders.

The full model with the first stage is derived as:

$$\begin{cases} Y_{i,t} = \alpha + \beta_1 \cdot \widehat{Wildfire}_{v(i),t-2} + \beta_\mathbf{2} \cdot \mathbf{Control_{v(i),t}} + C_{c(i)} \times Year_t + \varepsilon_{i,t}, \\ Wildfire_{v(i),t-2} = \eta + \gamma_\mathbf{1} \cdot \mathbf{WildfireRisk}_{v(i),t-2} + \gamma_\mathbf{2} \cdot \mathbf{Control_{v(i),t}} + C_{c(i)} \times Year_t + \epsilon_{i,t} \end{cases} \tag{5}$$

where $\mathbf{WildfireRisk}_{v(i),t-2}$ is the instrumental variables of wildfire, calculated by Equation (4), and $\mathbf{Control_{v(i),t}}$ is the temperature, precipitation, and land-cover characteristics at year t when surveyed. By incorporating country-by-year fixed effects $C_{c(i)} \times Year_t$, we account for unobserved county-specific time shocks such as war, structural economic change, and large-scale social unrest that could otherwise bias our estimates. In the robustness checks, we further control for respondent characteristics, including urban residency status, gender, age, and access to personal information channels. We also adopt alternative model specifications including ordered probit model and ordered instrumented probit model and ordered instrumented probit model to test the robustness of the estimation.

Under the IV models, our major conclusions still hold. Specifically, wildfire exposure significantly increases both institutionalized and non-institutionalized political participation, especially community meeting attendance, while the effects on frequency of protest participation turn out to be insignificant. Below we cite the revised baseline results for ease of reference.

Paragraph 3 on Page 8:

> The 2SLS estimations presented in Panel B of Table 1 indicate that wildfire exposure increases both institutionalized and non-institutionalized forms of political participation, ranging from voting to collective-issue raising, but not protest, which is consistent with our endogeneity analysis. Compared to individuals living at a distance of 7–20 miles, those residing within 5 miles of recent wildfires are significantly more engaged in a variety of civic activities. Compared to individuals who live farther away from the fire, those who live closer are approximately 7.8% (95% confidence intervals (CIs): 0.038–0.118) more likely to vote. Proximity to a wildfire increases citizens' reported frequency of contacting local councilors by 22.7% (95%CI: 0.116–0.339) of the standard deviations (S.D. = 0.89), which also boosts community meetings attendance by 46.6% (95%CI: 0.331–0.601) of the overall variation (S.D. = 1.31), and raises joint issues-setting by 27.0% (95% CI: 0.160–0.381) of the overall variation (S.D. = 1.29). In contrast, the effect on protest participation is insignificant. Protests may involve greater personal risk, such as potential confrontation with authorities or social backlash, which raises the threshold for participation compared to low-cost actions like voting or attending community meetings. As Table 1 illustrates, the OLS estimates are generally smaller, yet the significance and signs of the coefficients remain unchanged compared with the 2SLS estimates. We take the IV strategy as our preferred estimation.

Table 1: Baseline Effects of Wildfire Exposure on Political Participation

	Institutionalized Form			Non-institutionalized Form	
Dept. Vars.	Voting	Contacting local govt.	Attending com. meetings	Collective issue-raising	Protest
	(1)	(2)	(3)	(4)	(5)
Panel A: OLS Estimation					
Wildfire	0.008*	0.052***	0.084***	0.061***	0.019
	(0.0046)	(0.0147)	(0.0181)	(0.0155)	(0.0119)
Observations	69,940	83,206	83,206	83,206	83,206
Panel B: 2SLS Estimation					
Wildfire	0.078***	0.227***	0.466***	0.270***	0.073
	(0.0204)	(0.0567)	(0.0687)	(0.0563)	(0.0519)
Observations	69,940	83,206	83,206	83,206	83,206
Country-by-Year FE	Yes	Yes	Yes	Yes	Yes
Spatial Cut-off (miles)	20	20	20	20	20
Temporal Cut-off (months)	24	24	24	24	24

Note: Panel A reports the OLS estimation results by Equation (3), panel B reports the 2SLS estimation results based on Equation (5). We instrument $Wildfire_{v(i),t-2}$ by $\mathbf{WildfireRisk}_{v(i),t-2}$, constructed by climate conditions in the last two years. The variable *Wildfire* is a binary indicator equal to one if a wildfire occurred within 5 miles of the respondent's village in two years preceding the survey, and zero if the nearest wildfire occurred between 7 miles and 20 miles during the same period. The non-binary dependent variables, including contacting local government officials, attending community meetings, collective issue-raising, and participation in protests, are standardized before estimation. All specifications include country-by-year fixed effects to account for unobserved heterogeneity and national-level temporal trends. Robust standard errors are clustered at the subnational level. *** p<0.01, ** p<0.05, * p<0.1.

3.3. Comment

RC: ***What is less clear though is whether this exogeneity assumption itself is credible. The authors restrict their non-treated data to people living in villages between 7 and 20 miles away from a wildfire, something they ascribe to an effort to "reduce measurement error" (page 4). This did not quite make sense to me, as restricting the control group presumably would have been done in order to make more plausible the as-if random assignment of wildfires between treated and non-treated villages. But the authors make no particular effort to guide the reader in assessing the viability of this assumption. It is, of course, helpful that the authors run a model controlling for various potential confounders, but they do not report anywhere the relative balance/imbalance on these measures for the treatment vs. control groups. To the degree that the implicit exogeneity assumptions here hold, we should expect largely balanced results on this front, but the absence of such a table makes me suspect imbalance and, in turn, does moderate the degree to which I am persuaded by the key finding. We are offered little to no discussion of treatment assignment dynamics (e.g. where and how wildfires occur, and how their spatial extents might correlate with patterns of economic and political development).***

AR: We thank you for this helpful comment. We agree with the reviewer that the OLS results of Equation (3) are difficult to interpret as a causal effect. We now revise our baseline identification by the instrumental variable strategy. Specifically, we construct wildfire-risk instruments by interacting lagged land-cover flammability with exogenous weather conditions measured two years prior to the interview (average temperature and precipitation), and by including average wind speed from the same period. We also control for the direct effects of temperature, precipitation, and land cover to help satisfy the exclusion restriction [12].

Under the IV models, our main conclusions still hold. Specifically, wildfire exposure significantly increases both institutionalized and non-institutionalized political participation, especially community meeting attendance, whereas the effects on frequency of protest participation turn out to be insignificant. Details are provided in our response to Comment 3.2.

3.4. Comment

RC: ***This opaque empirical consideration then structures so much of the follow-up mechanism and heterogeneous effect work that follows. For instance, Section 2.3 is somewhat surprising relative to existing literatures, but without having a better sense of whether the underlying specification is credibly identified from a causal perspective, it is equally possible that this stability is merely an artifact of the self-reported variables as measured in the Afrobarometer survey. The same is true for the many heterogenous treatment effects subsequently probed. The concerns for the reader remain. For example, is the relationship between ethnic groups + participation structured by considerations beyond wildfire exposure? Could wildfire exposure simply be picking up some variable that is correlated with both exposure and socioeconomic issues, creating apparent correlations. The reader can't fully decide based on the evidence presented.***

AR: Thank you for this thoughtful comment. We agree with the reviewer that the credibility of the mechanism and heterogeneity analyses depends on whether the underlying wildfire-participation relationship is identified in a causally meaningful way. As our responses to Comments 3.2 and 3.3, we now treat wildfire exposure as an endogenous regressor and employ an instrumental-variables strategy based on exogenous weather conditions interacted with lagged land-cover flammability. This approach removes concerns that self-reported political behaviours or unobserved subnational characteristics drive the observed correlations, as the instruments rely solely on environmental factors that are unrelated to respondents' reporting behaviour.

Using the revised identification strategy, we re-estimate the mechanism analysis by treating material deprivation and perceptions of government performance as outcome variables. The results confirm that material

deprivation remains a key channel and additionally suggest that evaluations of government performance also respond to wildfire exposure. Importantly, this pattern is not attributable to self-report artifacts: the instruments isolate variation in wildfire exposure that is orthogonal to individual reporting tendencies or local socioeconomic conditions.

We also re-estimate all heterogeneity analyses under the IV specification to ensure consistency with this improved identification framework. For brevity, we summarize the mechanism results below and refer the reviewer to the revised manuscript for the full set of updated heterogeneity estimates.

Paragraph 2 on Page 8 – Paragraph 5 on Page 9:

> Experiencing climate-related shocks can undermine individuals' living conditions and lead to economic losses [32, 33, 34], which may exacerbate social tensions,highlight the necessity of government aid [11] and in some cases trigger conflict and crime [35, 36, 37], thereby influencing political participation. Direct exposure may also enhance awareness of climate risks [6, 38], potentially increasing environmental concern and, in turn, the willingness to participate in collective action.
>
> To explore these channels, we examine these potential mechanisms, including material deprivations, perceptions of government performance, conflict exposure, and public concern by regressing each mechanism variable on wildfire exposure using the IV specification in Equation (5).
>
> As shown in Panel A of Table 2, individuals living within 5 miles of recent wildfires are significantly more likely to experience medicine, water and income deprivations than those farther away (Columns (1)–(4)). Notably, wildfire exposure also increases respondents' favourable evaluations of local councillors' performance (Column (5) of Panel A). This pattern is consistent with greater demand for public goods and local government responsiveness following climate-related shocks [8].
>
> We find that wildfire exposure reduces the likelihood of conflict and physical attack occurrence (Columns (6) and (7) of Panel B). Although this may appear counter-intuitive, most existing evidence links conflict to slower-moving climate pressures that reshape economic opportunities and resource competition [35]. Wildfires, by contrast, are episodic and destructive events, more akin to sudden shocks such as earthquakes, which have been shown to reduce conflict and foster cooperation [39]. Our baseline results also align with this logic: wildfire exposure increases participation in community-level activities but does not affect protest involvement.
>
> By contrast, we find no evidence that wildfire exposure increases general public concern (Column (9) and (10) of Panel B in Table 2). We further examine whether wildfires influence individuals' concern about environmental risks.[a] Column (8) of Panel B shows no significance for individuals who express concern about these environmental issues following wildfire exposure, compared to those farther from wildfire zones. These findings are consistent with the relatively low levels of climate literacy documented in Africa [40], which may limit the extent to which climate shocks translate into heightened environmental concern.
>
> In conclusion, the results in Table 2 show that wildfire exposure increases political participation primarily through the wildfire-caused material deprivations and government reactions, rather than abstract public concerns or environmental concerns.
>
> [a]We only leverage data from Afrobarometer Survey Round 6 here since only this round lists all natural risks in the questionnaire.

Table 2: Mechanism Analysis

Panel A: Material Deprivation and Government Performance					
	Material Deprivation				Percep. Local Govt.
Dep. Vars.	Without Food	Without Income	Without Medics	Without Water	Local Govt. Perf.
	(1)	(2)	(3)	(4)	(5)
Wildfire	0.0360	0.194***	0.281***	0.203**	0.218***
	(0.0812)	(0.0677)	(0.0763)	(0.0952)	(0.0689)
Observations	83,061	80,569	82,868	83,086	73,669
Panel B: Conflict,Crime and Concern					
	Conflict and Crime		Environmental / Political Concern		
Dep. Vars.	Conflict Count	Being Attacked	Env. Concern	Pub. Aff. Int.	Pol. Discussion
	(6)	(7)	(8)	(9)	(10)
Wildfire	-0.180**	-0.053*	-0.0120	0.0505	-0.0618
	(0.0700)	(0.0301)	(0.0105)	(0.0553)	(0.0474)
Observations	71,182	82,028	26,206	82,318	82,368
Country-by-Year FE	Yes	Yes	Yes	Yes	Yes
Spatial Cut-off (miles)	20	20	20	20	20
Temporal Cut-off (months)	24	24	24	24	24

Note: This table presents mechanism analysis using different outcome variables instrumented by climate conditions in the last 3 years. All non-binary dependent variables are standardised before estimation. The variable *Wildfire* is a binary indicator equal to one if a wildfire occurred within 5 miles of the respondent's village in two years preceding the survey, and zero if the nearest wildfire occurred between 7 miles and 20 miles during the same period. All specifications include country-by-year fixed effects to account for unobserved heterogeneity and national-level temporal trends. Robust standard errors are clustered at the subnational level and reported in parentheses. *** $p<0.01$, ** $p<0.05$, * $p<0.1$.

3.5. Comment

RC: ***A second example of the inadequate elaboration of causal considerations is in the mechanism section and the presentation of a very ambiguous instrumental variable model. Noting the serious endogeneity concerns here, they propose an instrumental variable solution, but offer no real rationale for why and how their chosen instrument meet the necessary assumptions for the paper's analysis. (In particular, they do not discuss the necessary exclusion restrictions that must hold and, in fact, given their instrument is "first principal component of household shortages of essential goods, including water, food, medicine and cash, over the previous week when surveyed, calculated by PCA method" - I cannot follow why applying a PCA analysis to self-reported endogenous measures from the same survey as the outcome variable offers any causal leverage.) I therefore find unpersuasive the sustained claims of causality that accompany this section too.***

AR: Thank you for this comment. As noted in our response to the previous comment, we have removed the earlier IV specification and now rely on the revised IV strategy using exogenous weather and land-cover variables, with a full explanation of the exclusion restrictions provided above. Overall, we find that the main conclusions remain unchanged, the revised analysis indicates that evaluations of government performance represent an additional mechanism beyond material deprivation. Details are provided in our response to Comment 3.4.

3.6. Comment

RC: ***Finally, I would encourage the article to dialog more with several literatures in political science on related topics. First, there is a growing literature on crime victimization and political participation (including a Bateson American Political Science Review article from several years ago that also looked at within-Afrobarometer correlations on this topic). Second, since wildfire exposure is a bundled treatment with incumbent / government responses to that wildfire and its aftereffects, consideration of whether and how potential participation impacts are a function of the disaster itself or policy responses to it could be elaborated. This type of mechanism is not tested or discussed theoretically, with the focus being on reactions to wildfire exposure only, and not its bundled consequences.***

AR: Thank you for this helpful suggestion. In the revised manuscript, we incorporate insights from the literature on crime victimization and political participation, as well as research emphasizing that climate shocks often operate as bundled treatments involving both the disaster itself and government responses. Consistent with the literature, our updated mechanism analysis shows that evaluations of local government performance respond to wildfire exposure, indicating that government responsiveness constitutes an additional pathway alongside material deprivation. We have integrated this mechanism into the revised theoretical framework and clarified it in the introduction and mechanism sections. For ease of reference, the updated theoretical framework is presented below.

Below we present the theoretical framework and contribution in the revised manuscript for ease of reference.

Paragraph 2 on Page 3 – Paragraph 1 on Page 4:

> Understanding how climate-induced disasters shape political participation in Africa requires situating the analysis in the continent's unique institutional and political realities. Existing research on climate extremes has mainly examined electoral behaviour and environmental attitudes in developed democracies [5, 6, 7, 8, 9], whereas political engagement in Africa often follows a more instrumental logic: citizens assess institutions primarily by their ability to deliver immediate, tangible benefits rather than ideological commitments [1, 3, 13]. This instrumental approach has significant implications

for how citizens mobilize politically after climate disasters. Furthermore, a notable gap exists in the literature's focus on electoral participation, with insufficient attention given to community-level civic engagement—a gap especially relevant for Africa, where local participation often serves as the main avenue through which citizens influence governance and express demands [4, 2]. Unlike national elections, which are infrequent, community activities such as contacting local officials, attending meetings, and raising collective issues provide ongoing opportunities for citizens to seek redress for immediate concerns. In Africa, with around 70 % of the population under 30 and many communities facing material deprivation and weak institutional capacity, local governance mechanisms are vital channels for grass-roots democracy. The significance of community participation also varies across different African contexts, shaped by decentralization frameworks, ethnic compositions, and institutional capacities, with countries like South Africa, Kenya, Ghana and Rwanda developing distinct local governance innovations that establish formal channels for disaster-affected communities to articulate demands [14, 15, 16].

We propose a theoretical framework centered on material deprivation and government responsiveness as the primary mechanisms linking wildfire exposure to political participation in Africa, departing from models developed in Global North contexts where climate disasters increase environmental concern and generate support for climate-friendly policies [6, 7, 17, 18]. In settings characterized by limited climate literacy, immediate material scarcity, and instrumental orientations toward democracy, wildfires mobilize citizens primarily through disruption of daily life and creation of urgent needs for government response [12, 10, 11]. Climate-induced wildfires generate immediate material consequences, destruction of property, loss of agricultural assets, disruption of food and water access, creating pressing needs that cannot be addressed through individual coping strategies alone. Where democracy is valued instrumentally as a means to securing tangible benefits, material deprivation creates powerful incentives for political mobilization [1, 3]. Citizens engage in community-level political activities not primarily from ideological commitment to environmental protection, but as strategic responses to immediate threats to well-being. Our framework distinguishes between institutionalized participation (voting, contacting councilors, community meetings), representing low-cost activities within existing governance structures, and non-institutionalized participation (collective issue-raising, protests) involving higher costs but necessary when institutionalized channels prove unresponsive [19]. Political responses vary systematically across populations based on factors affecting both the severity of material losses and the capacity for mobilization, including climate vulnerability, ethnic political status, information accessibility, and socioeconomic resources [20, 21]. By centering community-level participation and theorizing material deprivation and government's bundled reactions as primary mechanisms, we extend understanding of how citizens in the Global South respond to climate extremes through diverse forms of civic engagement, illuminate essential differences between political responses in developed versus developing countries, and provide insights into which communities can mobilize effectively to secure disaster assistance.

Paragraph 4 on Page 5:

Furthermore, our study speaks to recent debates on the heterogeneous contexts in which extreme climate events affect political participation. While many studies find that climate disasters generally increase political engagement and support for climate or green policies [41, 42]. Emerging evidence shows that these effects can weaken or even reverse depending on local economic structures and political incentives [10, 11]. Specifically, in Amazon, large-scale wildfires function as de facto land-clearing

mechanisms, leading affected groups to oppose green policies rather than support them [12]. Although our data do not allow us to examine African citizens' views on green policies directly, the heterogeneity analysis provides complementary insights. Wildfire exposure increases community-level participation among both agricultural and non-agricultural workers, but the differences between the two groups are modest. This likely reflects the African context, where agricultural burning occurs on a small, household scale and farmers do not benefit economically from large, landscape-scale wildfires [28].

We again appreciate your thoughtful feedback and hope the revisions have addressed the concerns raised. We would be glad to incorporate additional suggestions to further improve the manuscript if any.

References

[1] Michael Bratton and Robert Mattes. Support for democracy in Africa: Intrinsic or instrumental? *British Journal of Political Science*, 31(03), 2001.

[2] Jesse Ribot. *Democratic decentralization of natural resources: Institutionalizing popular participation*. Washington DC: World Resources Institute, 2002.

[3] Robert Mattes and Michael Bratton. Learning about democracy in Africa: Awareness, performance, and experience. *American Journal of Political Science*, 51(1):192–217, 2007.

[4] John Gaventa and Gregory Barrett. Mapping the outcomes of citizen engagement. *World Development*, 40(12):2399–2410, 2012.

[5] David Castells-Quintana, Maria del Pilar Lopez-Uribe, and Thomas K. J. McDermott. Population displacement and urban conflict: Global evidence from more than 3300 flood events. *Journal of Development Economics*, 158, 2022.

[6] Roman Hoffmann, Raya Muttarak, Jonas Peisker, and Piero Stanig. Climate change experiences raise environmental concerns and promote green voting. *Nature Climate Change*, 12(2):148–155, 2022.

[7] Michael Coury. Climate risk and preferences over the size of government: Evidence from California wildfires. *The Review of Economics and Statistics*, 107(3):697–710, 2025.

[8] Sabrina B. Arias and Christopher W. Blair. In the eye of the storm: Hurricanes, climate migration, and climate attitudes. *American Political Science Review*, 118(4):1593–1613, 2024.

[9] Diogo Baerlocher, Renata Caldas, Francisco Cavalcanti, and Rodrigo Schneider. Natural disasters and voting behavior under authoritarian regimes: Evidence from the brazilian shrimp vote. *Journal of Economic Behavior & Organization*, 234, 2025.

[10] Hanno Hilbig and Sascha Riaz. Natural disasters and green party support. *The Journal of Politics*, 86(1):241–256, 2024.

[11] Simone Cremaschi and Piero Stanig. Voting and climate change: How an extreme weather event increased support for a radical-right incumbent in italy. *The Journal of Politics*, 87(4):000–000, 2025.

[12] Silvia Pianta and Paula Rettl. *Global Harms, Local Profits: How the Uneven Costs of Natural Disasters Affect Support for Green Political Platforms*. Harvard Business School, 2023.

[13] Martha Wilfahrt. Citizen response to local service provision: Emerging democratic accountability in decentralized west africa? *Electoral Studies*, 79:102498, 2022.

[14] Claire Bénit-Gbaffou. Party politics, civil society and local democracy–reflections from johannesburg. *Geoforum*, 43(2):178–189, 2012.

[15] Paul Smoke. Decentralisation in Africa: Goals, dimensions, myths and challenges. *Public Administration and Development: The International Journal of Management Research and Practice*, 23(1):7–16, 2003.

[16] Richard C Crook and James Manor. *Democracy and decentralisation in South Asia and West Africa: Participation, accountability and performance*. Cambridge University Press, 1998.

[17] Chie Hanaoka, Hitoshi Shigeoka, and Yasutora Watanabe. Do risk preferences change? Evidence from the great east Japan earthquake. *American Economic Journal: Applied Economics*, 10(2):298–330, 2018.

[18] Manja Gärtner, Johanna Mollerstrom, and David Seim. Individual risk preferences and the demand for redistribution. *Journal of Public Economics*, 153:49–55, 2017.

[19] Elke Zuern. *The politics of necessity: Community organizing and democracy in South Africa*. Univ of Wisconsin Press, 2011.

[20] Benn Eifert, Edward Miguel, and Daniel N Posner. Political competition and ethnic identification in Africa. *American journal of political science*, 54(2):494–510, 2010.

[21] Michelle Kuenzi and Gina Lambright. Party system institutionalization in 30 African countries. *Party Politics*, 7(4):437–468, 2001.

[22] W Matt Jolly, Mark A Cochrane, Patrick H Freeborn, Zachary A Holden, Timothy J Brown, Grant J Williamson, and David MJS Bowman. Climate-induced variations in global wildfire danger from 1979 to 2013. *Nature Communications*, 6(1):7537, 2015.

[23] John T Gasper and Andrew Reeves. Make it rain? retrospection and the attentive electorate in the context of natural disasters. *American journal of political science*, 55(2):340–355, 2011.

[24] Sarah Meier, Rob Elliott, and Eric Strobl. The regional economic impact of wildfires: Evidence from southern europe. *Journal of Environmental Economics and Management*, 118:102787, 2023.

[25] Mark Borgschulte, David Molitor, and Eric Yongchen Zou. Air pollution and the labor market: Evidence from wildfire smoke. *Review of Economics and Statistics*, 106(6):1558–1575, 2024.

[26] Adam F. A. Pellegrini, Anders Ahlström, Sarah E. Hobbie, Peter B. Reich, Lars P. Nieradzik, A. Carla Staver, Bryant C. Scharenbroch, Ari Jumpponen, William R. L. Anderegg, James T. Randerson, and Robert B. Jackson. Fire frequency drives decadal changes in soil carbon and nitrogen and ecosystem productivity. *Nature*, 553(7687):194–198, 2018.

[27] Benny Geys and Rune J Sørensen. Public sector employment and voter turnout. *American Political Science Review*, 116(1):367–373, 2022.

[28] Jakelyne S Bezerra, Víctor Arroyo-Rodríguez, Juan Manuel Dupuy-Rada, Inara R Leal, and Marcelo Tabarelli. Negative impact of slash-and-burn agriculture on the seed rain in a tropical dry forest. *Forest Ecology and Management*, 531:120821, 2023.

[29] Prem Sagar Chapagain, Tibendra Raj Banskota, Shobha Shrestha, Narendra Raj Khanal, Zhang Yili, Jianzhong Yan, Liu Linshan, Basanta Paudel, Suresh C Rai, Md Nurul Islam, et al. Studies on adaptive capacity to climate change: a synthesis of changing concepts, dimensions, and indicators. *Humanities and Social Sciences Communications*, 12(1):1–10, 2025.

[30] Guy Michaels, Dzhamilya Nigmatulina, Ferdinand Rauch, Tanner Regan, Neeraj Baruah, and Amanda Dahlstrand. Planning ahead for better neighborhoods: Long-run evidence from Tanzania. *Journal of Political Economy*, 129(7):2112–2156, 2021.

[31] Hannah Druckenmiller, Yanjun Liao, Sophie Pesek, Margaret Walls, and Shan Zhang. Removing development incentives in risky areas promotes climate adaptation. *Nature Climate Change*, 14(9):936–942, 2024.

[32] J. V. Henderson, A. Storeygard, and U. Deichmann. Has climate change driven urbanization in Africa? *Journal of Development Economics*, 124:60–82, 2017.

[33] Arash Modaresi Rad, John T Abatzoglou, Jason Kreitler, Mohammad Reza Alizadeh, Amir AghaKouchak, Nicholas Hudyma, Nicholas J Nauslar, and Mojtaba Sadegh. Human and infrastructure exposure to large wildfires in the United States. *Nature Sustainability*, 6(11):1343–1351, 2023.

[34] Florent Baarsch, Jessie R Granadillos, William Hare, Maria Knaus, Mario Krapp, Michiel Schaeffer, and Hermann Lotze-Campen. The impact of climate change on incomes and convergence in Africa. *World Development*, 126:104699, 2020.

[35] Eoin F. McGuirk and Nathan Nunn. Transhumant pastoralism, climate change, and conflict in Africa. *Review of Economic Studies*, 2024.

[36] Mariaflavia Harari and Eliana La Ferrara. Conflict, climate, and cells: A disaggregated analysis. *Review of Economics and Statistics*, 100(4):594–608, 2018.

[37] Regina Bateson. Crime victimization and political participation. *American Political Science Review*, 106(3):570–587, 2012.

[38] Marc Fabel, Matthias Flückiger, Markus Ludwig, Helmut Rainer, Maria Waldinger, and Sebastian Wichert. The relationship between the youth-led Fridays for future climate movement and voting, politician and media behaviour in Germany. *Nature Human Behaviour*, 9(3):481–495, 2025.

[39] Alexander De Juan, Jan Pierskalla, and Elisa Schwarz. Natural disasters, aid distribution, and social conflict–micro-level evidence from the 2015 earthquake in nepal. *World Development*, 126:104715, 2020.

[40] Nicholas P. Simpson, Talbot M. Andrews, Matthias Krönke, Christopher Lennard, Romaric C. Odoulami, Birgitt Ouweneel, Anna Steynor, and Christopher H. Trisos. Climate change literacy in Africa. *Nature Climate Change*, 11(11):937–944, 2021.

[41] Susanna Garside and Haoyu Zhai. If not now, when? climate disaster and the green vote following the 2021 germany floods. *Research & Politics*, 9(4):20531680221141523, 2022.

[42] Anton Kronborg, Frederik Hedegaard, Isak Klindt, and Clara Vandeweerdt. Do green parties in government benefit from natural catastrophes? how wildfires are linked to voting. *Electoral Studies*, 88:102749, 2024.

Authors' Response to Reviews of

Fires and Votes: How Wildfire Exposure Affects Africans' Political Participation

Nature Communications, `NCOMMS-25-68525A-Z`

RC: ***Reviewers' Comment***, AR: Authors' Response, ☐ Manuscript Text

We sincerely appreciate the editor and reviewers for their careful evaluation of our manuscript and constructive suggestions. These comments and suggestions have helped us to improve the instrumental-variable strategy, expand the discussion to the existing literature, and broaden the policy discussion. Below, we first offer a brief summary of the major concerns and how we address these concerns, followed by a point-by-point response.

- We strengthened the IV strategy by situating it more carefully in the wildfire literature, and providing discussion and validation of its credibility through improved exclusion-restriction discussion, first-stage and reduced-form evidence, falsification and over-identification tests, and additional robustness checks.
- We clarified the instrument's construction, timing, and data-generating logic in ***Methods*** section.
- We added a robustness check using a continuous measure of distance to wildfire and showed that the main findings remain consistent.
- We expanded the discussion to note that post-disaster participation may be linked to clientelistic mobilization through disaster relief, while interpreting this cautiously.

All changes are highlighted in BLUE in the revised manuscript. We believe the current manuscript is significantly improved, and hope that the revisions have addressed your concerns.

1. Reviewer 1

RC: ***I am satisfied that the authors have addressed my concerns, and this paper is close to being ready to publish. There are only two small points that I would suggest the authors consider:***

AR: We thank the reviewer for the positive assessment of the revised manuscript and for these helpful suggestions.

1.1. Comment 1

RC: ***I am pleased to see that the analysis of mechanism has been further developed. The mechanisms, as described, look very much like they involve clientelistic mobilization. It would make sense in the conclusion to comment on the normative implications of the use of disaster relief for clientelistic purposes.***

AR: We thank the reviewer for this helpful suggestion. We agree with the reviewer that our findings raise an important normative question about how disaster relief shapes post-disaster political mobilization. In the revised "Discussion" section, we add a brief paragraph noting that our findings, greater material deprivation, better evaluations of local government performance, and no increase in general political interest, are consistent

with a more instrumental and localized form of participation. We also note more cautiously that this pattern may be compatible with clientelistic mobilization through disaster relief. Given our data limitations, we cannot test this mechanism directly, and it remains an important question for future research.

Specifically, we added the following paragraph to the "Discussion" section:

Paragraph 2 on Page 21

> It is also worth noting that our findings, greater material deprivation, improved evaluations of local government performance, and no increase in general political interest, are consistent with a more instrumental and localized form of participation. This pattern may also be compatible with clientelistic mobilization through disaster relief[1, 2]. If so, increased participation after disaster exposure may reflect localized political responses to relief rather than broader civic engagement. However, our data do not allow us to test this mechanism directly. We therefore interpret this possibility with caution and leave it for future research.

1.2. Comment 2

RC: ***Page 3, penultimate line "while not concentrating" should be "while concentrating less", as there are papers on this topic.***

AR: Thank you. We have corrected this phrasing in the revised manuscript.

2. Reviewer 2

RC: ***I find that the paper has improved substantially. The revised empirical strategy based on an IV design is more convincing, and the theoretical discussion is now more thorough and better integrated with the empirical analysis. That said, I still have a few outstanding concerns regarding the empirical implementation that require clarification.***

AR: We thank the reviewer for the encouraging assessment and for these specific suggestions. These comments helped us improve both the transparency of the empirical strategy and the presentation of the IV results.

2.1. Comment 1

RC: ***The treatment variable (proximity to wildfires) is now operationalized as a dichotomous indicator distinguishing respondents who live close to a wildfire from those who live farther away. This choice introduces an arguably arbitrary modeling assumption. Using continuous distance from the wildfire would seem a more natural specification. In addition, I would like to see results that include respondents located between 7 and 20 miles from the nearest wildfire. Their exclusion is currently justified by the dichotomous operationalization of the treatment and the need to reduce measurement error. However, it would be reassuring to see robustness checks showing how sensitive the results are to this modeling choice. I would strongly encourage using a continuous measure of distance to wildfires—including all respondents within the relevant radius—as the main specification.***

AR: We thank the reviewer for this comment. The threshold-based treatment was intended to mitigate potential attenuation from geocoding error and to provide a transparent comparison between villages located very close to recent wildfires and those farther away. At the same time, we agree with the reviewer that it is important to show that the main findings are not driven by this specific modelling choice.

Following the reviewer's suggestions, we add robustness checks using a continuous measure of distance to the nearest wildfire over the 0 to 50 mile range, which also retained the observations between 7 and 20 miles from the nearest wildfires. These additional analyses yield consistent results. The coefficient of $Distance(miles)$ is statistically negative, indicating lower political participation as distance from the wildfire increases. In the main specification, we continue to use the dummy measure because it offers a more transparent and readily interpretable comparison between respondents who were closely exposed to wildfire and those located meaningfully farther away.

For ease of reference, we report the corresponding results below.

Paragraph 3 on Page 8

> Results are also robust to a range of sensitive tests, including alternative measurements of wildfire exposure that account for both frequency and size (Extended Table B2 and B3), varying distance binds to wildfires (Extended Table B4), continuous measurement of distance between wildfires and villages (Extended Table B5), models dropping samples with agricultural or built-in land cover (Extended Tables B6–B8) and models controlling for individual characteristics, information and transportation accessibility (Extended Table B9 and B10).

Table B5 on Page 46

Robustness Check: Continuous Measurements of Wildfire Distance

Dept. Vars.	Institutionalized Form			Non-institutionalized Form	
	Voting	Contacting local govt.	Attending com. meetings	Collective issue-raising	Protest
	(1)	(2)	(3)	(4)	(5)
**Panel A: 2SLS Estimation Results**					
Distance (miles)	-0.0041***	-0.0097*	-0.0254***	-0.0139**	-0.0026
	(0.0015)	(0.0058)	(0.0075)	(0.0061)	(0.0049)
Observations	97,084	83,206	83,206	83,206	83,206
**Panel B: OLS Estimation Results**					
Distance (miles)	-0.0001	-0.0012	-0.0037***	-0.0024**	-0.0004
	(0.0002)	(0.0008)	(0.0011)	(0.0010)	(0.0008)
Observations	97,084	83,206	83,206	83,206	83,206
Country-Year FE	Yes	Yes	Yes	Yes	Yes
Spatial Cut-off (miles)	50	50	50	50	50
Temporal Cut-off (months)	24	24	24	24	24

Note: This table reports the estimation results using a continuous measure of distance to the nearest wildfire over the 0 to 50 mile range as the exposure measurement. The non-binary dependent variables have been standardized before estimation. We report the coefficients from the estimations based on Equation (1). All specifications include county-by-year fixed effects to account for unobserved heterogeneity and national-level temporal trends. Robust standard errors are clustered at the subnational level, with their standardized form reported in parentheses. *** p<0.01, ** p<0.05, * p<0.1

2.2. Comment 2

RC: ***First-stage estimates and the corresponding F-statistics for the main models are neither reported nor discussed. This is a notable omission, given their central role in assessing instrument relevance and strength. In addition, reporting reduced-form estimates would also be considered good practice.***

AR: We thank the reviewer for the comments on this omission. In response, we include the first-stage estimates for the main models, together with the corresponding joint F-statistics in the revised manuscript. The F statistic is significant in the main specification, which rules out weak instrument problems. The reduced form estimates are reported in Table B12, while the first stage estimates are reported in Table B11. The table contents, and the corresponding revised contents in the manuscript are shown below.

Table 1 on Page 9

Baseline Effects of Wildfire Exposure on Political Participation

	Institutionalized Form			Non-institutionalized Form	
Dept. Vars.	Voting	Contacting local govt.	Attending com. meetings	Collective issue-raising	Protest
	(1)	(2)	(3)	(4)	(5)
Panel A: OLS Estimation					
Wildfire	0.0045	0.0433***	0.0668***	0.0486***	0.0127
	(0.0045)	(0.0145)	(0.0183)	(0.0156)	(0.0123)
Observations	69,940	83,206	83,206	83,206	83,206
Panel B: 2SLS Estimation					
Wildfire	0.0728**	0.1490*	0.3750***	0.2080**	0.0373
	(0.0290)	(0.0767)	(0.0975)	(0.0830)	(0.0683)
Observations	69,940	83,206	83,206	83,206	83,206
Hansen J Statistic	2.315	1.909	3.049	2.971	1.156
First Stage F Statistic	26.74	31.44	31.44	31.44	31.44
Country-by-Year FE	Yes	Yes	Yes	Yes	Yes
Spatial Cut-off (miles)	20	20	20	20	20
Temporal Cut-off (months)	24	24	24	24	24

Note: Panel A reports the OLS estimation results by Equation (3), panel B reports the 2SLS estimation results based on Equation (5). We instrument $Wildfire_{v(i),t-2}$ by $\mathbf{WildfireRisk}_{v(i),t-2}$, constructed by climate conditions in the last two years and land cover combustibility in the 3rd year prior to the interview year. The variable *Wildfire* is a binary indicator equal to one if a wildfire occurred within 5 miles of the respondent's village in two years preceding the survey, and zero if the nearest wildfire occurred between 7 miles and 20 miles during the same period. The non-binary dependent variables, including contacting local government officials, attending community meetings, collective issue-raising, and participation in protests, are standardized before estimation. All specifications include country-by-year fixed effects to account for unobserved heterogeneity and national-level temporal trends. Robust standard errors are clustered at the subnational level. *** p<0.01, ** p<0.05, * p<0.1.

Footnote 1 on Page 8

We also provide reduced form results of directly regressing dependent variables on IVs while also controlling for covariates as in the baseline results (Extended Table B12)

Table B12 on Page 52

Robustness Check: Reduced Estimation Results of Dependent Variables on IVs

Dept. Vars.	Institutionalized Form			Non-institutionalized Form	
	Voting	Contacting local govt.	Attending com. meetings	Collective issue-raising	Protest
	(1)	(2)	(3)	(4)	(5)
**Panel A: 2SLS Estimation Results**					
$Tem_{t-2} \cdot I(LC)_{t-3}$	-0.0004	0.0017	0.0164**	0.0123*	0.0065
	(0.0025)	(0.0055)	(0.0083)	(0.0071)	(0.0056)
$Wind_{t-2}$	-0.0162***	-0.0228	-0.0469***	-0.0216	0.0008
	(0.0060)	(0.0158)	(0.0177)	(0.0151)	(0.0119)
$Prep_{t-2} \cdot I(LC)_{t-3}$	-0.0216	0.0480*	0.0721*	0.0491	0.0043
	(0.0647)	(0.0268)	(0.0390)	(0.0375)	(0.0348)
Tem_{t-2}	0.0047***	0.0061	0.0185***	0.0144***	0.0120***
	(0.0018)	(0.0045)	(0.0060)	(0.0049)	(0.0045)
$Prep_{t-2}$	0.0387	0.0258	0.0458	0.0867***	0.0826***
	(0.0639)	(0.0216)	(0.0293)	(0.0252)	(0.0230)
$I(LC)_{t-3}$	0.0703	-0.2660	-0.6760**	-0.5270*	-0.1550
	(0.1400)	(0.2080)	(0.3240)	(0.2720)	(0.2840)
Observations	69, 940	83,206	83,206	83,206	83,206
Country-Year FE	Yes	Yes	Yes	Yes	Yes
Spatial Cut-off (miles)	50	50	50	50	50
Temporal Cut-off (months)	24	24	24	24	24

Note: This table reports the OLS estimation results of regressing dependent variables over IVs directly, while also controlling for covariates as in baseline specification (Equation 3). The non-binary dependent variables have been standardized before estimation. Annual precipitation ($Prep_{t-2}$) is log-transformed. And the land cover combustibility ($I(LC)_{t-3}$) is standardized by sample mean. All specifications include county-by-year fixed effects to account for unobserved heterogeneity and national-level temporal trends. Robust standard errors are clustered at the subnational level, with their standardized form reported in parentheses. *** p<0.01, ** p<0.05, * p<0.1

Paragraph 3 on Page 8 - Paragraph 1 on Page 9

First stage results (Extended Table B11) and some falsification tests (Extended Table B13) of the IV specification also do not challenge the validity of this IV setting.

Table B11 on Page 51

Robustness Check: First Stage of IV Estimation

Dept. Vars. = Wildfire	Sample	
	Election	Non-election
	(1)	(2)
$Tem_{t-2} \cdot I(LC)_{t-3}$	0.0242***	0.0197**
	(0.0088)	(0.0095)
$Wind_{t-2}$	-0.1610***	-0.1640***
	(0.0248)	(0.0201)
$Prep_{t-2} \cdot I(LC)_{t-3}$	0.6780**	0.0588
	(0.3190)	(0.0504)
Tem_{t-2}	0.0294***	0.0207**
	(0.0086)	(0.0089)
$Prep_{t-2}$	0.4070	0.0464
	(0.3060)	(0.0401)
$I(LC)_{t-3}$	-1.6420***	-0.6920*
	(0.5960)	(0.3800)
F-statistic	32.55	28.71
Observations	69,940	83,206
Country-Year FE	Yes	Yes
Spatial Cut-off (miles)	20	20
Temporal Cut-off (months)	24	24

Note: This table reports the first estimation results of the IV estimation reported in panel B of Table 1. Annual precipitation ($Prep_{t-2}$) is log-transformed. And the land cover combustibility ($I(LC)_{t-3}$) is standardized by sample mean. The *F Statistic* reports the joint F test results of excluded IVs. We report the coefficients from the estimations based on Equation (5). All specifications include county-by-year fixed effects to account for unobserved heterogeneity and national-level temporal trends. Robust standard errors are clustered at the subnational level, with their standardized form reported in parentheses. *** p<0.01, ** p<0.05, * p<0.1

2.3. Comment 3

RC: ***The instruments are measured well before the occurrence of the wildfire. Weather variables (precipitation, temperature, and wind speed) are measured two years prior to the interview, while land combustibility is measured three years before. It is unclear why this timing is chosen. If the IV strategy is intended to exploit exogenous variation in wildfire exposure, it seems that the relevant variation should be measured as close as possible to the event itself. For instance, Pianta and Rettl (2025) rely on weather measures from the week preceding the wildfire. Relatedly, I do not understand why land combustibility is operationalized as a binary indicator, given that it is likely available as a continuous index in the original data.***

AR: We thank the reviewer for this comment. To avoid potential misunderstanding, we further clarify the construction of the IV and the timing of each of its components in the revised manuscript.

The Afrobarometer survey asks respondents how often they engaged in specific political activities during the year preceding the interview, corresponding to the period from $T-1$ to T in Figure R1 below. Because the

survey does not record the exact dates of these activities, we measure wildfire exposure over the two years preceding the interview, that is, from $T-2$ to T. This is exactly why we cannot exploit short-term weather fluctuations as instrumental variables directly as Pianta and Rettl (2025) [3].

Consistent with this exposure window, the weather variables, including precipitation, temperature, and wind speed, are also measured over the period from $T-2$ to T, as they capture the time-varying conditions that directly affect wildfire occurrence. By contrast, land-cover combustibility is measured earlier, from $T-3$ to $T-2$, because land cover is a predetermined determinant of wildfire susceptibility but may itself be altered by recent fires. This timing structure therefore helps satisfy the relevance and exclusion requirements of the IV. The weather conditions are aligned with the wildfire exposure window and therefore predict wildfire risk, while the lagged land-cover combustibility measure is predetermined with respect to recent wildfire outcomes and political responses, reducing concerns that the instrument captures post-fire adjustments rather than exogenous susceptibility.

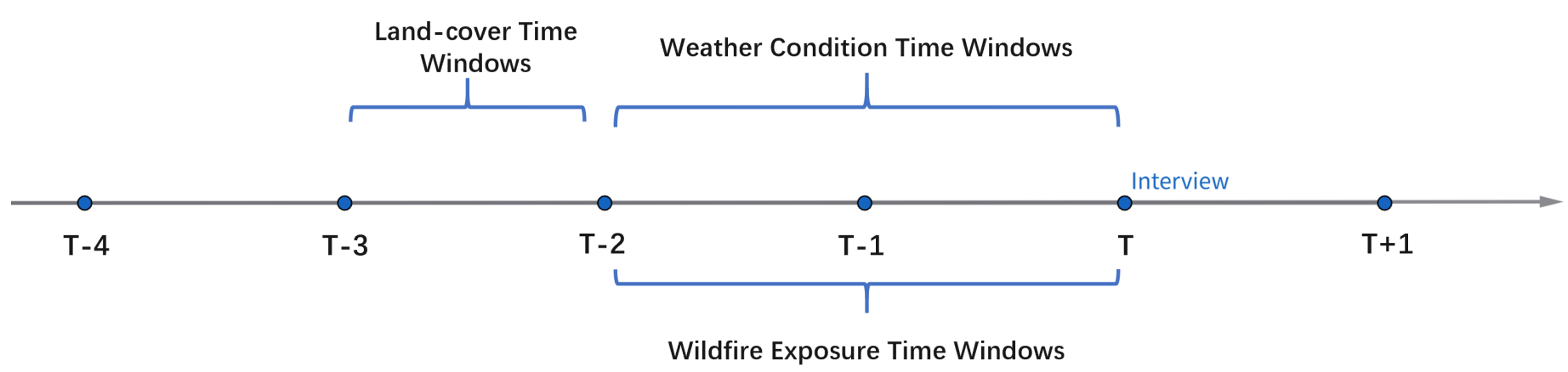

Figure R1. Time Windows of Instrumental Variable Construction

We also revise the main text in the manuscript, which is cited below for ease of reference.

Paragraph 6 on Page 24 – Paragraph 2 on Page 25

> However, the OLS estimates from Equation (3) are likely to be endogenous. Wildfire exposure is not randomly assigned across villages, and fire-prone locations may differ systematically in unobserved social, economic, and political characteristics that also affect political participation [3]. In this case, OLS would confound the effect of wildfire exposure with these underlying local conditions. Existing literature addresses this issue by exploiting natural variation in wildfire risk or ignition conditions. For example, Pianta and Rettl [3] instrument wildfire exposure with short-term weather fluctuations, noting that longer-term weather conditions may affect voting behaviour through channels other than fires. Umetani [4] instead uses lightning strikes as an instrument for wildfire exposure. Related work also shows that drought is an important predictor of large-scale fire activity [5]. However, this strategy is less feasible in our setting because comparable lightning data are not consistently available across our Africa-wide sample and study period.
>
> Because our outcomes measure political participation over the year preceding the survey, it is not appropriate to leverage short-term (e.g., one week before specific events) fluctuations of weather conditions as instrumental variables directly as Pianta and Rettl [3], while using weather or drought directly as instruments is less suitable in our setting. These climatic conditions may affect political behaviour through channels other than wildfire, including agricultural production, household income, and local economic stress [6], making the exclusion restriction less plausible when they are used alone as instruments. We therefore construct an interaction-based instrument that is better aligned with our cumulative wildfire exposure measure , as detailed in Figure 7. Specifically, we interact meteo-

rological conditions measured over the two years preceding the survey ($Tem_{v(i),t-2}$, $Prep_{v(i),t-2}$, and $Windspeed_{v(i),t-2}$) with a predetermined measure of land-cover flammability observed one year earlier ($I(LC)_{v(i),t-3}$). These factors jointly shape the likelihood and intensity of wildfires [7, 3]. This timing is intentional: weather is measured over the wildfire-exposure window to capture the conditions that generate fires, whereas land-cover flammability is measured earlier because it is a predetermined determinant of wildfire risk that may itself be altered by recent fires. In the estimation, we control separately for meteorological conditions and land-cover characteristics, so identification comes from the interaction-based component of weather variation in more combustible areas. These controls effectively absorb any alternative pathways through which these meteorological variables might influence political participation. As a result, the residual variation in the interaction terms is intended to isolate the component of weather variation that operates through wildfire incidence [8, 9, 10].

We also agree with the reviewer that the earlier discussion of land-cover combustibility was too brief. We have expanded the methods section to define the combustibility metric more carefully, explain how land-cover classes are mapped into combustible versus less combustible categories, and discuss why the baseline measure was initially coded as a binary indicator. It is not from continuous vegetation land cover measurement but rather from a categorical classification. In addition, we now provide robustness checks using alternative coding choices (by dropping samples with land cover combustible properties brought about by agricultural activities or urban development) for the combustibility component.

The explanation is cited here for ease of reference:

Paragraph 3 on Page 24

We also use land-cover data to measure flammability. As shown in Extended Table A6, bare land, permanent snow and ice, water bodies, and urban and built-up lands are classified as non-combustible, whereas forests, herbaceous areas, croplands, and shrublands are classified as combustible. The land cover combustibility dummy variable is re-centered by subtracting sample mean before applied in regression analysis. In robustness checks, we further exclude agricultural and built-up land-cover categories to reduce concerns that the interaction term captures agricultural or urban-related channels rather than wildfire risk. The land-cover data are sourced from MODIS through Google Earth Engine.

Table A6 on Page 41

Landcover Types and Corresponding Codes

**Landcover Type**	**Code**	**Explanation**
No data / Fill value	0	No data, used as fill value.
Bare land / Permanent snow or ice	1	Sand, rock, soil, or permanent snow/ice with less than 10% vegetation cover.
Permanent snow and ice	2	At least 60% of the area is covered by snow and ice for at least 10 months of the year.
Water bodies	3	At least 60% of the area is covered by permanent water bodies.
Urban and built-up lands	9	At least 30% of the area consists of impervious surfaces, including buildings, asphalt, and vehicles.
Dense forests	10	Tree cover exceeds 60%, with canopy height greater than 2 meters.
Open forests	20	Tree cover ranges from 10% to 60%, with canopy height greater than 2 meters.
Forest/cropland mosaics	25	Mosaics of small-scale cultivation (40%–60%) with more than 10% natural tree cover.
Natural herbaceous	30	Dominated by herbaceous annual plants shorter than 2 meters, with at least 10% cover.
Natural herbaceous/cropland mosaics	35	Mosaics of small-scale cultivation (40%–60%) mixed with natural shrub or herbaceous vegetation.
Herbaceous croplands	36	Dominated by herbaceous annual plants shorter than 2 meters, with at least 60% cover and cultivated fraction above 60%.
Shrublands	40	Shrub cover exceeds 60%, with vegetation height between 1 and 2 meters.

Notes: This table presents definition of land cover types and how they are coded in our research. The data is sourced from MODIS and processed by Google Earth Engine.

Paragraph 2 on Page 26

In the estimation, identification comes from variation in meteorological conditions that shifts wildfire risk differentially across areas with more versus less combustible land cover, conditional on the main effects of weather and land-cover characteristics. As shown in Extended Table A6, the land-cover categories include not only natural environments, such as ice and snow, sandy areas, water bodies, forests, and grasslands, but also built-up areas and agricultural zones. One possible concern is that some land-cover categories correspond to agricultural or built-up environments, so the interaction term could partly reflect channels other than wildfire risk. For example, agricultural land may be associated with local production conditions, while built-up land may be correlated with settlement intensity and related social or economic activity. To assess whether our results are driven by these alternative channels, we conduct three robustness checks by excluding observations in agricultural areas, built-up areas, and both agricultural and built-up areas. As reported in Extended Tables B6–B8, the results remain consistent with the baseline estimates.

Table B6 on Page 47

Robustness Check: Sample without Combustible Land Cover due to Agriculture

	Institutionalized Form			Non-institutionalized Form	
Dept. Vars.	Voting	Contacting local govt.	Attending com. meetings	Collective issue-raising	Protest
	(1)	(2)	(3)	(4)	(5)
Wildfire	0.0776**	0.1910**	0.4030***	0.2110**	0.0420
	(0.0313)	(0.0837)	(0.1080)	(0.0899)	(0.0765)
Observations	55,230	65,981	65,981	65,981	65,981
Country-Year FE	Yes	Yes	Yes	Yes	Yes
Spatial Cut-off (miles)	20	20	20	20	20
Temporal Cut-off (months)	24	24	24	24	24

Notes: This table reports the 2SLS estimation results of the same IV setting as in Equation (5) while without samples in which land cover combustibility comes from agricultural activities. $Wildfire$ is a binary indicator equal to one if a wildfire occurred within 5 miles of the respondent's village in two years preceding the survey, and zero if the nearest wildfire occurred between 7 miles and 20 miles during the same period. Non-binary dependent variables have been standardized before estimation. We report the coefficients from the estimations based on Equation (5). All specifications include county-by-year fixed effects to account for unobserved heterogeneity and national-level temporal trends. Robust standard errors reported in parentheses are clustered at the subnational level. *** p<0.01, ** p<0.05, * p<0.1.

Table B7 on Page 47

Robustness Check: Sample without Built-in Land Cover

Dept. Vars.	Institutionalized Form			Non-institutionalized Form	
	Voting	Contacting local govt.	Attending com. meetings	Collective issue-raising	Protest
	(1)	(2)	(3)	(4)	(5)
Wildfire	0.1180***	0.1630*	0.3120***	0.1660*	-0.0136
	(0.0413)	(0.0940)	(0.1090)	(0.0910)	(0.0713)
Observations	66,319	78,628	78,628	78,628	78,628
Country-Year FE	Yes	Yes	Yes	Yes	Yes
Spatial Cut-off (miles)	20	20	20	20	20
Temporal Cut-off (months)	24	24	24	24	24

Notes: This table reports the 2SLS estimation results of the IV setting dropping samples with built-in or urban land-cover. $Wildfire$ is a binary indicator equal to one if a wildfire occurred within 5 miles of the respondent's village in two years preceding the survey, and zero if the nearest wildfire occurred between 7 miles and 20 miles during the same period. Non-binary dependent variables have been standardized before estimation. We report the coefficients from the estimations based on Equation (5). All specifications include county-by-year fixed effects to account for unobserved heterogeneity and national-level temporal trends. Robust standard errors reported in parentheses are clustered at the subnational level. *** p<0.01, ** p<0.05, * p<0.1.

Table B8 on Page 48

Robustness Check: Sample without Built-in and Agricultural Land Cover

	Institutionalized Form			Non-institutionalized Form	
Dept. Vars.	Voting	Contacting local govt.	Attending com. meetings	Collective issue-raising	Protest
	(1)	(2)	(3)	(4)	(5)
Wildfire	0.1290***	0.2250**	0.3400**	0.1780	-0.0001
	(0.0485)	(0.1060)	(0.1320)	(0.1080)	(0.0823)
Observations	51,609	61,403	61,403	61,403	61,403
Country-Year FE	Yes	Yes	Yes	Yes	Yes
Spatial Cut-off (miles)	20	20	20	20	20
Temporal Cut-off (months)	24	24	24	24	24

Notes: This table reports the 2SLS estimation results of the IV setting dropping samples with built-in, urban or agricultural land-cover. $Wildfire$ is a binary indicator equal to one if a wildfire occurred within 5 miles of the respondent's village in two years preceding the survey, and zero if the nearest wildfire occurred between 7 miles and 20 miles during the same period. Non-binary dependent variables have been standardized before estimation. We report the coefficients from the estimations based on Equation (5). All specifications include county-by-year fixed effects to account for unobserved heterogeneity and national-level temporal trends. Robust standard errors reported in parentheses are clustered at the subnational level. *** $p<0.01$, ** $p<0.05$, * $p<0.1$.

3. Reviewer 3

3.1. Comment 1

RC: ***The authors here should be commended for the seriousness with which they took a very challenging set of initial reviews, including my own. Those reviews particularly pushed on whether the article could defend its causal claims, which is a large focus of the revisions offered here and the authors' response memo.***

I had mixed reactions to this revised manuscript. It is much cleaner and stronger than the initial version, focussing attention on the empirical claims that are most credible and offering a stronger theoretical and mechanistic account for these findings.

However, despite these improvements, one large reservation remains. The manuscript now pivots to (more appropriately) anchor it's identification strategy with an instrumental variables approach. Yet, the manuscript still does not provide enough context and information to allow for a reader to fully adjudicate this approach's credibility.

AR: We thank the reviewer for the thoughtful and constructive comments. In this revision, we have placed substantially more emphasis on explaining the instrument's construction, comparing the IV to related approaches in the literature, reporting first-stage and reduced-form evidence, and conducting more robustness checks to validate the exclusion restriction.

RC: ***This is not the first paper to instrument for wildfire exposure, and it would be good to review and contrast the papers' instrument with the other approaches taken in the literature. Some of these are similar in part (e.g. lagged drought) while others are different and less applicable to this specific dataset (e.g. lightning strike intensity).***

AR: We thank the reviewer for this suggestion. In response to this comment, we now situate our IV strategy more clearly in the wildfire literature. The revised manuscript explains that wildfire exposure is endogenous in OLS settings and discusses how previous studies use short-term weather fluctuations, lightning-based instruments, and drought-related fire risk to address this issue [3, 4, 5]. We also clarify why these alternatives are less suitable in our context. In particular, because our outcomes measure political participation over the year preceding the survey, using weather or drought directly as instruments would make the exclusion restriction less plausible.

We cite the revision here for ease of reference.

Paragraph 6 on Page 24 – Paragraph 2 on Page 25

> However, the OLS estimates from Equation (1) are likely to be endogenous. Wildfire exposure is not randomly assigned across villages, and fire-prone locations may differ systematically in unobserved social, economic, and political characteristics that also affect political participation [3]. In this case, OLS would confound the effect of wildfire exposure with these underlying local conditions. Existing literature addresses this issue by exploiting natural variation in wildfire risk or ignition conditions. For example, Pianta and Rettl [3] instrument wildfire exposure with short-term weather fluctuations, noting that longer-term weather conditions may affect voting behaviour through channels other than fires. Umetani [4] instead uses lightning strikes as an instrument for wildfire exposure. Related work also shows that drought is an important predictor of large-scale fire activity [5]. However, this strategy is less feasible in our setting because comparable lightning data are not consistently available across our Africa-wide sample and study period.

Because our outcomes measure political participation over the year preceding the survey, using weather or drought directly as instruments is less suitable in our setting. These climatic conditions may affect political behaviour through channels other than wildfire, including agricultural production, household income, and local economic stress [6], making the exclusion restriction less plausible when they are used alone as instruments.

3.2. Comment 2

RC: ***The paper needs to make its instrument more clear. In particular, the "flammability" metric that is used to construct the instrument is left underspecified. What does this metric measure in terms of land use? How is it measured? What is the data-generating process that shapes land-use flammability, and does the exclusion restriction plausibly hold with respect to that data-generating process? It may well do so! But the manuscript does not provide the detail or information necessary for that determination to be made, either by the paper or by interested readers. Even the methods section only provides a single line on this flammability metric and offers no real clarity on what it measures and how it is constructed.***

AR: We thank the reviewer for this comment. In the revised manuscript, we now clarify more explicitly what this measure captures, how it is constructed, and why it is measured prior to the exposure window. Specifically, in the data section, we explain that flammability is derived from MODIS land-cover data and classify land-cover categories into combustible and non-combustible surface types (Page 24, Paragraph 3; Extended Table A6). In the methods section, we further clarify that land-cover flammability is intended to capture predetermined landscape susceptibility to burning rather than realized wildfire itself, and that it is measured one year before the wildfire-exposure window because recent fires may alter land cover (Page 25, Paragraph 2).

We also address the reviewer's concern that land-cover flammability may proxy for other channels, such as agricultural production or urban-related human activity. To examine this possibility, we now report robustness checks that exclude agricultural areas, built-up areas, and both types of areas, and show that the results remain consistent with the baseline estimates (Page 26, Paragraph 2; Extended Tables B6–B8). In addition, we report falsification and over-identification tests, which provide further support for the exclusion restriction (Page 26, Paragraph 3). We believe these revisions make the construction and interpretation of the flammability measure substantially clearer and allow readers to better assess its role in the IV design.

We cite the revision here for ease of reference.

Paragraph 3 on Page 24

We also use land-cover data to measure flammability. As shown in Extended Table A6, bare land, permanent snow and ice, water bodies, and urban and built-up lands are classified as non-combustible, whereas forests, herbaceous areas, croplands, and shrublands are classified as combustible. In robustness checks, we further exclude agricultural and built-up land-cover categories to reduce concerns that the interaction term captures agricultural or urban-related channels rather than wildfire risk. The land-cover data are sourced from MODIS through Google Earth Engine.

Paragraph 2 on Page 26

In the estimation, identification comes from variation in meteorological conditions that shifts wildfire risk differentially across areas with more versus less combustible land cover, conditional on the main effects of weather and land-cover characteristics. As shown in Extended Table A6, the land-cover categories include not only natural environments, such as ice and snow, sandy areas, water bodies,

forests, and grasslands, but also built-up areas and agricultural zones. One possible concern is that some land-cover categories correspond to agricultural or built-up environments, so the interaction term could partly reflect channels other than wildfire risk. For example, agricultural land may be associated with local production conditions, while built-up land may be correlated with settlement intensity and related social or economic activity. To assess whether our results are driven by these alternative channels, we conduct three robustness checks by excluding observations in agricultural areas, built-up areas, and both agricultural and built-up areas. As reported in Extended Tables B6–B8, the results remain consistent with the baseline estimates.

Table A6 on Page 41

Landcover Types and Corresponding Codes

Landcover Type	Code	Explanation
No data / Fill value	0	No data, used as fill value.
Bare land / Permanent snow or ice	1	Sand, rock, soil, or permanent snow/ice with less than 10% vegetation cover.
Permanent snow and ice	2	At least 60% of the area is covered by snow and ice for at least 10 months of the year.
Water bodies	3	At least 60% of the area is covered by permanent water bodies.
Urban and built-up lands	9	At least 30% of the area consists of impervious surfaces, including buildings, asphalt, and vehicles.
Dense forests	10	Tree cover exceeds 60%, with canopy height greater than 2 meters.
Open forests	20	Tree cover ranges from 10% to 60%, with canopy height greater than 2 meters.
Forest/cropland mosaics	25	Mosaics of small-scale cultivation (40%–60%) with more than 10% natural tree cover.
Natural herbaceous	30	Dominated by herbaceous annual plants shorter than 2 meters, with at least 10% cover.
Natural herbaceous/cropland mosaics	35	Mosaics of small-scale cultivation (40%–60%) mixed with natural shrub or herbaceous vegetation.
Herbaceous croplands	36	Dominated by herbaceous annual plants shorter than 2 meters, with at least 60% cover and cultivated fraction above 60%.
Shrublands	40	Shrub cover exceeds 60%, with vegetation height between 1 and 2 meters.

Notes: This table presents definition of land cover types and how they are coded in our research. The data is sourced from MODIS and processed by Google Earth Engine.

Table B6 on Page 47

Robustness Check: Sample without Combustible Land Cover due to Agriculture

	Institutionalized Form			Non-institutionalized Form	
Dept. Vars.	Voting	Contacting local govt.	Attending com. meetings	Collective issue-raising	Protest
	(1)	(2)	(3)	(4)	(5)
Wildfire	0.0776**	0.1910**	0.4030***	0.2110**	0.0420
	(0.0313)	(0.0837)	(0.1080)	(0.0899)	(0.0765)
Observations	55,230	65,981	65,981	65,981	65,981
Country-Year FE	Yes	Yes	Yes	Yes	Yes
Spatial Cut-off (miles)	20	20	20	20	20
Temporal Cut-off (months)	24	24	24	24	24

Notes: This table reports the 2SLS estimation results of the same IV setting as in Equation 5, excluding samples in which land cover combustibility comes from agricultural activities. $Wildfire$ is a binary indicator equal to one if a wildfire occurred within 5 miles of the respondent's village in two years preceding the survey, and zero if the nearest wildfire occurred between 7 miles and 20 miles during the same period. Non-binary dependent variables have been standardized before estimation. We report the coefficients from the estimations based on Equation (5). All specifications include county-by-year fixed effects to account for unobserved heterogeneity and national-level temporal trends. Robust standard errors reported in parentheses are clustered at the subnational level. *** p<0.01, ** p<0.05, * p<0.1.

Table B7 on Page 47

Robustness Check: Sample without Built-in Land Cover

	Institutionalized Form			Non-institutionalized Form	
Dept. Vars.	Voting	Contacting local govt.	Attending com. meetings	Collective issue-raising	Protest
	(1)	(2)	(3)	(4)	(5)
Wildfire	0.1180***	0.1630*	0.3120***	0.1660*	-0.0136
	(0.0413)	(0.0940)	(0.1090)	(0.0910)	(0.0713)
Observations	66,319	78,628	78,628	78,628	78,628
Country-Year FE	Yes	Yes	Yes	Yes	Yes
Spatial Cut-off (miles)	20	20	20	20	20
Temporal Cut-off (months)	24	24	24	24	24

Notes: This table reports the 2SLS estimation results of the IV setting excluding samples with built-in or urban land-cover. $Wildfire$ is a binary indicator equal to one if a wildfire occurred within 5 miles of the respondent's village in two years preceding the survey, and zero if the nearest wildfire occurred between 7 miles and 20 miles during the same period. Non-binary dependent variables have been standardized before estimation. We report the coefficients from the estimations based on Equation (5). All specifications include county-by-year fixed effects to account for unobserved heterogeneity and national-level temporal trends. Robust standard errors reported in parentheses are clustered at the subnational level. *** p<0.01, ** p<0.05, * p<0.1.

Table B8 on Page 48

Robustness Check: Sample without Built-in and Agricultural Land Cover

Dept. Vars.	Institutionalized Form			Non-institutionalized Form	
	Voting	Contacting local govt.	Attending com. meetings	Collective issue-raising	Protest
	(1)	(2)	(3)	(4)	(5)
Wildfire	0.1290***	0.2250**	0.3400**	0.1780	-0.0001
	(0.0485)	(0.1060)	(0.1320)	(0.1080)	(0.0823)
Observations	51,609	61,403	61,403	61,403	61,403
Country-Year FE	Yes	Yes	Yes	Yes	Yes
Spatial Cut-off (miles)	20	20	20	20	20
Temporal Cut-off (months)	24	24	24	24	24

Notes: This table reports the 2SLS estimation results of the IV setting excluding samples with built-in, urban or agricultural land-cover. $Wildfire$ is a binary indicator equal to one if a wildfire occurred within 5 miles of the respondent's village in two years preceding the survey, and zero if the nearest wildfire occurred between 7 miles and 20 miles during the same period. Non-binary dependent variables have been standardized before estimation. We report the coefficients from the estimations based on Equation (5). All specifications include county-by-year fixed effects to account for unobserved heterogeneity and national-level temporal trends. Robust standard errors reported in parentheses are clustered at the subnational level. *** p<0.01, ** p<0.05, * p<0.1.

3.3. Comment 3

RC: ***The discussion of the exclusion restriction left me with some questions. It seems that the authors are concerned that the exclusion restriction does not hold in this case with simple measures of precipitation or temperature alone, and thus feel the need to interact these with the flammability measure. But this is very hard to follow for the reader, and I think requires more clear writing and justification. It is also possible that changing the measurement strategy for some of the temperature/precipitation measurements (to focus on long-term structural and lagged conditions) might be a better approach to making the exclusion restriction more credible.***

AR: We thank the reviewer for this comment. In the revised manuscript, we now clarify more explicitly why meteorological variables are not used directly as instruments and the reason that we instead adopt an interaction-based IV design. Specifically, we explain that, because our outcomes measure political participation over the year preceding the survey, weather or drought variables may affect political behaviour through channels other than wildfire, including agricultural production, household income, and local economic stress. This makes the exclusion restriction less plausible when these variables are used alone as instruments. We therefore construct an interaction-based instrument that combines meteorological conditions over the wildfire-exposure window with predetermined land-cover flammability measured prior to that window. In the estimation, we also control separately for meteorological conditions and land-cover characteristics, so identification comes from the interaction-based component of weather variation in more combustible areas. We further strengthen this discussion by reporting robustness checks that exclude agricultural and built-up

areas, as well as falsification and over-identification tests, all of which provide additional support for the exclusion restriction.

For ease of reference, we cite below the revised discussion of the interaction-based IV design. The robustness checks excluding agricultural and built-up areas are discussed in our response to Comment 2.

Paragraph 4 on Page 25

> Because our outcomes measure political participation over the year preceding the survey, it is not appropriate to leverage short-term (e.g., one week before specific events) fluctuations of weather conditions as instrumental variables directly as Pianta and Rettl [3], while using weather or drought directly as instruments is less suitable in our setting. These climatic conditions may affect political behaviour through channels other than wildfire, including agricultural production, household income, and local economic stress [6], making the exclusion restriction less plausible when they are used alone as instruments. We therefore construct an interaction-based instrument that is better aligned with our cumulative wildfire exposure measure, as detailed in Figure 7. Specifically, we interact meteorological conditions measured over the two years preceding the survey ($Tem_{v(i),t-2}$, $Prep_{v(i),t-2}$, and $Windspeed_{v(i),t-2}$) with a predetermined measure of land-cover flammability observed one year earlier ($I(LC)_{v(i),t-3}$). These factors jointly shape the likelihood and intensity of wildfires [7, 3]. This timing is intentional: weather is measured over the wildfire-exposure window to capture the conditions that generate fires, whereas land-cover flammability is measured earlier because it is a predetermined determinant of wildfire risk that may itself be altered by recent fires. In the estimation, we control separately for meteorological conditions and land-cover characteristics, so identification comes from the interaction-based component of weather variation in more combustible areas. These controls effectively absorb any alternative pathways through which these meteorological variables might influence political participation. As a result, the residual variation in the interaction terms is intended to isolate the component of weather variation that operates through wildfire incidence [8, 9, 10].

General concern on the credibility of the IV design

RC: ***However, I still think a far more fulsome theoretical explanation of the instrument's logic is important, particularly with detail and clarity on the individual elements that go into the instrument's constructions, and their individual and joint data-generating processes. In turn, linking this logic to a literature that has considered this specific treatment before would be useful. The credibility of these identification assumptions are the fulcrum of the paper, and so despite appreciating its other merits, this still feels like a necessary improvement before recommending for publication.***

AR: We thank the reviewer for this comment and we agree with the reviewer that the credibility of the IV design is central to the paper. In response to this broader concern, we have revised section **4.2 Estimation Method** to provide a more complete explanation of the instrument's logic, construction, and empirical support. First, we now situate our IV strategy more clearly in the wildfire literature and explain why alternative approaches are less suitable in our setting. Because our outcomes measure political participation over the year preceding the survey, it is not appropriate to leverage short-term (e.g., one week before specific events) fluctuations of weather conditions as instrumental variables directly as Pianta and Rettl [3], while weather or drought variables may affect political behaviour through channels other than wildfire, making the exclusion restriction less plausible when they are used directly as instruments.

Second, we clarify the construction and timing of each component of the instrument. Meteorological variables are measured over the wildfire-exposure window to capture the conditions that generate fires, whereas

land-cover flammability is measured earlier because it is a predetermined determinant of wildfire risk. We also explain how the flammability measure is constructed from land-cover categories and why it should be interpreted as predetermined landscape susceptibility to burning.

Third, we strengthen the discussion of the exclusion restriction and add supporting evidence. We explain why the interaction-based IV is intended to isolate the wildfire-generating component of climatic variation, and we report robustness checks excluding agricultural and built-up areas, as well as falsification and over-identification tests.

We again appreciate the reviewer's thoughtful feedback and hope the revisions have addressed the concerns raised. We would be glad to incorporate additional suggestions to further improve the manuscript if any.

References

[1] Allen Hicken. Clientelism. *Annual review of political science*, 14(1):289–310, 2011.

[2] René Lemarchand. Political clientelism and ethnicity in tropical africa:* competing solidarities in nation-building. *American political science review*, 66(1):68–90, 1972.

[3] Silvia Pianta and Paula Rettl. Global harms, local profits: How the uneven costs of natural disasters affect support for green political platforms. 2023.

[4] Hayato Umetani. Wildfires, pollution, and pregnancy outcomes: Evidence from lightning strikes. *Available at SSRN 4833440*, 2024.

[5] Luiz EOC Aragão, Liana O Anderson, Marisa G Fonseca, Thais M Rosan, Laura B Vedovato, Fabien H Wagner, Camila VJ Silva, Celso HL Silva Junior, Egidio Arai, Ana P Aguiar, et al. 21st century drought-related fires counteract the decline of amazon deforestation carbon emissions. *Nature communications*, 9(1):536, 2018.

[6] Jonathan Mellon. Rain, rain, go away: 194 potential exclusion-restriction violations for studies using weather as an instrumental variable. *American Journal of Political Science*, 69(3):881–898, 2025.

[7] W Matt Jolly, Mark A Cochrane, Patrick H Freeborn, Zachary A Holden, Timothy J Brown, Grant J Williamson, and David MJS Bowman. Climate-induced variations in global wildfire danger from 1979 to 2013. *Nature communications*, 6(1):7537, 2015.

[8] John T Gasper and Andrew Reeves. Make it rain? retrospection and the attentive electorate in the context of natural disasters. *American Journal of Political Science*, 55(2):340–355, 2011.

[9] Sarah Meier, Rob Elliott, and Eric Strobl. The regional economic impact of wildfires: Evidence from southern europe. *Journal of Environmental Economics and Management*, 118:102787, 2023.

[10] Mark Borgschulte, David Molitor, and Eric Yongchen Zou. Air pollution and the labor market: Evidence from wildfire smoke. *Review of Economics and Statistics*, 106(6):1558–1575, 2024.

Authors' Response to Reviews of

Fires and Votes: How Wildfires Drive Political Participation in Africa

Nature Communications, NCOMMS-25-68525B

RC: ***Reviewers' Comment***, AR: Authors' Response, ☐ Manuscript Text

We sincerely thank the editor and reviewers for their careful evaluation of our manuscript and for the opportunity to revise and resubmit. In response to the comments, we have revised the manuscript to clarify the instrumental-variable design and its remaining identification assumptions, added robustness checks for nonlinear weather controls, explicitly discussed the OLS–2SLS gap, and expanded the Discussion on the democratic implications of clientelistic disaster relief. Revisions in the manuscript are highlighted in BLUE for ease of reference.

Below, we provide detailed, point-by-point responses to each reviewer's comment.

1. Reviewer 1

RC: ***The authors appear to have misunderstood my request for "comment on the normative implications of the use of disaster relief for clientelistic purposes." When I referred to the normative implications of the clientelization of disaster relief, what I was referring to was the moral and democratic theory implications of these practices. If parties are using disaster relief to bribe voters, this is a form of corruption. It also undermines democratic representation, because it short-circuits voter evaluations of candidate competence and policy positions. It appears, therefore, that this practice is profoundly undemocratic and may lead to democratic backsliding. I invite the authors to reflect on this.***

AR: We thank the reviewer for this important clarification. We have rewritten the closing paragraph of the Discussion to note that, when disaster relief is used to influence voters, the practice can be viewed as a form of corruption that may weaken democratic representation. We also note that our data cannot directly test the clientelism mechanism, so we maintain a caveat to avoid overinterpretation.

The revised paragraph now appears in the Discussion section (final paragraph) and reads:

> Our findings are consistent with an instrumental, localized form of participation that may reflect clientelistic mobilization through disaster relief [1, 2]. While our data cannot directly test this mechanism, its moral and democratic-theory implications warrant explicit discussion. If parties use disaster relief to influence voters, this practice constitutes a form of corruption: it short-circuits voter evaluations of candidate competence and policy positions, thereby undermining democratic representation. To the extent that programmatic accountability is displaced by patronage, such practices are undemocratic and may contribute to democratic backsliding. Distinguishing genuine accountability pressures from clientelistic mobilization therefore remains an important agenda for future research.

2. Reviewer 2

RC: ***The authors have done a great job to address the challenging comments raised by reviews, including my own, throughout the review process and in this round in particular. This is a strong paper that I feel confident recommending for publication.***

AR: We thank the reviewer for this positive assessment and for the constructive engagement throughout the review process. The reviewer's earlier suggestions on the continuous distance measure, first-stage and reduced-form estimates, and the construction and timing of the IV helped us improve the transparency of the empirical analysis. We are grateful for the reviewer's time and expertise.

3. Reviewer 3

RC: ***I want to commend the authors for their engaged response to my comments, which have remained more challenging than the other reviewers. My reaction to this paper remains mixed, even after the additional (and substantial) revisions, but I do recognize that the other reviewers are on balance quite positive and that the authors have done serious empirical legwork here to address broader concerns. This empirical work does reassure me in part by offering a suite of robustness checks that help buttress some of the core claims.***

AR: We thank the reviewer for carefully evaluating the revised manuscript and for the valuable comments. We agree with the reviewer that the remaining concerns center on the credibility of the exclusion restriction and the interpretation of the OLS–2SLS magnitude gap, and we address these points directly below.

Comment 1: Credibility of the Exclusion Restriction

RC: ***Where I am less persuaded, this has to do with what I view as this revision's weaker effort to reassure the reader on the credibility of the instrument itself and the instrument's exclusion restriction. For example, the authors now write: "In the estimation, we control separately for meteorological conditions and land-cover characteristics, so identification comes from the interaction-based component of weather variation in more combustible areas. These controls effectively absorb any alternative pathways through which these meteorological variables might influence political participation." But this is not true mechanically. The controls absorb linear pathways associated with observables, but not unobservables and, critically, not the likely non-linear pathways associated with these observables. My intuition is that meteorological settings are precisely where we would expect to see non-linear effects of rainfall on crop yield, livestock, etc. and follow-on interactions with land-cover variables. In short, I think the paper needs to still be softened to make clear that there are substantial causal identification questions that remain unresolved.***

AR: We thank the reviewer for the valuable comment. In response, we have revised the manuscript to state the exclusion restriction more cautiously and to clarify what the controls can and cannot establish. The revised text now explains that separate controls for weather and land-cover characteristics address observed linear pathways, while unobserved and nonlinear weather-related channels cannot be fully ruled out. We have also added a sensitivity analysis using quadratic and cubic terms for temperature and precipitation.

First, to examine whether the estimates are sensitive to more flexible controls for weather conditions, we re-estimate the 2SLS specification by adding quadratic and cubic terms for temperature and precipitation. As reported in Extended Table B9, the estimated effects of wildfire exposure remain similar across these

specifications. This pattern reduces the concern that the estimates are primarily driven by simple nonlinear weather terms, while no finite set of polynomial controls can rule out all possible nonlinear or unobserved pathways.

Paragraph 3, Page 24

We carried out a series of robustness checks to assess potential threats to the exclusion restriction. As shown in Extended Table A6, the land-cover categories include not only natural environments, such as ice and snow, sandy areas, water bodies, forests, and grasslands, but also built-up areas and agricultural zones. One possible concern is that some land-cover categories correspond to agricultural or built-up environments, so the interaction term could partly reflect channels other than wildfire risk. For example, agricultural land may be associated with local production conditions, while built-up land may be correlated with settlement intensity and related social or economic activity. To assess whether our results are driven by these alternative channels, we conduct three robustness checks by excluding observations in agricultural areas, built-up areas, and both agricultural and built-up areas. As reported in Extended Tables B6–B8, the results remain consistent with the baseline estimates. A related concern is that temperature and precipitation may affect political participation through nonlinear non-wildfire channels, such as agricultural production, livelihood stress, or other weather-sensitive local conditions. To probe this possibility, we augment the baseline specification with quadratic and cubic terms for temperature and precipitation. The results, reported in Extended Table B9, remain similar to the baseline estimates. These checks reduce the concern that the estimates are driven by simple nonlinear weather effects, but they cannot rule out all unobserved or more complex nonlinear pathways.

Paragraph 4, Page 25

Taken together, these checks show that the estimates are stable across a range of specifications and do not reveal systematic inconsistencies. However, because they cannot exhaust all possible pathways, we interpret them as supporting the plausibility of the exclusion restriction rather than proving it.

Table B9, Page 48

Heterogeneity Analysis: Controlling for Quadratic and Cubic Terms of Temperature and Precipitation

Dept. Vars.	Voting	Contacting local govt.	Attending com. meetings	Collective issue-raising	Protest
	(1)	(2)	(3)	(4)	(5)
**Panel A: Adding Quadratic Terms**					
Wildfire	0.0675***	0.167**	0.388***	0.214***	0.0199
	(0.0257)	(0.0759)	(0.0929)	(0.0805)	(0.0678)
Observations	69,940	83,206	83,206	83,206	83,206
**Panel B: Adding Cubic Terms**					
Wildfire	0.0664**	0.166**	0.389***	0.213***	0.0200
	(0.0257)	(0.0770)	(0.0941)	(0.0817)	(0.0688)
Observations	69,940	83,206	83,206	83,206	83,206
**Panel C: Adding Both Cubic and Quadratic Terms**					
Wildfire	0.0808***	0.162**	0.390***	0.200**	0.000464
	(0.0284)	(0.0821)	(0.101)	(0.0874)	(0.0728)
Observations	69,940	83,206	83,206	83,206	83,206
Country-Year FE	Yes	Yes	Yes	Yes	Yes
Spatial Cut-off (miles)	20	20	20	20	20
Temporal Cut-off (months)	24	24	24	24	24

Note: This table reports the 2SLS estimation results of adding high-order term of annual temperature and precipitation as covariates. Robust standard errors are reported in parentheses. All specifications include country-year fixed effects. The spatial cut-off is 20 miles and the temporal cut-off is 24 months. *** p<0.01, ** p<0.05, * p<0.1.

Second, we have revised the language in the Methods section to reflect the reviewer's concern. The passage the reviewer quoted now reads:

Paragraph 2, Page 24

> Because our outcomes measure political participation over the year preceding the survey, it is not appropriate to leverage short-term (e.g., one week before specific events) fluctuations of weather conditions as instrumental variables directly as Pianta and Rettl [3], while using weather or drought directly as instruments is less suitable in our setting. These climatic conditions may affect political behaviour through channels other than wildfire, including agricultural production, household income, and local economic stress [4], making the exclusion restriction less plausible when they are used alone as instruments. We therefore construct an interaction-based instrument that is better aligned with our cumulative wildfire exposure measure. Specifically, we interact meteorological conditions measured over the two years preceding the survey ($Tem_{v(i),t-2}$, $Prep_{v(i),t-2}$, and $Windspeed_{v(i),t-2}$) with a predetermined measure of land-cover flammability observed one year earlier ($(I(LC)_{v(i),t-3}$). These factors jointly shape the likelihood and intensity of wildfires [5, 3]. This timing is intentional: weather is measured over the wildfire-exposure window to capture the conditions that generate fires, whereas land-cover flammability is measured earlier because it is a predetermined determinant of wildfire risk that may itself be altered by recent fires. In the estimation, we control separately for meteorological conditions and land-cover characteristics. These controls and a series of robustness checks substantially reduce concerns that the instruments capture non-wildfire channels related to weather or land cover. The remaining identifying variation is therefore tied to the interaction-based component of weather variation in more combustible areas, although the exclusion restriction ultimately remains an identifying assumption [6, 7, 8].

We believe the manuscript now presents its causal claims with appropriate caution. The exclusion restriction is explicitly noted as an identifying assumption, and the remaining uncertainty is acknowledged transparently.

Comment 2: The OLS–2SLS Gap

RC: ***In addition, in reading the paper another time, the substantial gap between the magnitude of OLS and 2SLS estimates surprised me. (I should have seen this earlier!) I do think the authors should add some explicit recognition of this, and provide some speculation on why there is severe downward bias in the OLS.***

AR: We thank the reviewer for this observation, which we agree deserves explicit discussion. Table 1 reports the OLS and 2SLS estimates side by side. The corresponding estimates are 0.0045 and 0.0728 for voting, 0.0433 and 0.1490 for contacting local government, 0.0668 and 0.3750 for attending community meetings, 0.0486 and 0.2080 for collective issue-raising, and 0.0127 and 0.0373 for protest. We have added a footnote in the Results section (accompanying Table 1) to acknowledge these OLS–IV differences explicitly and to offer two cautious interpretations: possible downward OLS bias and the local nature of the IV estimand. The revised text therefore recognizes the gap while avoiding an interpretation of the IV estimates as population-wide ATEs.

Footnote 1, Page 7

> The OLS–IV gap can be interpreted in light of two complementary considerations: possible downward bias in OLS and the local nature of the IV estimand. OLS may understate the effect of wildfire exposure if wildfire-prone areas are negatively correlated with unobserved determinants of political participation,

> such as remoteness, weaker electoral integration, or lower access to public institutions [9, 10]. In addition, because the instrument shifts wildfire exposure through meteorological fire-risk variation interacted with predetermined land-cover flammability, the IV estimate is closer to a local average treatment effect (LATE) for observations whose wildfire exposure is shifted by the instrument, rather than a population-wide ATE.

We also note that the OLS estimates are smaller than the corresponding 2SLS estimates across all five outcomes. This pattern is compatible with attenuation from measurement error in the wildfire exposure measure, although we treat this as a possible explanation rather than direct evidence. The OLS and 2SLS estimates generally point in the same direction, and both show no clear effect on protest. Thus, while the estimated magnitudes differ, the broad directional pattern is not unique to the 2SLS specification.

We thank the reviewer for encouraging us to make this point explicit. The revised footnote now acknowledges the OLS–IV differences and frames their interpretation cautiously.

Closing

RC: ***With these issues made more transparent, I am comfortable deferring to the more positive assessments of the other reviewers, largely because I do think the empirical setting here is understudied and there is real value in careful empirical work in non-US/Europe on this topic.***

AR: We thank the reviewer for this assessment and for encouraging us to state the remaining identification limits more explicitly. In the revised manuscript, we have clarified the exclusion-restriction assumption, added sensitivity checks for higher-order weather terms, and discussed the OLS–2SLS differences more directly. We hope these revisions make the scope and limits of the evidence clearer.

Again, we are grateful to all reviewers for their time, careful reading, and thoughtful suggestions, which have helped us improve the manuscript.

References

[1] Allen Hicken. Clientelism. *Annual review of political science*, 14(1):289–310, 2011.

[2] René Lemarchand. Political clientelism and ethnicity in tropical africa:* competing solidarities in nation-building. *American political science review*, 66(1):68–90, 1972.

[3] Silvia Pianta and Paula Rettl. *Global Harms, Local Profits: How the Uneven Costs of Natural Disasters Affect Support for Green Political Platforms*. Harvard Business School, 2023.

[4] Jonathan Mellon. Rain, rain, go away: 194 potential exclusion-restriction violations for studies using weather as an instrumental variable. *American Journal of Political Science*, 69(3):881–898, 2025.

[5] W Matt Jolly, Mark A Cochrane, Patrick H Freeborn, Zachary A Holden, Timothy J Brown, Grant J Williamson, and David MJS Bowman. Climate-induced variations in global wildfire danger from 1979 to 2013. *Nature communications*, 6(1):7537, 2015.

[6] John T Gasper and Andrew Reeves. Make it rain? retrospection and the attentive electorate in the context of natural disasters. *American Journal of Political Science*, 55(2):340–355, 2011.

[7] Sarah Meier, Rob Elliott, and Eric Strobl. The regional economic impact of wildfires: Evidence from southern europe. *Journal of Environmental Economics and Management*, 118:102787, 2023.

[8] Mark Borgschulte, David Molitor, and Eric Yongchen Zou. Air pollution and the labor market: Evidence from wildfire smoke. *Review of Economics and Statistics*, 106(6):1558–1575, 2024.

[9] Enrico Cantoni. A precinct too far: Turnout and voting costs. *American Economic Journal: Applied Economics*, 12(1):61–85, 2020.

[10] Joshua J Dyck and James G Gimpel. Distance, turnout, and the convenience of voting. *Social Science Quarterly*, 86(3):531–548, 2005.
